# Multidirectional color palette of electrochromic metal–organic frameworks

**DOI:** 10.1093/nsr/nwaf326

**Published:** 2025-08-12

**Authors:** Cha Li, Jinli Zhang, Yudong Lian, Kai Zhang, Hao Zhang, Lin Xu, Yanghe Liu, Feifan Lang, Jiandong Pang, Xian-He Bu

**Affiliations:** School of Materials Science and Engineering, Smart Sensing Interdisciplinary Science Center, Collaborative Innovation Center of Chemical Science and Engineering, Nankai University, Tianjin 300350, China; School of Materials Science and Engineering, Smart Sensing Interdisciplinary Science Center, Collaborative Innovation Center of Chemical Science and Engineering, Nankai University, Tianjin 300350, China; School of Materials Science and Engineering, Smart Sensing Interdisciplinary Science Center, Collaborative Innovation Center of Chemical Science and Engineering, Nankai University, Tianjin 300350, China; School of Materials Science and Engineering, Smart Sensing Interdisciplinary Science Center, Collaborative Innovation Center of Chemical Science and Engineering, Nankai University, Tianjin 300350, China; School of Materials Science and Engineering, Smart Sensing Interdisciplinary Science Center, Collaborative Innovation Center of Chemical Science and Engineering, Nankai University, Tianjin 300350, China; School of Materials Science and Engineering, Smart Sensing Interdisciplinary Science Center, Collaborative Innovation Center of Chemical Science and Engineering, Nankai University, Tianjin 300350, China; School of Materials Science and Engineering, Smart Sensing Interdisciplinary Science Center, Collaborative Innovation Center of Chemical Science and Engineering, Nankai University, Tianjin 300350, China; School of Materials Science and Engineering, Smart Sensing Interdisciplinary Science Center, Collaborative Innovation Center of Chemical Science and Engineering, Nankai University, Tianjin 300350, China; School of Materials Science and Engineering, Smart Sensing Interdisciplinary Science Center, Collaborative Innovation Center of Chemical Science and Engineering, Nankai University, Tianjin 300350, China; Haihe Laboratory of Sustainable Chemical Transformations, Tianjin 300192, China; School of Materials Science and Engineering, Smart Sensing Interdisciplinary Science Center, Collaborative Innovation Center of Chemical Science and Engineering, Nankai University, Tianjin 300350, China; State Key Laboratory of Elemento-Organic Chemistry, Frontiers Science Center for New Organic Matter, College of Chemistry, Nankai University, Tianjin 300071, China; Haihe Laboratory of Sustainable Chemical Transformations, Tianjin 300192, China

**Keywords:** zirconium metal–organic frameworks, electrochromism, topology engineering, linker installation, chemical palette

## Abstract

Electrochromic metal–organic frameworks (MOFs) combine advantages from both inorganic/organic electrochromic materials by enabling stable structures/performances as well as tunable functionalities. Their current design and synthesis, however, are inherently complicated, as they often involve amendments to the chromogenic components and/or MOF structures. Inspired by reticular chemistry, we herein demonstrate a multidirectional ‘color palette’ based on the colorful electrochromic behaviors across a total of 40 zirconium-based MOFs via systematically combining diverse naphthalene diimide (NDI)-based primary linkers (R-groups) and auxiliary linkers (X-groups), namely the NKM-908-R/NKM-906-R series (**csq**/**scu** topology) and their corresponding NKM-908-R-TPDC-X/NKM-906-R-TPDC-X derivatives. A new broad color gamut over these robust MOF thin films was showcased via systematical crystal engineering and was thoroughly investigated. The benefits include enhancing the primary set of ‘colors’ from altering peripheral R-groups without redesigning the NDI core, introducing different X-groups as the secondary sets of ‘colors’ and topology adjustments to incorporate more X-linkers for intensification of the latter. Together, such designable color-mixing/changing sequences successfully mimicked the use of the routine color palette with molecular-level precision. This work not only holds great potential for further extension, but also provides unique insights into the development of next-generation electrochromic devices.

## Teaser

Mixing colors in Zr-MOFs! Tuning the types and ratio of primary & auxiliary redox-active linkers enables an electrochromic color palette at molecular-level precision, where the hue/intensity of primary & secondary colors can be rationally customized.

## INTRODUCTION

Electrochromism—defined as being capable of changing color due to an external electric field—is fundamental to modern intelligent devices for applications such as smart displays, dimming windows, adaptive surfaces and even energy conservation/storage [1–7]. In general, inorganic electrochromic materials have advantages due to their defined structure, better reversibility, high robustness and short responsive time, but behave relatively poorer regarding discoloration [8–11]. The more designable and processable organic electrochromic materials show richer color change, but exhibit weaker durability and uniformity than the former [12–16]. Decades of intensive studies have been devoted to electrochromic materials and advancements that may combine those advantages in state-of-the-art technologies are still being sought [17–20]. One highly anticipated competitor is the metal–organic framework (MOF)—a class of inorganic–organic hybrid materials developed at the end of the last century [21–23]. These porous crystalline materials typically possess framework-like porous periodical structures with ultra-high surface area, an ordered array of active sites and, most importantly, stable but tunable pore architectures and functionalities [24–26]. It is therefore possible to introduce metal sites with multiple oxidation states and/or redox-active organic moieties (either on linkers or guest molecules) into MOFs, while simultaneously achieving a reasonable electrolyte-transfer rate and redox properties [27–30]. For instance, naphthalene diimide (NDI)-based aromatic linkers have been widely applied in functional MOFs due to their strong electron-deficient properties and they induce charge transfer from electron donors. This further allows customization of their abundant chromatic behaviors from rationally altering the MOF structures and/or the physical/chemical surroundings [31]. Some leading electrochromic MOFs have been established as potential solutions to modern technologies [32–34], yet are admittedly insufficient compared with other well-reported applications of MOFs (e.g. catalysis, sensing, adsorption/separation, compound delivery) regarding systematic exploration [35–38].

Several pioneering studies have explored the potential of MOF-based platforms as efficient electrochromic materials [39–42]. Among these, zirconium-based MOFs (Zr-MOFs) have emerged as particularly promising candidates due to their exceptional robustness and tunability, especially with regard to network topology [43–46]. Also, the strong coordination bonds between Zr^4+^ ions and carboxylate-based linkers offer settled frameworks during redox processes under various conditions [47,48]. While these studies have led to effective modifications to the electrochromic properties of Zr-MOFs (or other MOFs, polymers, etc.) constructed from chromogenic linkers, the majority focuses on structural modifications of the chromophore cores that sometimes alter the size and/or the shape of the linkers, alongside the MOF architectures yielded [49–51]. This adds complexity—from a practical production and application perspective—to the design/synthetic procedures and post-synthetic modifications during the fine-tuning of the properties, especially with regard to the systematic/uniform introduction of functional groups/units. In this context, one primary goal in this field is to facilitate the high-performance and adjustable electrochromic behaviors of MOFs while minimizing synthetic/structural variations.

Herein, we report the first MOF platform for multidirectional electrochromism achieved via a large library of pure MOF thin films in which their electrochromic behaviors have been remarkably and precisely varied yet with only minor alterations to the MOF structure and synthetic condition. Specifically, a total of 40 different MOFs (and their corresponding thin films) have been systematically synthesized within the NKM-908-R/NKM-908-R-TPDC-X and NKM-906-R/NKM-906-R-TPDC-X series by utilizing all feasible combinations of selected primary and auxiliary linkers. Those include five different NDI-based tetracarboxylate NDTB-*R* primary linkers (NDTB-*R* = 5',5''''-(1,3,6,8-tetraoxo-1,3,6,8-tetrahydrobenzo[lmn][3,8]phenanthroline-2,7-diyl)bis(2'-R-[1,1':3',1''-terphenyl]-4,4''-dicarboxylate), *R* = H, Me, OMe, F or OH; Figs S1–S6), with/without three different dicarboxylate TPDC-*X* auxiliary linkers that could be post-synthetically installed [52,53] into the coordination-unsaturated pockets of the MOFs (TPDC-*X* = 4,4'-(X)dibenzoate, *X* = AN, Py or TDA (i.e. anthracene-9,10-diyl, pyridine-2,5-diyl or benzo[c][1,2,5]thiadiazole-4,7-diyl functional units; Fig. S7), respectively. Upon the application of potentials, two sets of colors originated from the R-linkers and X-linkers, respectively, which showed exceptionally clear and diverse changing/mixing sequences over a wide hue–saturation–lightness (HSL) space.

We demonstrated and comprehensively investigated—for the first time in MOF materials—the harmonious trinity of crystal engineering that constructively achieved the multivariant control of electrochromism in the MOF materials as if using a routine color palette in daily life. The possible modifications involved the strength of the ‘primary’ set of colors (varying R-groups), the types of ‘secondary’ colors (varying X-linkers) and the strength of the ‘secondary’ sets of colors (changing MOF topology, i.e. the quantity of X-linkers), plus great potential for subsequent extension with molecule-level precision. Also remarkably, the demonstrated color-mixing/changing sequences are intuitive and fully predictable, which is rarely reported and of significance for real usage. Given the robust preparation procedure and film performance, such a crystalline electrochromic platform is considered a promising multi-domain ‘color palette’ and holds great value for customized applications including anti-counterfeiting and smart electronics.

## RESULTS AND DISCUSSION

### NKM-908-R series

**Figure 1.**
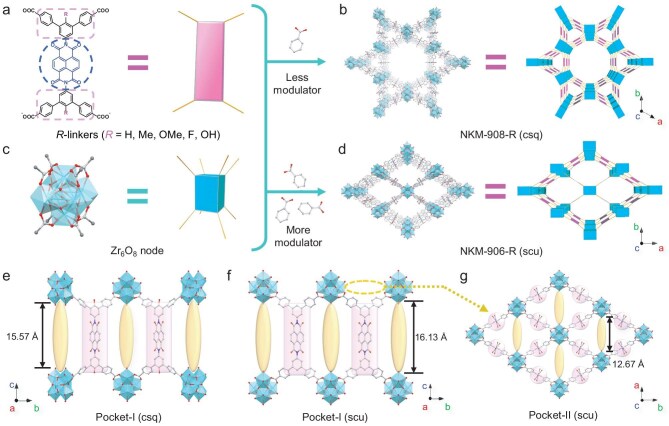
Single-crystal structures and the corresponding simplified units/topologies of (a) R-linker (pink atoms: simplified R-groups; blue frame: NDI core; pink frames: ‘modifiable peripheral zones’), (b) NKM-908-OH, (c) Zr_6_O_8_ nodes and (d) NKM-906-H. Hydrogen atoms were omitted for clarity. (e) One type of coordination-unsaturated pocket within NKM-908-R. (f, g) Two types of coordination-unsaturated pockets within NKM-906-R. The Zr···Zr distances were measured after subtracting the crystallographic van der Waals radii of terminal Zr atoms (2.3 Å) at either end.

The modification of NDI-containing linkers (R-linkers) represents the first step in regulating the electrochromism of MOFs in this work. It specifically emphasizes modifying the peripheral functional groups (R-groups) in the ‘modifiable peripheral zones’ located at either end of these tetratopic carboxylates while maintaining the chromogenic NDI cores unaltered (Fig. 1a) to achieve a consistent shape and size among all the R-linkers. Four new NKM-908-*R* products (*R* = H, Me, F or OH) were successfully synthesized under similar conditions, followed by successful determination of the single-crystal X-ray diffraction (SCXRD) structures of NKM-908-Me and NKM-908-OH (Table S1). As the powder X-ray diffraction (PXRD) patterns of all as-synthesized NKM-908-*R* variants hardly showed any significant differences, it was concluded that the entire NKM-908 series shared the same (4,8)-connected **csq** topology (Fig. 1b and c). Their PXRD patterns exhibited signs of neither phase change nor degradation, which confirmed their robustness subject to solvent-exchange and thermal activation (Figs S8–S12). Elemental analyses (EAs) further confirmed their purity from the perspective of elemental composition (Table S2). Volumetric N_2_ adsorption measurements at 77 K further validated these findings via demonstrating their similar Type-IV adsorption behavior and Brunauer–Emmett–Teller (BET) surface area ranging between 2101 and 2925 m^2^ g^−1^ (Fig. S13a and Table S3). Corresponding pore-size distributions supported the presence of mesoporous channels (2.7-nm hexagonal apertures) and microporous channels (1.2-nm triangular apertures) within these stable Zr-MOFs, which could favor fast electrolyte transfer within the material (Fig. S13e). Also, regarding the pristine R-linkers, the color of the NKM-908-*R* crystals varied from almost white to yellow upon changing the R-groups and was predicted to serve a role in the following fine-tuning of their electrochromic properties.

### NKM-906-R series

The second direction of regulation involved the rational application of reticular chemistry, the significance of which lies in the tailored functionalities of the porous materials from understanding and manipulating the connections between their building blocks. By simply modifying the number of modulators during the synthesis of NKM-908-*R*, a series of NKM-906-*R* (*R* = H, Me, OMe, OH and F) were afforded with exceptional purity (NKM-906-H was previously reported as Zr-NDI-**scu**-MOF [41]; Fig. 1d). Analogous to the NKM-908-*R* series, a shared (4,8)-connected **scu** topology among all these NKM-906-R products was therefore reasonably deduced from the single-crystal structure of NKM-906-OMe (Table S1) and highly matched PXRD patterns within the series, along with well-matched EA results (Table S2). They also demonstrated good stability after desolvation though observing slightly shifted reflection positions due to the flexibility of the rhombic channels (Figs S14–S18). Consistent permanent porosity and Type-I adsorption behaviors of these activated MOFs were evidenced by their volumetric N_2_ sorption isotherms at 77 K (BET surface area ranging between 287 and 533 m^2^ g^−1^), as well as the similar pore-size distribution that was calculated to be ∼1.2 nm in diameter (Fig. S19a and e, and Table S4).

Mild color intensification was also observed across the NKM-906-R products upon varying the R-groups and, surprisingly, it matched well with the corresponding NKM-908-*R* containing the same substituents. This observation might indicate a negligible difference in their intrinsic color and potentially the electrochemical performance by only altering between the two topologies. But, on the flip side, NKM-906-R featured two forms of coordination-unsaturated pockets between the adjacent Zr_6_O_8_ nodes with similar size owing to the **scu** topology (Zr···Zr distances roughly being 12.67/16.13 Å along the *a*-/*c*-axes within the rhombic or elongated-hexagonal channels, respectively; note that the former is flexible [54]; Fig. 1f and g), whereas NKM-908-*R* had only one such configuration within their **csq** nets (Zr···Zr: 15.57 Å along the *c*-axis; Fig. 1e). This led to a notable distinction in their capability to be post-synthetically installed with suitable functional linkers within their pockets, especially the quantity of which shall affect the initial color and electrochromic behavior of the resulting MOFs.

## NKM-908-R-TPDC-X series and NKM-906-R-TPDC-X series

**Figure 2.**
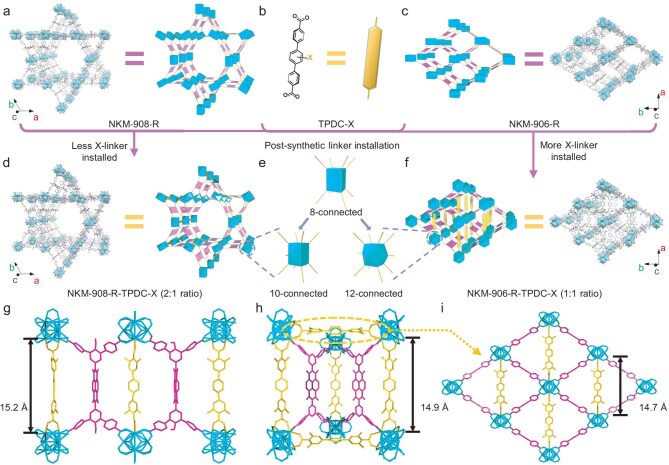
Structures and corresponding simplified units/topologies of (a) NKM-908-OH, (b) X-linker, (c) NKM-906-H, (d) NKM-908-OMe-TPDC-4F, (e) different connecting modes of Zr_6_O_8_ nodes and (f) NKM-906-H-TPDC-4F. Illustrative crystal structures of (g) pocket-I within NKM-908-OMe-TPDC-4F, (h) pocket-I and (i) pocket-II within NKM-906-H-TPDC-4F. Hydrogen atoms were omitted and only one conformation of each disordered part is shown for clarity. The numbers in brackets represent the ideal *n*_R_:*n*_X_ molar ratios after installation.

Previous Zr-MOF-based studies have demonstrated the excellent designability and extensibility of the linker-installation strategy for largely enriching structure diversity and functionality [52–54], especially regarding those materials that may be challenging to synthesize conventionally. Building on this, three different TPDC-X linkers (*X* = TDA, AN or Py) shared a similar molecular length of ∼15.5 Å (O···O distance between terminal oxygen atoms; van der Waals radius: 1.52 Å) were selected as suitable candidates for this study (Fig. 2b). This strategy allowed their controlled integration into the structural pockets of both MOF series, preserving the analogous framework architecture across the derivatives while endowing distinct electrochemical responsiveness. In other words, this represented the third means of regulating the electrochromic behavior of these MOFs by introducing sites with diverse redox activity conveniently.

A total of 15 MOFs were prepared to accomplish the NKM-908-R-TPDC-X series based on the complete permutations of combining those abovementioned five R-linkers and three TPDC-X linkers, while also minimizing the variations in their synthetic conditions. According to the single-crystal structure of NKM-908-OMe-TPDC-4F (Table S1), the Zr···Zr distance in the saturated pockets was 15.2 Å (Fig. 2g). PXRD analyses revealed the pattern consistency of the as-synthesized MOFs with NKM-908-OMe-TPDC-4F [42] and confirmed their high purity together with the EA results (Table S2), indicating their isostructural nature ((4,10)-connected topology; Fig. 2d and e, and Figs S20–S34). Such an installation resulted in a theoretical molar ratio between the R-linkers and X-linkers (*n*_R_:*n*_X_) of 2:1. As a result, the volumetric N_2_ sorption isotherms of NKM-908-R-TPDC-X demonstrated a universal decrease in their capacity, i.e. specific surface area, compared with the parent NKM-908-*R* due to the occupancy of the X-linkers within the channels (for ∼10%–25%; Fig. S13b–d and f–h, and Table S3). Yet their pore-size distribution hardly showed any significant changes, especially to the mesopores (Figs S13f–h). Also notably, only MOFs with TPDC-TDA installed exhibited slightly more yellowish colors, whereas the others remained as pale as their parent MOFs.

Subsequently, the NKM-906-R-TPDC-X series was launched by innovatively synthesizing 15 derivatives by following all of the different combinations of primary/auxiliary linkers. Recognizing that these crystals were undersized for reliable SCXRD data collection, the edge-cutting 3D electron diffraction was therefore professionally conducted. The single-crystal structure of NKM-906-H-TPDC-4F was successfully determined (Fig. 2f and Table S1) as an illustration in comparison with NKM-908-OMe-TPDC-4F. Starting from the **scu**-net of NKM-906-H (Fig. 2c), the TPDC-4F linkers replaced those coordinated water molecules facing the rhombic channels and bridged neighboring Zr_6_O_8_ nodes along the *a*-axis (with the corresponding Zr···Zr distance slightly increased to 14.7 Å; Fig. 2i). While this aspect of the linker installation resembled that of the previously reported Zr-NDI-BPy-**sco**-MOF [41], their key difference lay in the additional TPDC-4F linkers coordinating between the Zr_6_O_8_ nodes along the *c*-axis inside the elongated-hexagonal channels of NKM-906-H-TPDC-4F (reduced Zr···Zr distance to 14.9 Å; Fig. 2h). This led to a (4,12)-connected framework and a more prominent theoretical *n*_R_:*n*_X_ of 1:1 (Fig. 2e and f). The PXRD patterns of all NKM-906-R-TPDC-X derivatives showed minimal differences (Figs S35–S49) alongside good crystallinity and purity. Also, in contrast to NKM-908-R-TPDC-X, a general increase in the volumetric N_2_ adsorption capacity (i.e. surface area) was observed for NKM-906-R-TPDC-X (1166–1914 m^2^ g^−1^; Fig. S19b–d and f–h, and Table S4) compared with their parent NKM-906-R. This was attributed to the auxiliary linkers serving as structural pillars within the flexible rhombic channels that obstructed their dynamic ‘breathing’ behavior upon desolvation, which was beneficial in providing a better and steady electrolyte-transfer rate during the redox processes.

### MOF thin films

The abovementioned 40 different NKM-908-R/NKM-906-R/NKM-908-R-TPDC-X/NKM-906-R-TPDC-X were fabricated onto the surface of indium tin oxide (ITO) glass plates *in situ* as thin films, respectively, to achieve better particle-size consistency and electrochromic performance (Fig. S50). For those that required linker installation, the process itself was robust and efficient enough to be directly carried out on the thin films of parent MOFs compared to using normal crystals, and with no corrosion to either the films or the ITO electrodes. The initial colors of these films varied from transparent to yellow, which matched well with the corresponding crystals that were prepared normally. To precisely characterize the synthesized thin films, these microcrystals were gently removed from the ITO glass for general analyses. PXRD proved their phase purity and good crystallinity compared to the normal crystals. ^1^H nuclear magnetic resonance (^1^H NMR; Figs S51–S90) spectra demonstrated the purity of the MOFs, as well as accurately confirming *n*_R_:*n*_X_ after digestion, which aligned well with the theoretical ratio for full installation of the coordination-unsaturated pockets (∼2:1 for 908-X series and 1:1 for 906-X series). Further, the doubling of the relative number of X-linkers in NKM-906-R-TPDC-X compared with that in NKM-908-R-TPDC-X was expected to lead to a noticeable modification to the electrochromic behaviors. According to the scanning electron microscopy (SEM) images, the rod-like MOF crystals were grown and distributed evenly on the ITO glass surface, forming uniform and dense layers (the thickness sat mostly between 4 and 7 μm) with the majority of crystals orienting relatively upwards (Figs S8–S12, S14–S18 and S20–S49). Compared with liquid-phase dispersions (random particle sizes and aggregation), such film deposition allowed enhanced interaction between the MOF crystals and the ITO surface, which largely optimized the electronic transfer, while also inhibiting possible exfoliation/non-uniformity regarding practical production and usage.

### Electrochromic behaviors

**Figure 3.**
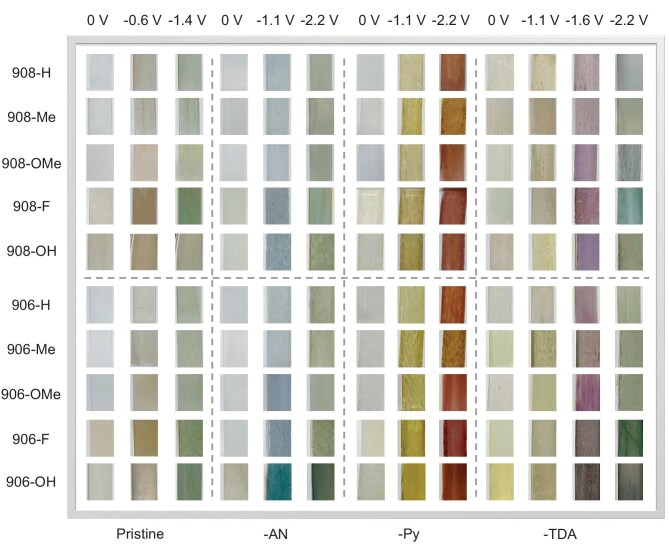
Collections for all 40 MOF thin films at representative potentials (colors) during electrochromic processes (photographed through the electrolytic cell). Note that these representative potentials are not those when the redox events started to occur, but the ‘turning points’ of absorption behaviors, as they were more intuitive for visual observation (see Fig. 4).

**Figure 4.**
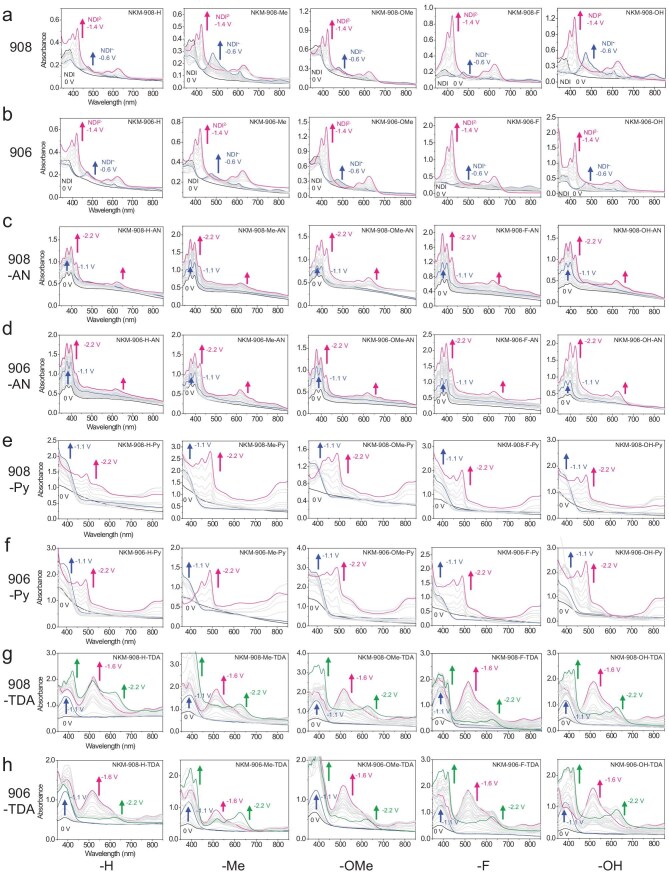
*In situ* UV–vis absorption spectra of all 40 MOF thin films during electrochromic processes. Spectra at turning points (i.e. when the characteristic absorption maximized) are highlighted to correspond to the presented colors in Fig. 3. The corresponding transmittance spectra and corresponding optical contrast (Δ*T*) values are shown in Fig. S95.

The electrochemical characteristics of these films were revealed by respectively applying them as the working electrodes in cyclic voltammetry (CV) measurements by using a three-electrode electrochemical cell (0.1 M [(*^n^*Bu)_4 _N]PF_6_/DMF as the electrolyte). All 40 MOF films demonstrated well-behaved quasi-reversible reduction events, together with conspicuous and diverse electrochromic behaviors (Fig. 3). Transmission UV–vis spectroscopy was applied simultaneously along the processes to monitor the changes in optical absorption. Table S5 summarizes the representative potentials for these films within a voltage window of 0 to −2.2 V (for brevity, MOFs are labeled in Arabic numbers and redox stages are in Roman), as these were the ‘turning points’ of their absorption behaviors according to the UV–vis spectra (Fig. 4). The corresponding reference pure colors of all 40 MOFs at each redox state were also acquired from these photographs (Fig. S91) and digitized into coordinates based on two distinct but globally recognized color models: the RGB (Red-Green-Blue; designed for digital displays) and HSL (more intuitive than RGB for how humans perceive color) [55,56]. This was aimed to provide standardized reference for these colors and to minimize ambiguity on account of the individual differences (naked eyes, displayer settings, etc.) and subjectivity in describing colors and their variations.

The investigation started with the 10 thin films of parent NKM-908-*R* (labeled as **1–5**; in the order of –H/–Me/–OMe/–F/–OH for R-groups, and similarly for all subgroups of five similar MOFs hereinafter) and NKM-906-R (**6–10**), which possessed close parallels between their CV curves (Fig. S92a and b). Two distinct one-electron redox couples were shown (maximized at around −0.55 V/−0.4 V and −0.9 V/−0.7 V), which consequentially arose from the reduction/oxidation of NDI moieties during the process (NDI ↔ NDI^•−^ ↔ NDI^2−^; Fig. S93a). This was consistent with previous reports and the CV results of pure H_4_NDTB-R linkers in the solution phase (Fig. S94) and demonstrated the de-embedding of the electrolyte cations. Meanwhile, as designed, the Zr_6_O_8_ clusters only served as structural nodes due to the redox-inactive *d*^0^ electronic configuration of Zr^4+^ ions (much higher electronic band gap compared with organic linkers). This was again supported by the examinations on the respective pure R-linkers, which all possessed two pairs of peaks that closely matched their corresponding MOFs. Accordingly, the color changes of these 10 thin films basically followed a similar trend during the process: white or pale yellow ↔ yellow–orange or brown ↔ green. Each two MOFs constructed from the same R-linkers showed nearly identical colors, indicating that switching between the **csq/scu** topology hardly affected the electrochromic behavior during this stage.

The four thin films with R-groups of -H or -Me showed minuscule color differences between one another at each state. UV–vis absorption spectra (Fig. 4a and b) revealed that, under the neutral state, the strong absorption bands at <400 nm dominated and were reflected as the initial pale colors of the films, which had arisen from the NDI-originated π→π^*^ transitions. The colors then shifted toward pale yellow–orange/brown under the NDI^•−^ state, which was attributed to the new absorption peak with *λ_max_* at ∼475 nm (cyan absorption; broad single peak characteristically). This absorption band maximized at about −0.6 V and was gradually quenched at further external potentials. This accompanied the introduction of two new absorption bands at ∼400–450 nm (violet–blue absorption) and ∼550–675 nm (yellow–red absorption; two adjacent peaks characteristically, likely indicating n→π^*^ transition), which resulted in the greenish colors at the NDI^2−^ state.

While the six MOFs with –OMe, –F or –OH groups also followed this trend, they exhibited more darkened/strengthened colors throughout the process. Evidence from UV–vis absorption/transmittance spectra (Fig. 4a and b, and Fig. S95a and b) pointed out the overall stronger absorption (or lower transmittance) of these six MOFs under given potentials. In fact, while the optical contrast (Δ*T*) values were limping from –H (35.5/36.9%) to –Me (38.8/38.7%) then to –OMe (39.7/43.1%), drastic increases were shown upon using R-linkers with –F (55.7/55.6%) and –OH (65.6/68.4%). From the HSL coordinates point of view, they presented generally lower lightness values. This was attributed to the darker MOF crystals, i.e. their darker R-linkers in the first place, that presumably originated from the hyperchromic effect of these three R-groups on the ‘modifiable peripheral zones’ as auxochromes (trend: –H ≈ –Me < –OMe << –F < –OH). It is possibly due to the increasing electro-donating effect of these functional groups to the zone that enhanced the absorption across different MOFs. In other words, it still follows the consensus that the functionalization of imide nitrogen atoms should have little effect on the optical/electrochemical behaviors of the NDI core itself [31]. Yet, color intensification to the bleached state and throughout the electrochromic processes of these MOF materials was successfully achieved via varying the peripheral R-groups of the NDI-containing primary linkers. And now, figuratively, such an indirectly tuned electrochromism represents the primary set of ‘colors’ and its corresponding changes in this ‘color palette’.

The remaining 30 NKM-908-R-TPDC-X and NKM-906-R-TPDC-X thin films together exhibited a rather richer color gamut from their additional X-linkers. In essence, additional redox peak pairs that corresponded to the reduction/oxidation of the X-linkers (Fig. S96) were observed in each CV curve (Figs S92c–h), bringing remarkably altered electrochromic behaviors, particularly in hue variation. The spectral contributions attributed to these X-linkers, i.e. their colors, were denoted as the secondary set of ‘colors’ introduced into this ‘color palette’. With the different redox potentials of these X- linkers, these secondary colors physically (or ‘spectroscopically’, to be more precise) blended with the primary electrochromic sequence (from the R-linkers) across the various potential regions, leading to highly distinct and customizable color-transition modes among these MOFs. Of special note was that, in most of the CV curves, the peak pairs for NDI/NDI^•−^ did exist but were often difficult to observe due to their relatively low peak heights. Also, due to the installation of extra linkers into the porous frameworks, the potentials for NDI redox events also underwent slight shifts relative to those of their parent MOFs. An illustrative comparative experiment using drop-cast films of linker inks (physically mixing the ground powder of H_4_NDTB-OMe and H_2_TPDC-Py) replicated the electrochromic behavior of NKM-908-OMe-TPDC-Py (Fig. S97). This result supported the fact that such a Zr-MOF-based color-mixing platform functions as a blender of two ‘individual colors’, with each generated and controlled by the respective redox-active organic moieties. Yet, importantly, these linker mixtures exhibited clearly inferior stability or uniformity compared with MOF films under the same conditions (possibly attributed to the common limitations of organic solids: aggregation, poor adhesion, etc.). This underscores the advantage of MOF and linker installation, as the two linkers are distributed evenly across the periodic crystalline structure. Together with better surface adhesion from *in situ* growth, this platform achieves color mixing at molecule-level dispersion rather than at the ‘particle level’, thereby offering better color quality and practicality for applications.

More specifically, the MOF with TPDC-AN installed exhibited the situation of having a relatively earlier onset of the X-linker redox event (Fig. 4c and d, **11–15** for 908 and **16–20** for 906 series; Fig. S93b for proposed redox mechanism). Initially, they presented nearly identical colors at neutral states to those of their parent MOFs. Their color then transitioned to a blueish hue (−1.1 V) before the observation of the yellow coloration of the MOFs, which was associated with the overall increased absorption (more rapid below 420 nm; characteristic for TPDC-AN for the π→π^*^ transitions of anthracene units). Notably, the mixture of ‘light yellow’ and ‘blue’ spectra eventually led to the grayish-blue colors of these MOFs at the NDI^•−^ state, which was consistent with the color blending predicted by the RGB model. Upon reaching the second redox event for NDI, they presented the greenish colors of the parent MOFs but eventually became more ‘brownish’ (−2.2 V), especially for those that possessed apparent blue colors at the previous stage. This was contributed from the overall lifted absorption across the tested region. As anticipated, changing R-groups led to deeper colors among these MOFs and followed the abovementioned trend in the parent MOFs. Also, NKM-906-X-TPDC-AN displayed more pronounced bluish/greenish colors due to having more X-linkers than NKM-908-X-TPDC-AN (overall lower lightness), which was evidenced by the relatively stronger characteristic absorption in the spectra.

In a similar fashion, MOFs installed with TPDC-Py linkers were generally pale at the neutral state (Fig. 4e and f, **21–25** for 908 and **26–30** for 906 series; Fig. S93c). As TPDC-Py possessed a relatively later redox event, the colors of these MOFs firstly went yellowish upon the first NDI redox event until −1.1 V, but were visually brighter (yellower) than the parent MOFs (which were more brownish). This was reflected by the strongly lifted absorption at <450 nm compared with those of their parent MOFs and was likely due to the introduction of TPDC-Py. Regarding the color coordinates, generally lower B-values (RGB) were shown for Pys, whereas their H-values (HSL) moved toward yellow zones (roughly 50°–70°) rather than orange zones (roughly 30°–50°). Upon applying further potentials, color shifts toward crimson or reddish-brown were subsequently observed for these materials due to the overlapped redox events of TPDC-Py and NDI^2−^ that was essentially blending red with various shades of green, respectively. The broad absorption band at this stage covered mostly >400–550 nm (violet–yellow absorption; characteristic for TPDC-Py, which presumably arose from the n→π^*^ transition at this stage), which was much stronger than the NDI^2−^ characteristic absorption between 550 and 675 nm (yellow–red absorption). Therefore, the spectra showed relatively hollow absorption at ∼600–750 nm, which aligned well with their apparent colors. More intuitively, their RGB values at this stage demonstrated overall the lowest G- and B-values among all the electrochromic stages for the 40 MOFs. And, similarly, the colors for MOFs with the –OMe/–F/–OH groups were slightly deeper than those of the others (low L-values), as NKM-906-X-TPDC-Py received more ‘reddish’ spectral contributions from having more TPDC-Py linkers installed (i.e. stronger characteristic absorption).

The electrochromic behaviors of NKM-908-X-TPDC-TDA and NKM-906-X-TPDC-TDA were a step further in complexity (Fig. 4g and h, **31–35** for 908 and **36–40** for 906 series; Fig. S93d). Their colors under natural and NDI^•−^ states were mostly analogous to those installed with TPDC-Py. Interestingly, as the redox events for TPDC-TDA overlapped with the NDI^2−^ event, the color first went clearly purplish/brownish at around −1.6 V, which was due to the conspicuous and intense absorption peak covering 450–620 nm (blue–orange absorption and maximized at 520 nm (green); characteristic for TPDC-TDA; presumably arising from the n→π^*^ transition at this stage). The corresponding H-values were distributed within the magenta–red zone (∼300°–20°), accompanied by clearly dropped L-values at this stage. The colors then went darker as the two characteristic absorption bands for the NDI^2−^ state were enhanced and eventually shifted into dark green at around −2.2 V due to the gradually diminished broad absorption peak. Analogously, NKM-906-X-TPDC-TDA possessed considerably stronger colors than those of NKM-908-X-TPDC-TDA, and especially NKM-906-OH-TPDC-TDA, which showed the darkest in the series (overall very high absorption/low transmittance over the visible-light region). By this stage, the rational installation of various redox-active TPDC-X linkers had offered a reliable strategy in physically introducing as well as controlling the number of ‘secondary’ colors. Together with the possible modification to the ‘primary’ colors contributed from the MOFs themselves, such a multi-domain electrochromic MOF platform is successfully demonstrated as a ‘color palette’ based on light absorption/reflection.

**Figure 5.**
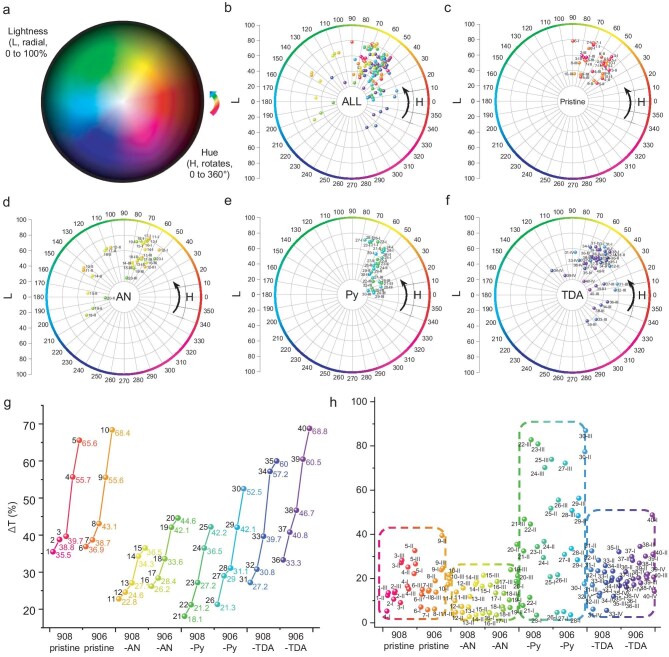
(a) Illustration of the 2D color wheel representing the HSL double-cone model surface (all under full saturation). (b) Distributions of HL-values for all 40 MOFs and correspondingly for (c) NKM-908-R and NKM-906-R, (d) TPDC-AN-installed MOFs, (e) TPDC-Py-installed MOFs and (f) TPDC-TDA-installed MOFs. (g) Grouped Δ*T* line chart showing the evolving trends for Δ*T* within the 40 MOFs. (h) The S-value distribution for all 40 MOFs. (For brevity, MOFs are labeled in Arabic numbers and redox stages are in Roman in the above charts, and their correspondences are listed in Table S5.)

To analytically retrospect the color distribution, as shown in Fig. 5a, the digitized color coordinates are mapped over a more intuitive polar coordinate map based on the color wheel representing the 2D extension of the HSL double-cone model surface (S is set at 100% in this specific wheel; the illustrative model is shown in Fig. S98). Broad coverage across the color gamut has already been demonstrated accordingly with the 40 materials in the current series and is rarely achieved within a set of materials that share almost identical composition and structures (Fig. 5b–f). Here, the reason for showing a denser distribution over the range of 40° < H < 80° is due to their similar colors at neutral. The generally lowered L-values along the electrochromic processes suggested a trend of darkened colors upon higher potentials, which is supported by the positive Δ*T* values for all MOFs (Fig. S95 and Table S5). Also, the evolution of the Δ*T* values evidently manifests that changing R-groups (–H < –Me < –OMe << –F < –OH) and topologies (908 < 906 while X-linkers are installed) are both important factors for deepening the colors (Fig. 5g). The corresponding S-values are plotted in Fig. 5h for reference and varied coverages of spots are illustrated among the four sets of MOFs (TDAs are similar, ANs are narrower and Pys are broader than those of pristine MOFs). Importantly, the subsequent expansion beyond this coverage is entirely possible by further engineering the R-/X-groups (even different types of R-/X-linkers). Most encouragingly, their color types and changing sequences can be designed and utilized a priori based on their individual electrochromic behaviors.

Regarding their stability, all of those thin films were fine adhered after >10 cycles of experiments by using two-potential-step chronoamperometry (0 and −2.2 V) *in situ*, with essentially consistent surface morphology and crystallinity presented in the SEM images and PXRD patterns compared to those before the circulations (Figs S8–S12, S14–S18 and S20–S49). Meanwhile, little changes in the Δ*T* values and current densities of these MOF thin films were demonstrated (Figs S99–S108 and Table S6) and their switching time between states (coloring time (*t*_c_) and bleaching time (*t*_b_)) were obtained accordingly. The coloring efficiencies (CEs) of these MOFs were calculated according to the following equation: CE(*λ*) = ΔOD/*Q*, where ΔOD = log[*T*_b_/*T*_c_] (*T*_b_ and *T*_c_ represent the transmittance at bleached and colored states. respectively), OD represents the optical density and Q represents the injected/ejected charge density (C cm^−2^). Table S6 also summarizes the Δ*T, t*_c_, *t*_b_ and CE values in comparison to other MOFs in the literature. Meanwhile, reaching the full redox events of these MOFs required potentials lower than −2.2 V (mostly below −1.5 V; some standard operation voltages for routine usages are listed as follows: dry cell (1.5 V), single lead-acid cell (2.0 V), lithium battery (>3 V)). Considering the overall robustness, convenient set-ups and low energy consumption of these materials, such a MOF-based ‘color palette’ allows tailored electrochromic properties toward vast applicable scenarios, especially for customized simple displayers and anti-counterfeiting. For instance, this platform can serve as built-in modules within appropriate electronics and can be frequently replaced/upgraded over different batches, which will be tough for fakers to predict/replicate.

## CONCLUSION

In summary, we present the first MOF-based platform capable as a potential solid-state electrochromic ‘color palette’. Altogether, 40 different Zr-NDI-based MOFs were successfully prepared as durable thin films based on the combination of five/three types of R-/X-linkers. This exhibition demonstrated a systematic modification of the MOF structures following a general synthetic protocol, as well as the fine-tuning of their overall electrochromic behaviors. While the same NDI core was maintained, modifying the R-group on the ‘modifiable peripheral zones’ at the edges of these NDI-containing R-linkers brought color intensification to the NKM-908-R/NKM-906-R during the redox processes (referring to the variation and intensification of the primary ‘color’). Meanwhile, alternation between the **csq**/**scu** topologies did not change the color directly, but different quantities of X-linkers installed in NKM-908-R-TPDC-X/NKM-906-R-TPDC-X did, further offering noticeable intensification of the diverse ‘colors’ that originated from the different X-linkers (referring to the variation and intensification of the secondary ‘colors’). The spectroscopic mixtures of two ‘colors’ that resulted from such multidirectional modifications therefore demonstrated broad color differentiation and their predictable changing/mixing across these MOFs, accompanied by good electrochromic stability and cyclability of the fabricated MOF thin films. Given the considerable potential in further extension from a greater choice of R-/X-groups, this work offers a new and universal solution towards the development of customized electrochromic MOF materials for practical use.

## METHODS

The detailed preparation and characteristic methods of the materials are available as Supplementary Data.

## Supplementary Material

nwaf326_Supplemental_Files

## Data Availability

CCDC 2445635, 2445637, 2445656 and 2445658 contain the supplementary crystallographic data for this paper. These data can be obtained free of charge via https://www.ccdc.cam.ac.uk/structures/, by emailing data_request@ccdc.cam.ac.uk or by contacting The Cambridge Crystallographic Data Centre, 12 Union Road, Cambridge CB2 1EZ, UK; fax: +44 1223 336 033.

## References

[1] Yang G, Zhang YM, Cai Y et al. Advances in nanomaterials for electrochromic devices. Chem Soc Rev 2020; 49: 8687–720. 10.1039/D0CS00317D 33078186

[2] Rai V, Singh RS, Blackwood DJ et al. A review on recent advances in electrochromic devices: a material approach. Adv Eng Mater 2020; 22: 2000082. 10.1002/adem.202000082

[3] Gu C, Jia AB, Zhang YM et al. Emerging electrochromic materials and devices for future displays. Chem Rev 2022; 122: 14679–721. 10.1021/acs.chemrev.1c01055 35980039 PMC9523732

[4] Yang P, Sun P, Chai Z et al. Large-scale fabrication of pseudocapacitive glass windows that combine electrochromism and energy storage. Angew Chem Int Ed 2014; 53: 11935–9. 10.1002/anie.201407365 25212514

[5] Wang Y, Wang S, Wang X et al. A multicolour bistable electronic shelf label based on intramolecular proton-coupled electron transfer. Nat Mater 2019; 18: 1335–42. 10.1038/s41563-019-0471-8 31501553

[6] Sui C, Pu J, Chen T-H et al. Dynamic electrochromism for all-season radiative thermoregulation. Nat Sustain 2023; 6: 428–37. 10.1038/s41893-022-01023-2

[7] Davy NC, Sezen-Edmonds M, Gao J et al. Pairing of near-ultraviolet solar cells with electrochromic windows for smart management of the solar spectrum. Nat Energy 2017; 2: 17104. 10.1038/nenergy.2017.104

[8] Cai G, Zhu R, Liu S et al. Tunable intracrystal cavity in tungsten bronze-like bimetallic oxides for electrochromic energy storage. Adv Energy Mater 2022; 12: 2103106. 10.1002/aenm.202103106

[9] Li B, Dang J, Zhuang Q et al. Recent advances in inorganic electrochromic materials from synthesis to applications: critical review on functional chemistry and structure engineering. Chem Asian J 2022; 17: e202200022. 10.1002/asia.202200022 35191172

[10] Islam SM, Hernandez TS, McGehee MD et al. Hybrid dynamic windows using reversible metal electrodeposition and ion insertion. Nat Energy 2019; 4: 223–9. 10.1038/s41560-019-0332-3

[11] Wang Z, Wang X, Cong S et al. Towards full-colour tunability of inorganic electrochromic devices using ultracompact fabry-perot nanocavities. Nat Commun 2020; 11: 302. 10.1038/s41467-019-14194-y 31949150 PMC6965179

[12] Beaujuge PM, Ellinger S, Reynolds JR. The donor–acceptor approach allows a black-to-transmissive switching polymeric electrochrome. Nat Mater 2008; 7: 795–9. 10.1038/nmat2272 18758455

[13] Bulloch RH, Kerszulis JA, Dyer AL et al. An electrochromic painter's palette: color mixing via solution co-processing. ACS Appl Mater Interfaces 2015; 7: 1406–12. 10.1021/am507514z 25580827

[14] Kim J, Rémond M, Kim D et al. Electrochromic conjugated polymers for multifunctional smart windows with integrative functionalities. Adv Mater Technol 2020; 5: 1900890. 10.1002/admt.201900890

[15] Wang X, Chen K, de Vasconcelos LS et al. Mechanical breathing in organic electrochromics. Nat Commun 2020; 11: 211. 10.1038/s41467-019-14047-8 31924784 PMC6954196

[16] Shimoyama D, Baser-Kirazli N, Lalancette RA et al. Electrochromic polycationic organoboronium macrocycles with multiple redox states. Angew Chem Int Ed 2021; 60: 17942–6. 10.1002/anie.202105852 34111328

[17] Jamdegni M, Kaur A. Review—polymeric/small organic molecules-based electrochromic devices: how far toward realization. J Electrochem Soc 2022; 169: 030541. 10.1149/1945-7111/ac5c04

[18] Shao Z, Huang A, Ming C et al. All-solid-state proton-based tandem structures for fast-switching electrochromic devices. Nat Electron 2022; 5: 45–52. 10.1038/s41928-021-00697-4

[19] Gu C, Wang S, He J et al. High-durability organic electrochromic devices based on in-situ-photocurable electrochromic materials. Chem 2023; 9: 2841–54. 10.1016/j.chempr.2023.05.015

[20] Wang B, Zhang W, Zhao F et al. An overview of recent progress in the development of flexible electrochromic devices. Nano Mater Sci 2023; 5: 369–91. 10.1016/j.nanoms.2022.08.002

[21] Zhou HC, Long JR, Yaghi OM. Introduction to metal–organic frameworks. Chem Rev 2012; 112: 673–4. 10.1021/cr300014x 22280456

[22] Furukawa H, Cordova KE, O'Keeffe M et al. The chemistry and applications of metal-organic frameworks. Science 2013; 341: 1230444. 10.1126/science.1230444 23990564

[23] Behera N, Duan J, Jin W et al. The chemistry and applications of flexible porous coordination polymers. EnergyChem 2021; 3: 100067. 10.1016/j.enchem.2021.100067

[24] Jiao L, Seow JYR, Skinner WS et al. Metal–organic frameworks: structures and functional applications. Mater Today 2019; 27: 43–68. 10.1016/j.mattod.2018.10.038

[25] Chen Z, Hanna SL, Redfern LR et al. Reticular chemistry in the rational synthesis of functional zirconium cluster-based MOFs. Coord Chem Rev 2019; 386: 32–49. 10.1016/j.ccr.2019.01.017

[26] Lang F, Pang J, Bu X-H. Stimuli-responsive coordination polymers toward next-generation smart materials and devices. eScience 2024; 4: 100231. 10.1016/j.esci.2024.100231

[27] Xie LS, Skorupskii G, Dinca M. Electrically conductive metal-organic frameworks. Chem Rev 2020; 120: 8536–80. 10.1021/acs.chemrev.9b00766 32275412 PMC7453401

[28] Tao CA, Li Y, Wang J. The progress of electrochromic materials based on metal–organic frameworks. Coord Chem Rev 2023; 475: 214891. 10.1016/j.ccr.2022.214891

[29] Fan X, Pan M, Li X et al. Research progress of MOF electrochromic materials. Res Chem Mater 2024; 3: 230–45. 10.1016/j.recm.2024.03.001

[30] Li J, Ott S. The molecular nature of redox-conductive metal-organic frameworks. Acc Chem Res 2024; 57: 2836–46. 10.1021/acs.accounts.4c00430 39288193 PMC11447836

[31] Suraru SL, Wurthner F. Strategies for the synthesis of functional naphthalene diimides. Angew Chem Int Ed 2014; 53: 7428–48. 10.1002/anie.201309746 24961807

[32] Li R, Li K, Wang G et al. Ion-transport design for high-performance Na+-based electrochromics. ACS Nano 2018; 12: 3759–68. 10.1021/acsnano.8b00974 29595953

[33] Lu Z, Li R, Ping L et al. Ultra-stable ionic-liquid-based electrochromism enabled by metal-organic frameworks. Cell Rep Phy Sci 2022; 3: 100866. 10.1016/j.xcrp.2022.100866

[34] Fan X, Wang S, Pan M et al. Biphenyl dicarboxylic-based Ni-IRMOF-74 film for fast-switching and high-stability electrochromism. ACS Energy Lett 2024; 9: 2840–7. 10.1021/acsenergylett.4c00492

[35] Freund R, Zaremba O, Arnauts G et al. The current status of MOF and cof applications. Angew Chem Int Ed 2021; 60: 23975–4001. 10.1002/anie.202106259 33989445

[36] Jang W, Yoo H, Shin D et al. Colorimetric identification of colorless acid vapors using a metal-organic framework-based sensor. Nat Commun 2025; 16: 385. 10.1038/s41467-024-55774-x 39755687 PMC11700211

[37] Mallakpour S, Nikkhoo E, Hussain CM. Application of MOF materials as drug delivery systems for cancer therapy and dermal treatment. Coord Chem Rev 2022; 451: 214262. 10.1016/j.ccr.2021.214262

[38] Li C, Zhang H, Liu M et al. Recent progress in metal–organic frameworks (MOFs) for electrocatalysis. Ind Chem Mater 2023; 1: 9–38. 10.1039/D2IM00063F

[39] AlKaabi K, Wade CR, Dincă M. Transparent-to-dark electrochromic behavior in naphthalene-diimide-based mesoporous MOF-74 analogs. Chem 2016; 1: 264–72. 10.1016/j.chempr.2016.06.013

[40] Johnson BA, Bhunia A, Fei H et al. Development of a UiO-type thin film electrocatalysis platform with redox-active linkers. J Am Chem Soc 2018; 140: 2985–94. 10.1021/jacs.7b13077 29421875 PMC6067658

[41] Mallick A, Liang H, Shekhah O et al. Made-to-order porous electrodes for supercapacitors: mOFs embedded with redox-active centers as a case study. Chem Commun 2020; 56: 1883–6. 10.1039/C9CC08860A 31951225

[42] Li C, Zhang H, Lang F et al. Efficiently regulating the electrochromic behavior of naphthalene-diimide-based zirconium-organic frameworks through linker installation. Nat Commun 2025; 16: 1405. 10.1038/s41467-024-55473-7 39915474 PMC11803088

[43] Bai Y, Dou Y, Xie LH et al. Zr-based metal-organic frameworks: design, synthesis, structure, and applications. Chem Soc Rev 2016; 45: 2327–67. 10.1039/C5CS00837A 26886869

[44] Yuan S, Huang L, Huang Z et al. Continuous variation of lattice dimensions and pore sizes in metal-organic frameworks. J Am Chem Soc 2020; 142: 4732–8. 10.1021/jacs.9b13072 32058715

[45] Shupletsov L, Topal S, Schieck A et al. Linker conformation controls oxidation potentials and electrochromism in highly stable Zr-based metal-organic frameworks. J Am Chem Soc 2024; 146: 25477–89. 10.1021/jacs.4c04653 39226465

[46] Lang F, Zhang L, Li Y et al. Retrieving the stability and practical performance of activation-unstable mesoporous Zr(IV)-MOF for highly efficient self-calibrating acidity sensing. Angew Chem Int Ed 2025; 64: e202422517. 10.1002/anie.202422517 39810598

[47] Feng L, Pang J, She P et al. Metal-organic frameworks based on group 3 and 4 metals. Adv Mater 2020; 32: e2004414. 10.1002/adma.202004414 32902012

[48] Kung CW, Goswami S, Hod I et al. Charge transport in zirconium-based metal-organic frameworks. Acc Chem Res 2020; 53: 1187–95. 10.1021/acs.accounts.0c00106 32401008

[49] Wade CR, Li M, Dinca M. Facile deposition of multicolored electrochromic metal-organic framework thin films. Angew Chem Int Ed 2013; 52: 13377–81. 10.1002/anie.201306162 24133021

[50] Kumar A, Li J, Inge AK et al. Electrochromism in isoreticular metal-organic framework thin films with record high coloration efficiency. ACS Nano 2023; 17: 21595–603. 10.1021/acsnano.3c06621 37851935 PMC10655172

[51] Li J, Kumar A, Ott S. Diffusional electron transport coupled to thermodynamically driven electron transfers in redox-conductive multivariate metal-organic frameworks. J Am Chem Soc 2024; 146: 12000–10. 10.1021/jacs.4c01401 38639553 PMC11066865

[52] Yuan S, Lu W, Chen YP. Sequential linker installation: precise placement of functional groups in multivariate metal-organic frameworks. J Am Chem Soc 2015; 137: 3177–80. 10.1021/ja512762r 25714137

[53] Pang J, Yuan S, Qin J et al. Enhancing pore-environment complexity using a trapezoidal linker: toward stepwise assembly of multivariate quinary metal-organic frameworks. J Am Chem Soc 2018; 140: 12328–32. 10.1021/jacs.8b07411 30227706

[54] Pang J, Yuan S, Du D et al. Flexible zirconium MOFs as bromine-nanocontainers for bromination reactions under ambient conditions. Angew Chem Int Ed 2017; 56: 14622–6. 10.1002/anie.201709186 28990352

[55] Cheng HD, Jiang XH, Sun Y et al. Color image segmentation: advances and prospects. Pattern Recognit 2001; 34: 2259–81. 10.1016/S0031-3203(00)00149-7

[56] Mazur F, Han Z, Tjandra AD et al. Digitalization of colorimetric sensor technologies for food safety. Adv Mater 2024; 36: e2404274. 10.1002/adma.202404274 38932639

