# Supplementary material for: Multidirectional color palette of electrochromic metal–organic frameworks
Source: Natl Sci Rev. 2025 Aug 12;12(10):nwaf326. doi: 10.1093/nsr/nwaf326 (PMC12475564; doi:10.1093/nsr/nwaf326)
Supplement: nwaf326_Supplemental_Files [file nwaf326_supplemental_files.zip › 908-Supplementary_Information.pdf]

Multi-Directional Color Palette of Electrochromic Metal-Organic Frameworks

Cha Li[1], Jinli Zhang[1], Yudong Lian[1], Kai Zhang[1], Hao Zhang[1], Lin Xu[1], Yanghe Liu[1], Feifan Lang[1,*], Jiandong Pang[1,3,*], Xian-He Bu[1,2,3]

[1] School of Materials Science and Engineering, Smart Sensing Interdisciplinary Science Center, Collaborative Innovation Center of Chemical Science and Engineering, Nankai University, Tianjin 300350, P. R. China.

[2] State Key Laboratory of Elemento-Organic Chemistry, Frontiers Science Center for New Organic Matter, College of Chemistry, Nankai University, Tianjin 300071, P. R. China.

[3] Haihe Laboratory of Sustainable Chemical Transformations, Tianjin 300192, P. R. China.

E-mail addresses of corresponding authors: fflang@nankai.edu.cn; jdpang@nankai.edu.cn.

Table of Contents

Table of Contents 2
Supplementary Methods 4
Single-crystal X-ray diffraction (SCXRD) 4
Three-dimensional micro electron diffraction crystallography 4
Powder X-ray diffraction (PXRD) 5
^{1}H Nuclear Magnetic Resonance (NMR) spectroscopy 5
Mass Spectrometry (MS) 5
Elemental analyses (EA) 5
Scanning Electron Microscopy (SEM) 5
Volumetric N_2 adsorption analyses 6
Electrochromic tests (including CV curves and UV-vis spectra) 6
Chemicals 6
Syntheses of H_4NDTB-R 8
(1) Synthesis of H_4NDTB-H 8
(2) Synthesis of H_4NDTB-Me 10
(3) Synthesis of H_4NDTB-OMe 12
(4) Synthesis of H_4NDTB-F 13
(5) Synthesis of H_4NDTB-OH 16
Syntheses of H_2TPDC-X 18
Syntheses of NKM-908-R crystals 20
Syntheses of NKM-908-R-TPDC-X crystals 20
Syntheses of NKM-906-R crystals 20
Syntheses of NKM-906-R-TPDC-X crystals 20
Fabrication of MOF thin films 21
Supplementary Figures and Tables 24
Table S1: SCXRD data collection and structure solutions 24
Table S2. Elemental analyses results of all forty MOFs. 25
Figure S8–S12: PXRD patterns and SEM images for NKM-908-R 26
Figure S13 & Table S2: Volumetric gas adsorption for NKM-908-R series and NKM-908-R-TPDC-X series 28

Figure S14–S18: PXRD patterns and SEM images for NKM-906-R 30

Figure S19 & Table S3: Volumetric gas adsorption for NKM-906-R series and NKM-906-R-TPDC-X series 33

Figure S20–S34: PXRD patterns and SEM images for NKM-908-R-TPDC-X.................. 34

Figure S35–S49: PXRD patterns and SEM images for NKM-906-R-TPDC-X.................. 42

Figure S50: Optical photographs for all forty as-synthesized MOF thin films (dry) on ITO glass. 49

Figure S51–S60: ^{1}H NMR spectra of digested NKM-908-R and NKM-906-R. 50

Figure S61–S75: ^{1}H NMR spectra of digested NKM-908-R-TPDC-X.............................. 55

Figure S76–S90: ^{1}H NMR spectra of digested NKM-906-R-TPDC-X.............................. 62

Figure S91: The acquired reference colors for all forty MOF thin films. 70

Table S4: Summary of reference labels and representative potentials of all forty MOF thin films during electrochromic process, and the corresponding RGB/HSL coordinates of the colors. 71

Figure S92: CV-scan curves of all forty MOF thin films over one electrochromic process. 74

Figure S93: Proposed redox mechanisms for all linkers. 75

Figure S94: CV-scan curves of five pristine H_4NDTB-R 75

Figure S95: Optical transmittance spectra and corresponding optical contrast (ΔT) values of all forty MOF thin films over one electrochromic process. 76

Figure S96: CV-scan curves of five pristine H_2TPDC-X 77

Figure S97: Photographs of the drop-cast film fabricated from using ink of organic linkers 77

Figure S98: Illustration of HSL model and 2D color wheel... 77

Figure S99–S106: Dynamic optical transmittance spectra and coloring time for all forty MOF thin films over 10 cycles. 78

Figure S107: Changes of current density for all forty MOF thin films over 10 cycles. 81

Table S6: Summary of the MOF-based electrochromic materials in the literature with compassion to the forty MOFs in this work. 83

References 85

Supplementary Methods

Single-crystal X-ray diffraction (SCXRD)

Single crystal of NKM-908-Me, NKM-908-OH and NKM-906-OMe were, respectively, harvested from the mother liquid and transferred into oil without further treatment, and mounted onto a loop for single-crystal X-ray diffraction. The experiments were carried on a Bruker Smart Apex diffractometer equipped with a Cu-K_{α} sealed-tube X-ray source ($\lambda = 1.54178$ Å, graphite monochromated) under 100 K by using an ω scan mode. The data frames were recorded using the program *APEX3* and processed using the program *SAINT* routine within *APEX3*.[1] The data were corrected for absorption and beam corrections based on the multi-scan technique as implemented in *SADABS*.[2] The crystal structures were solved by Direct method using *SHELXS* and refined by full-matrix least-squares on F^2 using *SHELXL* within the *Olex2* graphical user interface.[3,4] All non-hydrogen atoms were refined with anisotropic displacement parameters. Hydrogen atoms were positioned at geometrically calculated positions and refined by riding models. The diffused electron densities resulting from these highly disordered solvent molecules were removed using the *SQUEEZE* routine of *PLATON*, as attempts to locate and refine them with reasonable positions/structures were unsuccessful.[5]

Three-dimensional micro electron diffraction crystallography

Electron diffraction data of NKM-906-H-TPDC-4F were collected at room temperature using a Rigaku XtaLAB Synergy-ED diffractometer, equipped with a JEOL electron source up to 200 kV (λ = 0.0251 Å) and a HyPix-ED hybrid pixel array detector. The MOF microcrystals were dispersed in ethanol and sonicated for 5 min. Then the suspension was dropped onto a copper grid, which was installed as the test sample after completely dried the surface of the grid. The diffraction data were processed using *CrysAlis^{Pro}* software.[6] All structures were solved via Intrinsic Phasing method using *SHELXT* and refined with *SHELXL* within the *Olex2* graphical user interface.

Powder X-ray diffraction (PXRD)

PXRD analyses were performed on a Bruker D8-Focus Bragg-Brentano X-ray powder diffractometer equipped with a copper-sealed tube ($\lambda = 1.54178$ Å) at 40 kV and 40 mA. Due to the experimental condition, some of the PXRD patterns for the films were collected after scratched them off from the ITO glass. The calculated PXRD patterns were produced using the *Mercury* software based on the corresponding single-crystal structures.[7]

^{1}H Nuclear Magnetic Resonance (NMR) spectroscopy

For intermediate compounds and ligands, around 5 mg of the sample was dissolved in 0.5 mL DMSO-d_6 or $CDCl_3$ for ^{1}H NMR measurement.

For analyzing the molar ratio of the linkers (n_R:n_X) within the MOF thin films, the MOF microcrystals were gently removed from the ITO electrode. About 5 mg of the sample was first digested with one drop of D_2SO_4-d_2. About 0.5 mL DMSO-d_6 was then added to the mixture and sonicated for 5 min. The upper clear solution was then collected for ^{1}H NMR measurement. Multiplicities are recorded as: s = singlet, d = doublet, t = triplet, dd = doublet of doublets, br = broad singlet and m = multiplet.

Mass Spectrometry (MS)

High resolution mass spectrometry was performed using Agilent 6545 liquid chromatography quadrupole time of flight (LC/Q-TOF) mass spectrometer.

Elemental analyses (EA)

Elemental analyses (C, H, and N) were performed for all forty MOFs using the Elementar vario EL cube.

Scanning Electron Microscopy (SEM)

The SEM images were examined on the FEI Nova Nano 230 scanning electron microscope. The energy of the electron beam was 15 and 20 keV, respectively.

Volumetric N_2 adsorption analyses

Volumetric N_2 adsorption analyses were performed on a Micromeritics ASAP 2020 surface area and pore size analyzer. Typically, the as-synthesized MOF crystals were thoroughly washed with DMF and soaked in acetone to exchange the lattice DMF molecules into acetone. The solvent-exchanged MOF crystals were filtered out and activated at 80 °C under vacuum for 10 h. After degassed the sample, a liquid nitrogen bath was used to maintain the 77 K environment during volumetric N_2 sorption analyses. The specific surface areas and pore size distributions of the MOF samples were determined according to the N_2 adsorption isotherms using the Brunauer–Emmett–Teller (BET) model and Nonlocal Density Functional Theory (NLDFT) model, respectively.

Electrochromic tests (including CV curves and UV-vis spectra)

Cyclic voltammetry (CV) curves were recorded by a CHI760E potentiostat. All measurements were performed using a standard three-electrode system. The 0.1 M $[(^{n}Bu)_4N]PF_6$/DMF electrolyte was purged with nitrogen for 30 min before used in the measurements. In a typical measurement, the working electrode is one of the MOF thin film ITO electrodes described in this work, the reference electrode is Ag/AgCl, and the platinum mesh electrode is the counter electrode. The CV test of the ligand was performed using a glassy carbon electrode (electrode area: 0.07068 cm^2), with a solution of 1 mM H_4NDTB-R or H_2TPDC-X containing 0.1 M $[(^{n}Bu)_4N]PF_6$ in DMF.

UV-vis absorbance spectra of the thin film electrodes were performed on a KU-T6PC UV spectrophotometer (Nanjing KENFAN Electronic Technology Co. Ltd.) using a 10 mm path length quartz cuvette, with platinum wire as the counter electrode, Ag/AgCl as the reference electrode, and 0.1 M $[(^{n}Bu)_4N]PF_6$/DMF as the electrolyte.

Chemicals

All the chemicals were purchased from commercial suppliers and used without further purification. The 4-nitrophenol (99%), KBr (99.99% metals basis), N-Bromosuccinimide (NBS) (AR), 4-methylbenzenesulfonic acid (≥98%), CH_3I (99%), K_2CO_3 (98%), Fe (≥99%), NH_4Cl (99%), Cs_2CO_3 (99% metals basis), $Pd(PPh_3)_4$ (99%), NaOH (97%), 1,4,5,8-Naphthalenetetracarboxylic dianhydride (96%), 4-Ethoxycarbonylphenylboronic acid (97%), benzoic acid (99%), $ZrCl_4$ (98%)

were purchased from Aladdin Industrial Inc (Shanghai, China). The Tetra-n-butylammonium hexafluorophosphate (99%+), p-Dibromobenzene (99%), 1,2,4,5-Tetramethylbenzene Durol (98%), 9,10-Dibromoanthracene (98%), 4,7-Dibromo-2,1,3-BenZzothiadiazole (98%), 2,5-Dibromopyridine(98%), 1,4-Dibromo-2,5-dimethoxybenzene (98%), N, N-Dimethylformamide (DMF) (≥99%), Ethanol (EtOH) (AR), 1,4-Dioxane (≥99.9%(GC)), tetrahydrofuran (THF) (AR) were purchased from Shanghai Titan Scientific Co., Ltd (Shanghai, China).

Syntheses of H_4NDTB-R

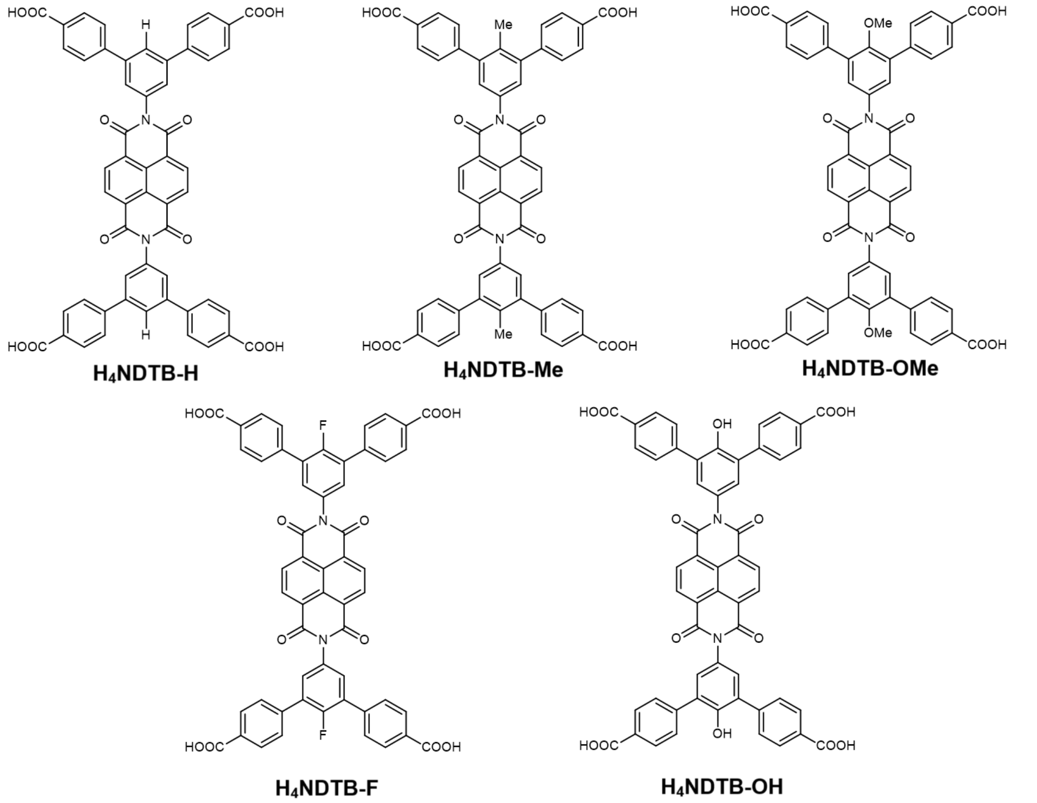

Figure S1. List of H_4NDTB-R (R-linkers, R = H, Me, OMe, F, OH).

(1) Synthesis of H_4NDTB-H

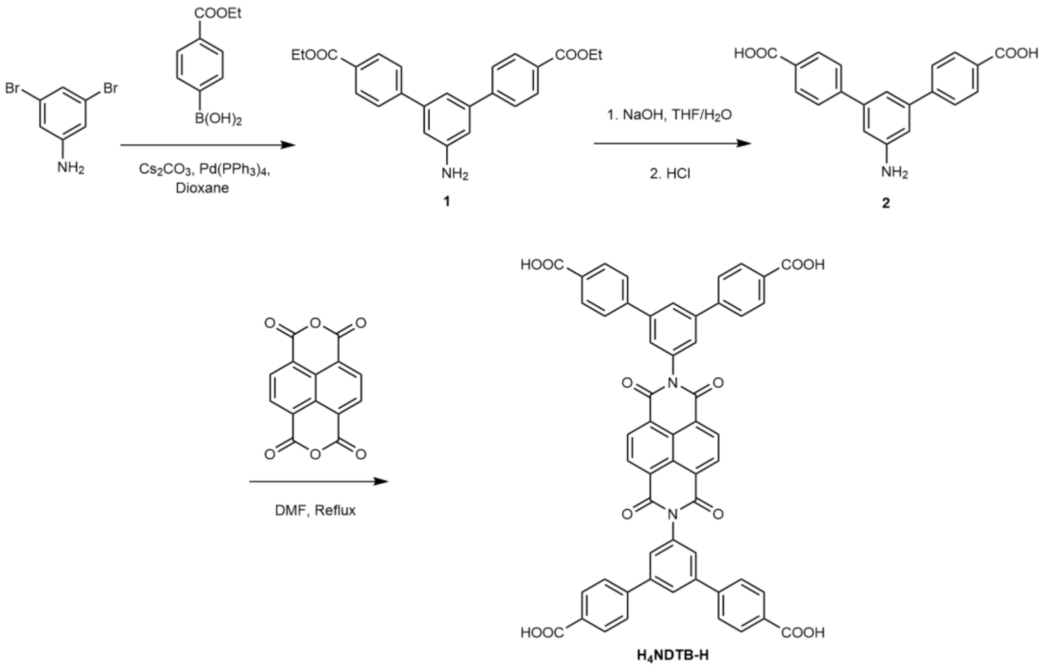

Figure S2. Synthesis of H_4NDTB-H.

Synthesis of diethyl 5'-amino-[1,1':3',1''-terphenyl]-4,4''-dicarboxylate **(1)**. 3,5-dibromoaniline (5 g, 20 mmol), (4-(ethoxycarbonyl)phenyl)boronic acid (11.6 g, 60 mmol), Cs_2CO_3 (26 g, 80 mmol), and tetrakis(triphenylphosphine)palladium (1.156 g, 1 mmol) were added to a 500 mL Schlenk flask charged with a stir bar. The flask was pumped under vacuum and refilled with N_2 for three times, followed by transferring 200 mL of degassed 1,4-dioxane into the system. The reaction mixture was heated to 85 ℃ and stirred for 72 h under a N_2 atmosphere. After cooled to room temperature, the organic solvent was removed using a rotary evaporator, and the resulting mixture was transferred into water and extracted with dichloromethane (3 × 50 mL). The combined organic layers were dried over anhydrous $MgSO_4$, and the solvent was removed with rotary evaporator again. After purification by column chromatography on silica gel using ethyl acetate/hexane (1:4 v/v) as eluent, compound **2** was obtained as a pale yellow solid from evaporating the fraction (4.97 g, yield: 64 %). ^{1}H NMR (400 MHz, $CDCl_3$): δ = 1.40 (t, 6H), 4.41 (q, 4H), 6.95 (s, 2H), 7.23 (s, 1H), 7.66 (d, 4H), 8.10 (d, 4H) ppm.

Synthesis of 5'-amino-[1,1':3',1''-terphenyl]-4,4''-dicarboxylic acid **(2)**. Compound **1** (0.54 g, 1.4 mmol) was dissolved in 20 mL of THF, to which 30 mL of 10 M NaOH aqueous solution was added. The mixture was stirred under reflux for 10 h, and then the organic solvent was removed using a rotary evaporator. The aqueous phase was acidified to pH = 2 using 6 M HCl aqueous solution. The resulting precipitate was collected *via* filtration, washed with water (200 mL), and dried under vacuum to afford compound **2** (0.433 g, 93 %). ^{1}H NMR (400 MHz, $CDCl_3$): δ = 7.13 (s, 2H), 7.35 (s, 1H), 7.80 (d, 4H), 8.04 (d, 4H), 13.01 (br, 2H) ppm.

Synthesis of 5',5''''-(1,3,6,8-tetraoxo-1,3,6,8-tetrahydrobenzo[lmn][3,8]phenanthroline-2,7-diyl)bis([1,1':3',1''-terphenyl]-4,4''-dicarboxylic acid) **(H_4NDTB-H)**. Compound **2** (3.33 g, 10 mmol) and 1,4,5,8-tetracarboxydianhydride (1.34 g, 5.0 mmol) were added to a 250 mL Schlenk flask. The flask was pumped under vacuum and refilled with N_2 three times, followed by transferring 50 mL of degassed N,N-Dimethylformamide (DMF) into the system. The reaction mixture was heated to 160 ℃ and stirred for 12 h under a N_2 atmosphere. After cooled to room temperature, 5 mL of 1 mol/L HCl was added to the mixture, which was then poured into 500 mL

of water. The product was collected by filtration, washed with water, ethanol and acetone, and dried under vacuum to give a light yellow solid of **H_4NDTB-H** (3.63 g, yield: 81.0%).^{1}H NMR (400 MHz, DMSO-d_6): δ = 7.88 (d, 8H), 7.95 (s, 4H), 8.02 (d, 8H), 8.10 (s, 2H), 8.67 (s, 4H) ppm. HRMS (m/z): [M]+ calculated. for $C_{54}H_{30}N_2O_{12}$: 898.18, found: 898.1818.

(2) Synthesis of H_4NDTB-Me

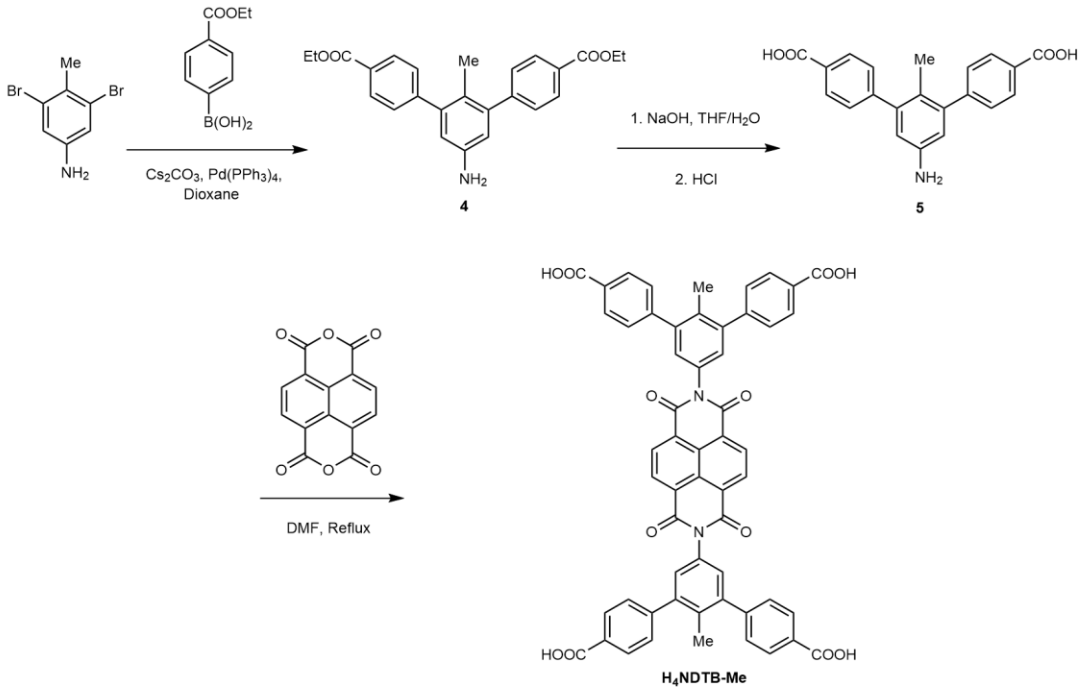

Figure S3. Synthesis of H_4NDTB-Me.

Synthesis of diethyl 5'-amino-2'-methyl-[1,1':3',1''-terphenyl]-4,4''-dicarboxylate **(4)**. 3,5-dibromo-4-methylaniline (5.28 g, 20 mmol), (4-(ethoxycarbonyl)phenyl)boronic acid (11.6 g, 60 mmol), Cs_2CO_3 (11 g, 80 mmol), and tetrakis(triphenylphosphine)palladium (1.156 g, 1 mmol) were added to a 500 mL Schlenk flask charged with a stir bar. The flask was pumped under vacuum and refilled with N_2 for three times, followed by transferring 200 mL of degassed 1,4-dioxane into the system. The reaction mixture was heated to 85 ℃ and stirred for 72 h under a N_2 atmosphere. After cooled to room temperature, the organic solvent was removed using a rotary evaporator, and the resulting mixture was transferred into water and extracted with dichloromethane (3 × 50 mL). The combined organic layers were dried over anhydrous $MgSO_4$, and the solvent was removed

with rotary evaporator again. After purification by column chromatography on silica gel using ethyl acetate/hexane (1:4 v/v) as eluent, compound **5** was obtained as a pale yellow solid from evaporating the fraction (5.6 g, yield: 70 %). ^{1}H NMR (400 MHz, $CDCl_3$): δ = 1.42 (t, 6H), 1.95 (s, 3H), 4.42 (q, 4H), 6.65 (s, 2H), 7.43 (d, 4H), 8.10 (d, 4H) ppm.

Synthesis of 5'-amino-2'-methyl-[1,1':3',1''-terphenyl]-4,4''-dicarboxylic acid **(5)**. Compound **4** (0.58 g, 1.4 mmol) was dissolved in 20 mL of THF, to which 30 mL of 10 M NaOH aqueous solution was added. The mixture was stirred under reflux for 10 h, and then the organic solvent was removed using a rotary evaporator. The aqueous phase was acidified to pH = 2 using 6 M HCl aqueous solution. The resulting precipitate was collected *via* filtration, washed with water (200 mL), and dried under vacuum to afford compound **5** (0.44 g, 91 %). ^{1}H NMR (400 MHz, $CDCl_3$): δ = 2.00 (s, 3H), 3.45 (s, 2H), 7.06 (s, 2H), 7.51 (d, 4H), 8.04 (d, 4H), 12.99 (br, 2H) ppm.

Synthesis of 5',5''''-(1,3,6,8-tetraoxo-1,3,6,8-tetrahydrobenzo[lmn][3,8]phenanthroline-2,7-diyl)bis(2'-methyl-[1,1':3',1''-terphenyl]-4,4''-dicarboxylic acid) **(H_4NDTB-Me)**. Compound **5** (3.47 g, 10 mmol) and 1,4,5,8-tetracarboxydianhydride (1.34 g, 5.0 mmol) were added to a 250 mL Schlenk flask. The flask was pumped under vacuum and refilled with N_2 three times, followed by transferring 50 mL of degassed N,N-Dimethylformamide (DMF) into the system. The reaction mixture was heated to 160 ℃ and stirred for 12 h under a N_2 atmosphere. After cooled to room temperature, 5 mL of 1 mol/L HCl was added to the mixture, which was then poured into 500 mL of water. The product was collected by filtration, washed with water, ethanol and acetone, and dried under vacuum to give a light yellow solid of **H_4NDTB-Me** (3.65 g, yield: 79%). ^{1}H NMR (400 MHz, DMSO-d_6): δ = 2.20 (s, 6H), 7.44 (s, 4H), 7.60 (d, 8H), 8.03 (d, 8H), 8.69 (s, 4H), 13.05 (br, 4H) ppm. HRMS (m/z): [M]+ calculated. for $C_{56}H_{34}N_2O_{12}$: 926.21, found: 957.2015.

(3) Synthesis of H_4NDTB-OMe

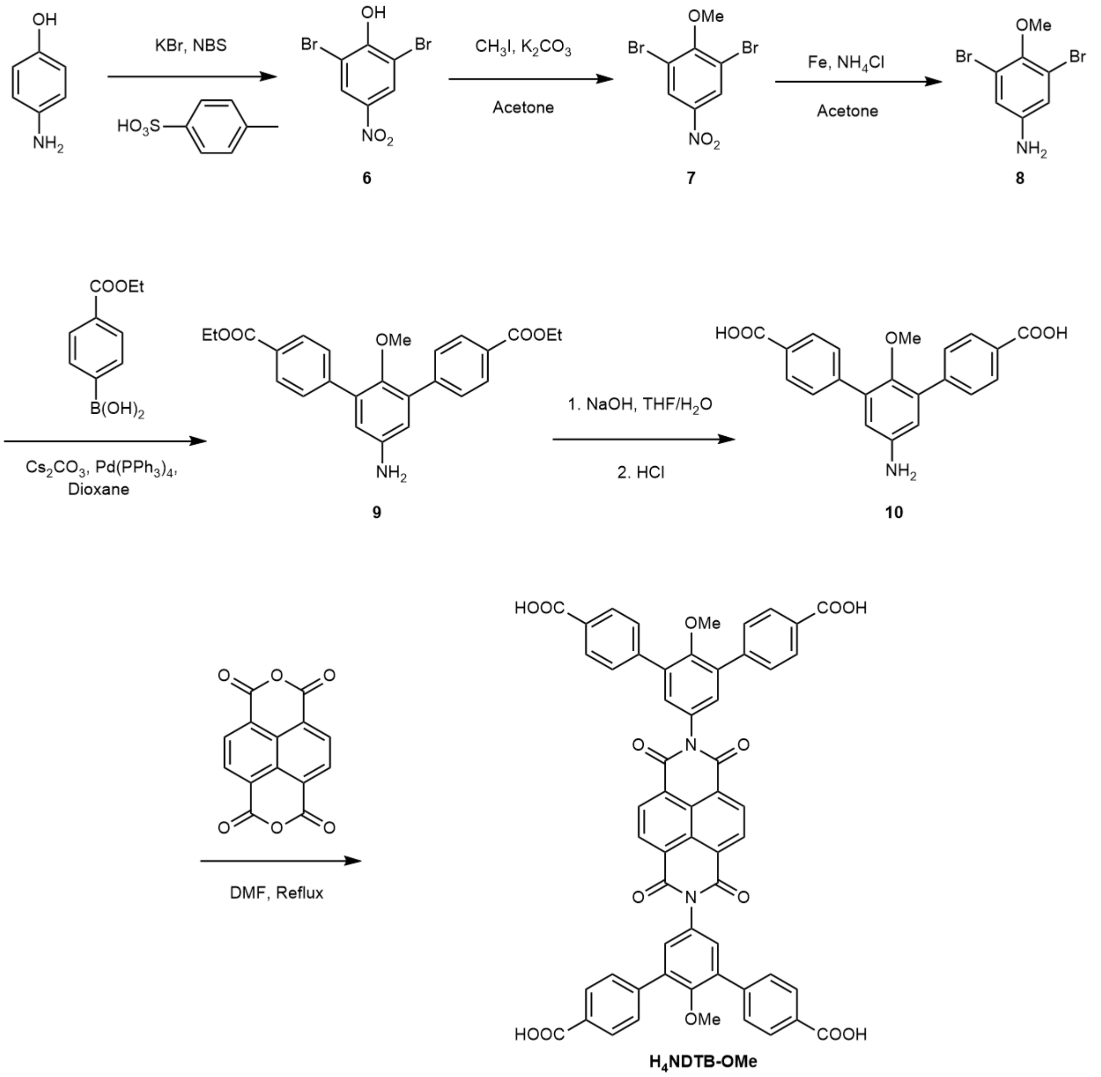

Figure S4. Synthesis of H_4NDTB-OMe.

The synthesis of H_4NDTB-OMe followed the previously reported procedures without modification. [8]

Compound **6** was synthesized accordingly with a yield of 71 %. ^{1}H NMR (400 MHz, DMSO-d_6): δ = 8.40 (s, 2H), 11.02 (s, 1H) ppm.

Compound **7** was synthesized accordingly with a yield of 70 %. ^{1}H NMR (400 MHz, $CDCl_3$): δ = 3.98 (s, 3H), 8.42 (d, 2H) ppm.

Compound **8** was synthesized accordingly with a yield of 74 %. ^{1}H NMR (400 MHz, $CDCl_3$): δ = 3.62 (s, 2H), 3.80 (s, 3H), 6.82 (d, 2H) ppm.

Compound **9** was synthesized accordingly with a yield of 68 %. ^{1}H NMR (400 MHz, $CDCl_3$): δ =

1.42 (t, 6H), 3.05 (s, 3H), 4.40 (q, 4H), 6.83 (t, 2H), 7.68 (d, 4H), 8.09 (d, 4H) ppm.

Compound **10** was synthesized accordingly with a yield of 90 %. ^{1}H NMR (400 MHz, $CDCl_3$): δ = 2.93 (s, 3H), 5.12 (s, 2H), 6.63 (s, 2H), 7.67 (d, 4H), 8.02 (d, 4H), 12.91 (br, 2H) ppm.

Ligand **H_4NDTB-OMe** was synthesized accordingly with a yield of 82.3 %. ^{1}H NMR (400 MHz, DMSO-*d*6): δ = 3.19 (s, 6H), 7.64 (s, 4H), 7.76 (d, 8H), 8.05 (d, 8H), 8.68 (d, 8H), 13.02 (br, 4H) ppm. HRMS (m/z): [M]+ calculated. for $C_{56}H_{34}N_2O_{14}$: 958.20, found: 957.1933.

(4) Synthesis of H_4NDTB-F

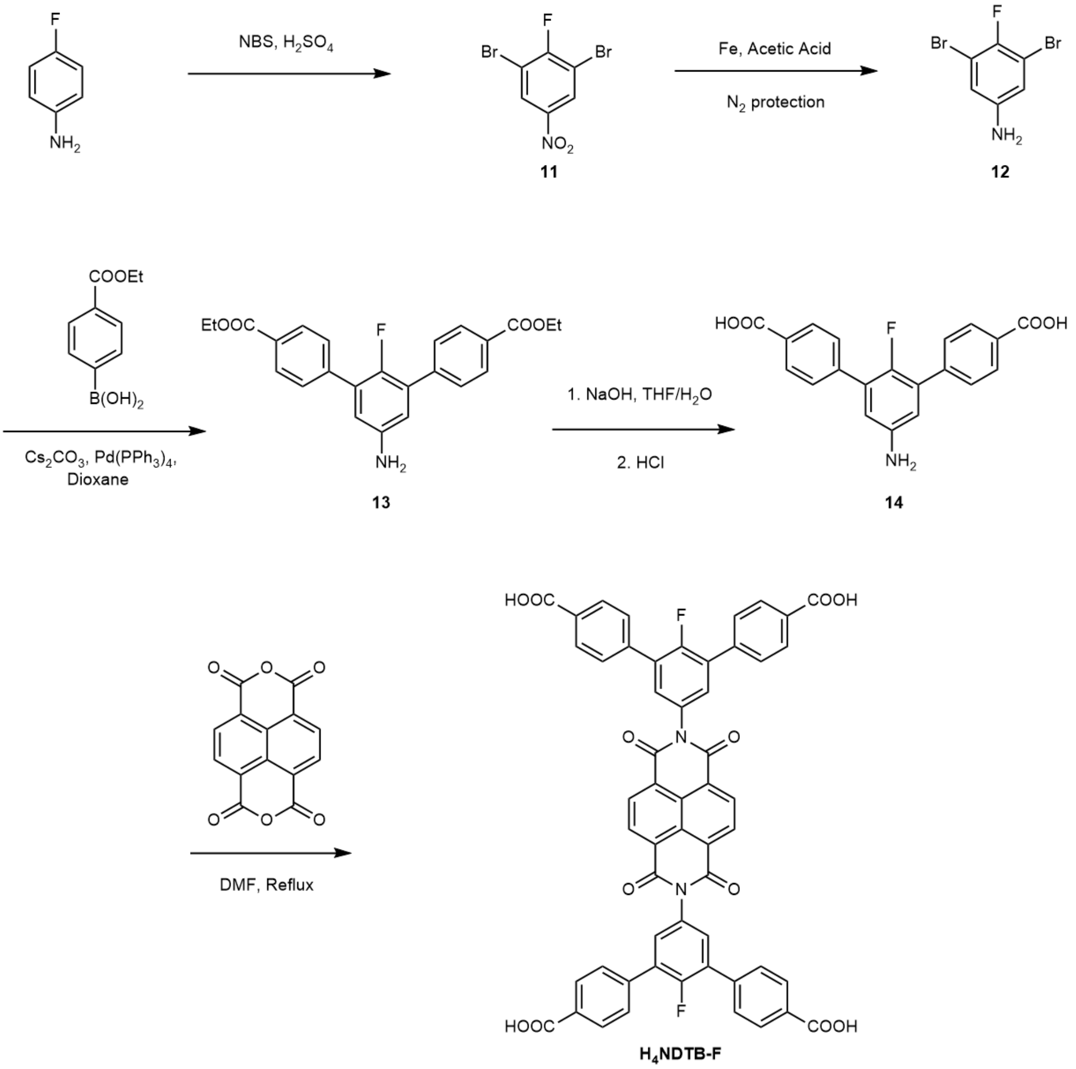

Figure S5. Synthesis of H_4NDTB-F.

Synthesis of 1,3-dibromo-2-fluoro-5-nitrobenzene **(11)**. 4-fluoroaniline (5.01 g, 35.4 mmol) was dissolved in 50 mL of concentrated H_2SO_4 solution and heated to 60 ℃. NBS (14.2 g, 78 mmol) was slowly added to the mixture over 10 mins and stirred for 2 h. After cooled to room temperature, the product was extracted from the mixture with toluene. The extraction solution was sequentially washed with pure water, $NaHCO_3$ aqueous solution, saturated NaCl aqueous solution, and used anhydrous $MgSO_4$ to remove the water. Solids of compound **11** was obtained after removed the toluene using a rotary evaporator (8.26 g, yield: 78 %). ^{1}H NMR (400 MHz, DMSO-*d*6): δ = 8.26 (d, 2H) ppm.

Synthesis of 3,5-dibromo-4-fluoroaniline **(12)**. Iron (5.2 g, 93.2 mmol) and compound **11** (4 g, 13.4 mmol) were added to a sealed 500 mL round-bottom flask and pumped vacuum. Degassed acetic acid (150 mL) was introduced into the flask and stirred for 1 h under room temperature and a N_2 atmosphere, followed by adding another 100 mL degassed acetic acid and stirred for 1 h. The reaction mixture was poured into ethyl acetate and washed sequentially with pure water, Na_2CO_3+$NaHCO_3$ aqueous solution, and saturated NaCl aqueous solution. Anhydrous $MgSO_4$ was used to remove the water before rotary evaporating the ethyl acetate. Solids of compound **12** was obtained in 86% yield (3.09 g), unpurified. ^{1}H NMR (400 MHz, $CDCl_3$): δ = 4.20 (s, 2H), 6.95 (d, 2H) ppm.

Synthesis of diethyl 5'-amino-2'-fluoro-[1,1':3',1''-terphenyl]-4,4''-dicarboxylate **(13)**. Compound **12** (5.38 g, 20 mmol), (4-(ethoxycarbonyl)phenyl)boronic acid (11.6 g, 60 mmol), K_2CO_3 (11 g, 80 mmol), and tetrakis(triphenylphosphine)palladium (1.156 g, 1 mmol) were added to a 500 mL Schlenk flask charged with a stir bar. The flask was pumped under vacuum and refilled with N_2 for three times, followed by transferring 200 mL of degassed 1,4-dioxane into the system. The reaction mixture was heated to 85 ℃ and stirred for 72 h under a N_2 atmosphere. After cooled to room temperature, the organic solvent was removed using a rotary evaporator, and the resulting mixture was transferred into water and extracted with dichloromethane (3 × 50 mL). The combined organic layers were dried over anhydrous $MgSO_4$, and the solvent was removed with rotary evaporator again. After purification by column chromatography on silica gel using ethyl

acetate/hexane (1:4 v/v) as eluent, compound **13** was obtained as a pale yellow solid from evaporating the fraction (5.86 g, yield: 72 %). ^{1}H NMR (400 MHz, $CDCl_3$): δ = 1.43 (t, 6H), 4.42 (q, 4H), 6.75 (t, 2H), 7.62 (d, 4H), 8.11 (d, 4H) ppm.

Synthesis of 5'-amino-2'-fluoro-[1,1':3',1''-terphenyl]-4,4''-dicarboxylic acid **(14)**. Compound **13** (0.57 g, 1.4 mmol) was dissolved in 20 mL of THF, to which 30 mL of 10 M NaOH aqueous solution was added. The mixture was stirred under reflux for 10 h, and then the organic solvent was removed using a rotary evaporator. The aqueous phase was acidified to pH = 2 using 6 M HCl aqueous solution. The resulting precipitate was collected *via* filtration, washed with water (200 mL), and dried under vacuum to afford compound **14** (0.45 g, 92 %). ^{1}H NMR (400 MHz, $CDCl_3$): δ = 6.85 (s, 2H), 7.22 (t, 2H), 7.65 (d, 4H), 8.02 (d, 4H), 12.91 (br, 2H) ppm.

Synthesis of 5',5''''-(1,3,6,8-tetraoxo-1,3,6,8-tetrahydrobenzo[lmn][3,8]phenanthroline-2,7-diyl)bis(2'-fluoro-[1,1':3',1''-terphenyl]-4,4''-dicarboxylic acid) **(H_4NDTB-F)**. Compound **14** (3.51 g, 10 mmol) and 1,4,5,8-tetracarboxydianhydride (1.34 g, 5.0 mmol) were added to a 250 mL Schlenk flask. The flask was pumped under vacuum and refilled with N_2 three times, followed by transferring 50 mL of degassed DMF into the system. The reaction mixture was heated to 160 ℃ and stirred for 12 h under a N_2 atmosphere. After cooled to room temperature, 5 mL of 1 mol/L HCl was added to the mixture, which was then poured into 500 mL of water. The product was collected by filtration, washed with water, ethanol and acetone, and dried under vacuum to give a light yellow solid of **H_4NDTB-F** (3.97 g, yield: 85%). ^{1}H NMR (400 MHz, DMSO-d_6): δ = 7.76 (d, 8H), 7.83 (d, 4H), 8.08 (d, 8H), 8.73 (s, 4H), 13.09 (br, 4H) ppm. HRMS (m/z): [M]+ calculated. for $C_{54}H_{28}F_2N_2O_{12}$: 934.16, found: 934.1631.

(5) Synthesis of H_4NDTB-OH

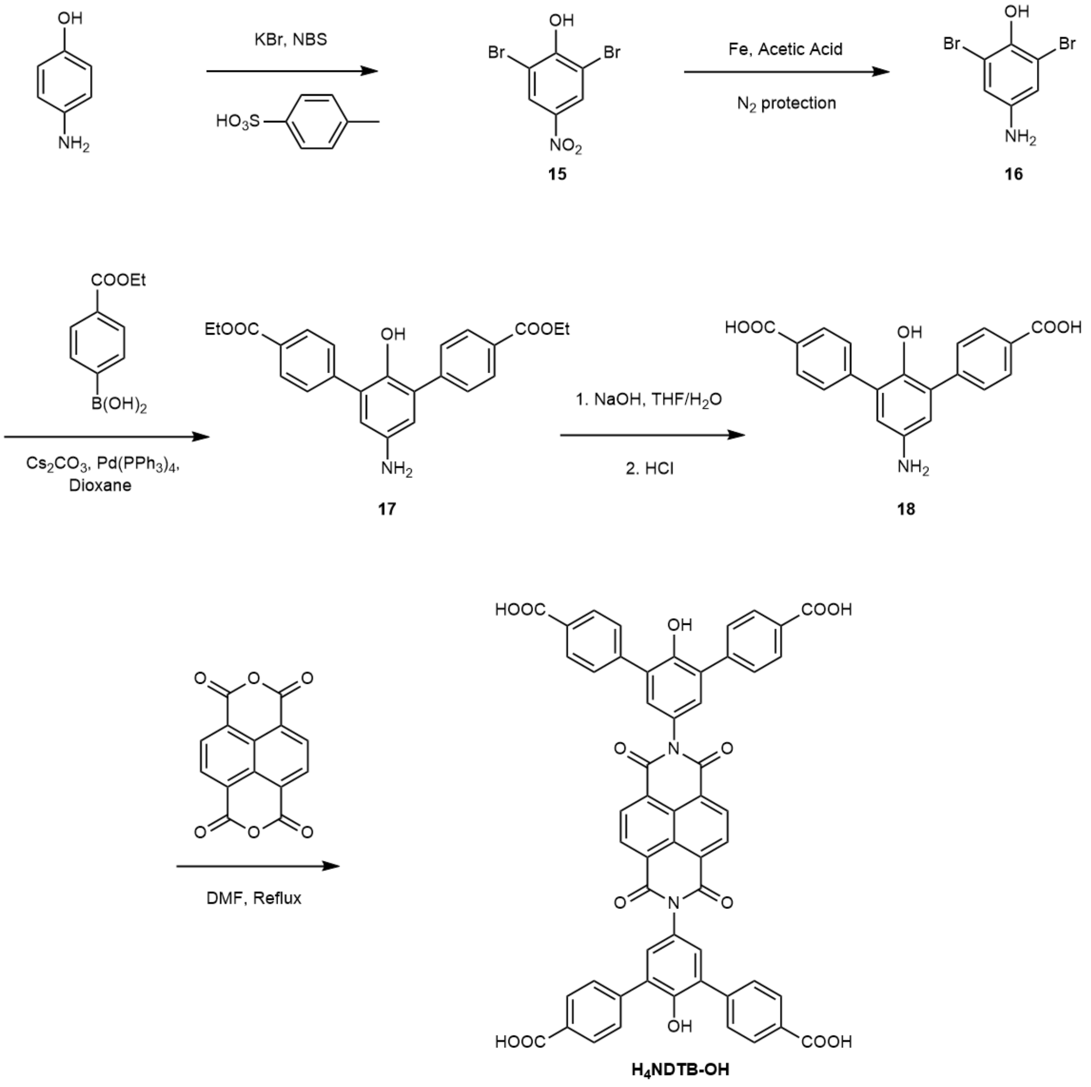

Figure S6. Synthesis of H_4NDTB-OH.

Synthesis of 2,6-dibromo-4-nitrophenol **(15)**. 4-aminophenol (2.78 g, 20 mmol), 4-methylbenzenesulfonic acid (7.61 g, 44.2 mmol), NBS (7.12 g, 40 mmol; slowly over 10 minutes) and KBr (7.14g, 60 mmol) were added to 50 mL of water (40 ℃) in the flask and stirred for 2 h, and brown solids could be observed. After cooled to room temperature, the filtered solids were washed with sodium metabisulfite aqueous solution (3×100 mL), and dried under vacuum to obtain the product (4.22 g, yield: 71 %). ^{1}H NMR (400 MHz, DMSO-*d*6) δ 8.39 (s, 2H) ppm, 11.04 (s, 1H).

Synthesis of 4-amino-2,6-dibromophenol **(16)**. Iron (5.2 g, 93.2 mmol) and compound **15** (6.5 g, 22 mmol) were added to a sealed 500 mL round-bottom flask and pumped vacuum. Degassed acetic acid (150 mL) was introduced into the flask and stirred for 1 h under room temperature and a N_2 atmosphere, followed by adding another 100 mL degassed acetic acid and stirred for 1 h. The reaction mixture was poured into ethyl acetate and washed sequentially with pure water, Na_2CO_3+$NaHCO_3$ aqueous solution, and saturated NaCl aqueous solution. Anhydrous $MgSO_4$ was used to remove the water before rotary evaporating the ethyl acetate. Solids of compound **16** was obtained in 83% yield (4.85 g), unpurified. ^{1}H NMR (400 MHz, $CDCl_3$): δ = 5.00 (s, 1H), 6.75 (d, 2H), 8.68 (s, 2H) ppm.

Synthesis of diethyl 5'-amino-2'-hydroxy-[1,1':3',1''-terphenyl]-4,4''-dicarboxylate **(17)**. Compound **16** (5.32 g, 20 mmol), (4-(ethoxycarbonyl)phenyl)boronic acid (11.6 g, 60 mmol), Cs_2CO_3 (26 g, 80 mmol), and tetrakis(triphenylphosphine)palladium (1.156 g, 1 mmol) were added to a 500 mL Schlenk flask charged with a stir bar. The flask was pumped under vacuum and refilled with N_2 for three times, followed by transferring 200 mL of degassed 1,4-dioxane into the system. The reaction mixture was heated to 85 ℃ and stirred for 72 h under a N_2 atmosphere. After cooled to room temperature, the organic solvent was removed using a rotary evaporator, and the resulting mixture was transferred into water and extracted with dichloromethane (3 × 50 mL). The combined organic layers were dried over anhydrous $MgSO_4$, and the solvent was removed with rotary evaporator again. After purification by column chromatography on silica gel using ethyl acetate/hexane (1:4 v/v) as eluent, compound **17** was obtained as a pale-yellow solid from evaporating the fraction (5.35 g, yield: 66 %). ^{1}H NMR (400 MHz, $CDCl_3$): δ = 1.42 (t, 6H), 4.40 (q, 4H), 6.71 (t, 2H), 7.63 (d, 4H), 8.13 (d, 4H) ppm.

Synthesis of 5'-amino-2'-hydroxy-[1,1':3',1''-terphenyl]-4,4''-dicarboxylic acid **(18)**. Compound **17** (0.56 g, 1.4 mmol) was dissolved in 20 mL of THF, to which 30 mL of 10 M NaOH aqueous solution was added. The mixture was stirred under reflux for 10 h, and then the organic solvent was removed using a rotary evaporator. The aqueous phase was acidified to pH = 2 using 6 M HCl aqueous solution. The resulting precipitate was collected *via* filtration, washed with water (200

mL), and dried under vacuum to afford compound **18** (0.43 g, 88 %). ^1H NMR (400 MHz, $CDCl_3$): δ = 7.05 (s, 2H), 7.66 (d, 4H), 8.00 (d, 4H) ppm.

Synthesis of 5',5''''-(1,3,6,8-tetraoxo-1,3,6,8-tetrahydrobenzo[lmn][3,8]phenanthroline-2,7-diyl)bis(2'-hydroxy-[1,1':3',1''-terphenyl]-4,4''-dicarboxylic acid) **(H_4NDTB-OH)**. Compound **18** (3.49 g, 10 mmol) and 1,4,5,8-tetracarboxydianhydride (1.34 g, 5.0 mmol) were added to a 250 mL Schlenk flask. The flask was pumped under vacuum and refilled with N_2 three times, followed by transferring 50 mL of degassed DMF into the system. The reaction mixture was heated to 160 ℃ and stirred for 12 h under a N_2 atmosphere. After cooled to room temperature, 5 mL of 1 mol/L HCl was added to the mixture, which was then poured into 500 mL of water. The product was collected by filtration, washed with water, ethanol and acetone, and dried under vacuum to give a light yellow solid of **H_4NDTB-OH** (3.72 g, yield: 80%).^1H NMR (400 MHz, DMSO-d_6): δ = 7.50 (s, 4H), 7.71 (d, 8H), 8.02 (d, 8H), 8.58 (s, 4H) ppm. HRMS (m/z): [M]+ calculated. for $C_{54}H_{30}N_2O_{14}$: 930.17, found: 930.1724.

Syntheses of H_2TPDC-X

The syntheses of all three types of H_2TPDC-X followed the previously reported procedures without modification.[8]

Ligand **H_2TPDC-TDA** was synthesized accordingly with a yield of 93 %. ^1H NMR (400 MHz, DMSO-d_6): δ = 8.10 (d, 2H), 8.15 (d, 4H), 8.19 (d, 4H), 13.04 (br, 2H) ppm. HRMS (m/z): [M]+ calculated. for $C_{20}H_{12}N_2O_4S$: 376.05, found: 376.0506.

Ligand **H_2TPDC-AN** was synthesized accordingly with a yield of 94 %. ^1H NMR (400 MHz, DMSO-d_6): δ 7.47 (d, 4H), 7.55 (d, 4H), 7.63 (d, 4H), 8.22 (d, 4H), 13.14 (br, 2H) ppm. HRMS (m/z): [M]+ calculated. for $C_{28}H_{18}O_4$: 418.12, found: 418.1200.

Ligand **H_2TPDC-Py** was synthesized accordingly with a yield of 94 %. ^1H NMR (400 MHz, DMSO-d_6): δ 7.98 (d, 1H), 8.06 (d, 2H), 8.09 (d, 3H), 8.22 (d, 2H), 8.31 (d, 2H), 9.12 (s, 1H), 13.02 (br, 2H) ppm. HRMS (m/z): [M]+ calculated. for $C_{19}H_{13}NO_4$: 319.08, found: 319.0828.

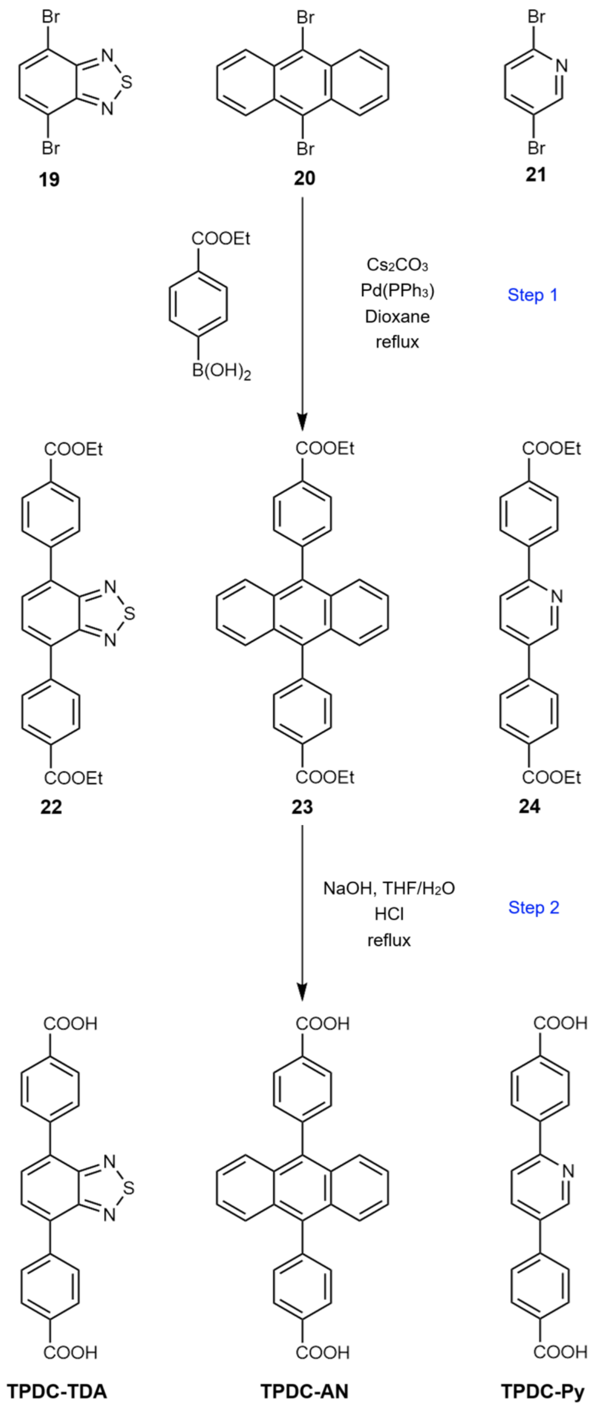

Figure S7. Syntheses of H_2TPDC-X (X = TDA, AN or Py).

Syntheses of NKM-908-R crystals

A typical synthesis of **NKM-908-OMe** was demonstrated as follow: $ZrCl_4$ (20 mg), H_4NDTB-OMe (10 mg), benzoic acid (225 mg), and DMF (3 mL) were charged in a 5 mL Pyrex vial. The mixture was heated in a 120 °C oven for 72 h. After cooling down to room temperature, the pale yellow crystals of NKM-908-OMe were harvested (yield: 65 %).

The rest four types of **NKM-908-R** were synthesized accordingly with modifications to the H_4NDTB-R ligands used during the syntheses.

Syntheses of NKM-908-R-TPDC-X crystals

A typical synthesis of **NKM-908-OMe-TPDC-TDA** was demonstrated as follow: NKM-908-OMe (100 mg), H_2TPDC-TDA (200 mg) and DMF (20 mL) were charged in a Pyrex vial. The mixture was heated in a 100 °C oven for 24 h. After cooling down to the room temperature, the crystals were washed three times with DMF, and then placed in 15 mL of DMF mixed with 200 μL of 1 M HCl. The mixture was left at 100 ° C in a conventional oven overnight. After cooling down to room temperature, the pale yellow crystals of **NKM-908-OMe-TPDC-TDA** were harvested (yield based on NKM-908-OMe: 96 %).

The rest fourteen types of **NKM-908-R-TPDC-X** were synthesized accordingly with modifications to the NKM-908-R and H_2TPDC-X ligands used during the syntheses.

Syntheses of NKM-906-R crystals

A typical synthesis of **NKM-906-OMe** was demonstrated as follow: $ZrCl_4$ (20 mg), H_4NDTB-OMe (10 mg), benzoic acid (600 mg), and DMF (3 mL) were charged in a 20 mL Pyrex vial. The mixture was heated in a 120 °C oven for 72 h. After cooling down to room temperature, the pale yellow crystals of NKM-906-OMe were harvested (yield: 68 %).

The rest four types of **NKM-906-R** were synthesized accordingly with modifications to the H_4NDTB-R ligands used during the syntheses.

Syntheses of NKM-906-R-TPDC-X crystals

A typical synthesis of **NKM-906-OMe-TPDC-TDA** was demonstrated as follow: NKM-906-

OMe (100 mg), H_2TPDC-TDA (200 mg) and DMF (20 mL) were charged in a Pyrex vial. The mixture was heated in a 100 °C oven for 24 h. After cooling down to the room temperature, the crystals were washed three times with DMF, and then placed in 15 mL of DMF mixed with 200 μL of 1 M HCl. The mixture was left at 100 °C in a conventional oven overnight. After cooling down to room temperature, the pale yellow crystals of **NKM-906-OMe-TPDC-TDA** were harvested (yield based on NKM-906-OMe: 96 %).

The rest fourteen types of **NKM-906-R-TPDC-X** were synthesized accordingly with modifications to the NKM-906-R and H_2TPDC-X ligands used during the syntheses.

Fabrication of MOF thin films

Typical procedures of the **NKM-908-OMe thin film** fabrication were demonstrated as follow: The ITO glass was cut into strip shape ($60 \times 7\ mm^2$), and was cleaned in soap water, ethanol, and acetone under ultrasonic condition for 15 min each. H_4NDTB-OMe (90.8 mg, 0.094 mmol), $ZrOCl_2 \cdot 8H_2O$ (99.9 mg, 0.31 mmol) and benzoic acid (1.275 g) were dissolved in a mixture of anhydrous DMF (10 mL) and anhydrous methanol (1 mL) in a 20 ml vial. The pre-cleaned ITO substrate was submerged into this solution obliquely, with the conductive side facing downwards. The reaction solution was then heated to 120 °C in a conventional oven for 3 days. After cooling down to room temperature, the adherent solids on the non-conductive side were completely wiped off using a DMF-soaked cotton swab, followed by gently rinsing the MOF film with anhydrous DMF. The film was then dried under a stream of nitrogen.

The rest four types of **NKM-908-R thin films** were synthesized accordingly with modifications to the H_4NDTB-R ligands used during the syntheses.

Typical procedures of the **NKM-908-OMe-TPDC-TDA thin film** fabrication were demonstrated as follow: The prepared NKM-908-OMe film was immersed in a 20 mL vial obliquely containing the TPDC-TDA/DMF solution and heated at 100 ℃ for 1 day. After cooling down to room temperature, the film was washed three times with DMF, and then placed in 15 mL of DMF mixed with 200 μL of 1 M HCl. The mixture was left at 100 ° C in a conventional oven overnight. After

cooling down to room temperature, the film was gently rinsed with anhydrous DMF and dried under a nitrogen flow.

The rest fourteen types of **NKM-908-R-TPDC-X thin films** were synthesized accordingly with modifications to the NKM-906-R films used and H_2TPDC-X ligands used during the syntheses.

Typical procedures of the **NKM-906-OMe thin film** fabrication were demonstrated as follow: The ITO glass was cut into strip shape (60 × 7 mm^2), and was cleaned in soap water, ethanol, and acetone under ultrasonic condition for 15 min each. H_4NDTB-OMe (90.8 mg, 0.094 mmol), $ZrOCl_2$ 8H_2O (99.9 mg, 0.31 mmol) and benzoic acid (3.4 g) were dissolved in a mixture of anhydrous DMF (10 mL) and anhydrous methanol (1 mL) in a 20 ml vial. The pre-cleaned ITO substrate was submerged into this solution obliquely, with the conductive side facing downwards. The reaction solution was then heated to 120 °C in a conventional oven for 3 days. After cooling down to room temperature, the adherent solids on the non-conductive side were completely wiped off using a DMF-soaked cotton swab, followed by gently rinsing the MOF film with anhydrous DMF. The film was then dried under a stream of nitrogen.

The rest four types of **NKM-906-R thin films** were synthesized accordingly with modifications to the H_4NDTB-R ligands used during the syntheses.

Typical procedures of the **NKM-906-OMe-TPDC-TDA thin film** fabrication were demonstrated as follow: The prepared NKM-906-OMe film was immersed in a 20 mL vial obliquely containing the TPDC-TDA/DMF solution and heated at 100 ℃ for 1 day. After cooling down to room temperature, the film was washed three times with DMF, and then placed in 15 mL of DMF mixed with 200 μL of 1 M HCl. The mixture was left at 100 ° C in a conventional oven overnight. After cooling down to room temperature, the film was gently rinsed with anhydrous DMF and dried under a nitrogen flow.

The rest fourteen types of **NKM-906-R-TPDC-X thin films** were synthesized accordingly with modifications to the NKM-906-R films used and H_2TPDC-X ligands used during the syntheses.

Typical procedures of the **organic linker thin film** fabrication were demonstrated as follow: H_4NDTB-OMe (5 mg) and H_2TPDC-Py (5 mg) were grinded into fine powder and dissolved in a mixture solution of ethanol+water+Nafion (500/500/50 μL), subjected to low-temperature ultrasonication for 20 minutes to obtain a uniformly dispersed ink. Then, 50 μL of such ink was dropped onto the ITO glass using a pipette and allowed to air dry naturally.

Supplementary Figures and Tables

Table S1. SCXRD data collection and structure solutions for NKM-908-OMe, NKM-908-Me, NKM-908-OH, NKM-906-OMe, NKM-908-H-TPDC-4F and NKM-906-H-TPDC-4F.

Code	NKM-908-OMe [8]	NKM-908-Me	NKM-908-OH	NKM-906-OMe	NKM-908-OMe-TPDC-4F [8]	NKM-906-H-TPDC-4F
Formula	$C_{56}H_{30}N_2O_{22}Zr_3$	$C_{56}H_{30}N_2O_{20}Zr_3$	$C_{54}H_{24}N_2O_{22}Zr_3$	$C_{56}H_{30}N_2O_{22}Zr_3$	$C_{66}H_{34}F_2N_2O_{21}Zr_3$	$C_{86}H_{30}F_4N_2O_{20}Zr_3$
Formula weight	1356.52	1324.48	1326.41	1351.44	1502.66	1760.78
Temperature/K	110(2)	100.0(3)	110(2)	298	293	293
Crystal system	Hexagonal	Hexagonal	Hexagonal	Orthorhombic	Hexagonal	Orthorhombic
Space group	*P6/mmm*	*P6/mmm*	*P6/mmm*	*P/mmm*	*P6/mmm*	*C/mmm*
a / Å	40.2850(15)	40.385(2)	40.0160(5)	23.5213(14)	40.54(15)	22.7(4)
b / Å	40.2850	40.385	40.0160	23.5213	40.54(15)	32.6(3)
c / Å	23.6990(16)	23.7573(10)	24.3387(6)	33.754(2)	23.45(14)	23.0(2)
α / °	90	90	90	90	90	90
β / °	90	90	90	90	90	90
γ / °	120	120	120	90	120	90
Volume / Å^3	33309(5)	35555(4)	33751.5(19)	18674.2(16)	33364(324)	17019(353)
Z	6	6	6	4	6	4
ρ_{calc} / g cm^{-3}	0.4057	0.393	0.4057	0.481	0.449	0.687
μ / mm^{-1}	1.315	1.296	1.294	1.563	0.000	0.000
F(000)	4056	3960	3948	2684	1617.0	1277
Crystal size/mm^3	0.08 × 0.02 × 0.02	0.10 × 0.02 × 0.03	0.15 × 0.03 × 0.03	0.13 × 0.04 × 0.02	0.011 × 0.0008 × 0.0009	0.006 × 0.001 × 0.001
Radiation	CuK$_\alpha$ (λ = 1.54184)	CuK$_\alpha$ (λ = 1.54184)	CuK$_\alpha$ (λ = 1.54184)	CuK$_\alpha$ (λ = 1.54184)	transmission electron microscope (λ = 0.0251)	transmission electron microscope (λ = 0.0251)
2Θ range for data collection/ °	4.386 to 111.732	7.516 to 77.054	4.436 to 138.644	5.314 to 109.194	0.188 to 1.798	0.192 to 1.798
Reflections collected	165711	87054	179572	50454	153511	38264
Independent reflections	7952 [R_{int} = 0.4809, R_{sigma} = 0.2485]	12128 [R_{int} = 0.2977, R_{sigma} = 0.1290]	12037 [R_{int} = 0.2373, R_{sigma} = 0.0774]	11262 [R_{int} = 0.1518, R_{sigma} = 0.1459]	46571 [R_{int} =1.1439, R_{sigma} = 0.9154]	9015 [R_{int} = 0.8811, R_{sigma} = 0.8245]
Data/restraints/parameters	7952/88/154	12128/59/168	12037/57/176	11262/99/111	46571/408/276	9015/224/105
Goodness-of-fit on F^2	1.025	1.339	0.917	1.432	0.921	0.859
Final R indexes [I>=2σ (I)]	R_1 = 0.1325, wR_2 = 0.3274	R_1 = 0.1168, wR_2 = 0.3058	R_1 = 0.1081, wR_2 = 0.2808	R_1 = 0.1484, wR_2 = 0.3898	R_1 = 0.3200, wR_2 = 0.5871	R_1 = 0.2715, wR_2 = 0.5176
Final R indexes [all data]	R_1 = 0.2029, wR_2 = 0.3805	R_1 = 0.2030, wR_2 = 0.4028	R_1 = 0.1653, wR_2 = 0.3533	R_1 = 0.2269, wR_2 = 0.4723	R_1 = 0.6667, wR_2 = 0.7743	R_1 = 0.2029, wR_2 = 0.6163
Largest diff. peak/hole / e Å^3	0.67/-0.52	1.28/-0.55	0.67/-0.96	2.816/-1.498	0.42/-0.22	0.385/-0.352

Table S2. Elemental analyses results of all forty MOFs.

Compound	C $\%_{calc}$	C $\%_{anal}$	H $\%_{calc}$	H $\%_{anal}$	N $\%_{calc}$	N $\%_{anal}$
NKM-908-H	49.72	48.24	2.63	2.67	2.15	2.22
NKM-908-Me	50.47	49.39	2.87	2.76	2.10	2.13
NKM-908-OMe	49.29	50.15	2.81	2.95	2.05	2.22
NKM-908-F	48.38	47.98	2.41	2.45	2.09	2.18
NKM-908-OH	48.53	47.34	2.56	2.48	2.10	2.19
NKM-906-H	49.72	48.50	2.63	2.59	2.15	2.09
NKM-906-Me	50.47	49.35	2.87	2.79	2.10	2.05
NKM-906-OMe	49.29	49.67	2.81	2.77	2.05	2.11
NKM-906-F	48.38	47.70	2.41	2.38	2.09	2.04
NKM-906-OH	48.53	47.83	2.56	2.61	2.10	2.15
NKM-908-H-AN	54.06	53.87	2.67	2.59	1.85	1.91
NKM-908-Me-AN	54.64	53.91	2.88	2.96	1.82	1.91
NKM-908-OMe-AN	53.52	53.02	2.82	2.84	1.78	1.79
NKM-908-F-AN	52.80	53.01	2.48	2.51	1.81	1.85
NKM-908-OH-AN	52.94	53.74	2.61	2.67	1.82	1.87
NKM-906-H-AN	59.58	58.69	2.80	2.85	1.69	1.62
NKM-906-Me-AN	60.02	60.29	3.00	3.06	1.67	1.77
NKM-906-OMe-AN	58.90	58.29	2.94	2.99	1.64	1.71
NKM-906-F-AN	58.38	58.01	2.51	2.60	1.66	1.62
NKM-906-OH-AN	58.45	57.91	2.75	2.79	1.66	1.73
NKM-908-H-Py	53.37	52.21	2.64	2.59	2.45	2.39
NKM-908-Me-Py	53.99	52.29	2.87	2.93	2.40	2.39
NKM-908-OMe-Py	52.83	53.91	2.81	2.89	2.35	2.41
NKM-908-F-Py	52.05	51.51	2.44	2.42	2.39	2.49
NKM-908-OH-Py	52.20	51.79	2.59	2.62	2.40	2.32
NKM-906-H-Py	56.43	55.91	2.66	2.73	2.70	2.77
NKM-906-Me-Py	56.95	55.74	2.87	2.89	2.66	2.74
NKM-906-OMe-Py	55.82	55.02	2.81	2.89	2.60	2.62
NKM-906-F-Py	55.15	55.31	2.47	2.56	2.39	2.47
NKM-906-OH-Py	55.29	53.99	2.61	2.68	2.65	2.69
NKM-908-H-TDA	52.73	51.43	2.56	2.60	2.88	2.89
NKM-908-Me-TDA	53.35	51.97	2.78	2.77	2.83	2.89
NKM-908-OMe-TDA	52.23	51.86	2.72	2.76	2.77	2.85
NKM-908-F-TDA	51.46	51.50	2.36	2.39	2.81	2.79
NKM-908-OH-TDA	51.60	51.12	2.50	2.56	2.82	2.87
NKM-906-H-TDA	55.18	54.71	2.50	2.58	3.48	3.53
NKM-906-Me-TDA	55.70	53.99	2.71	2.78	3.41	3.45
NKM-906-OMe-TDA	54.63	52.89	2.65	2.69	3.35	3.42
NKM-906-F-TDA	53.97	52.74	2.33	2.39	3.40	3.47
NKM-906-OH-TDA	54.10	52.97	2.45	2.50	3.41	3.43

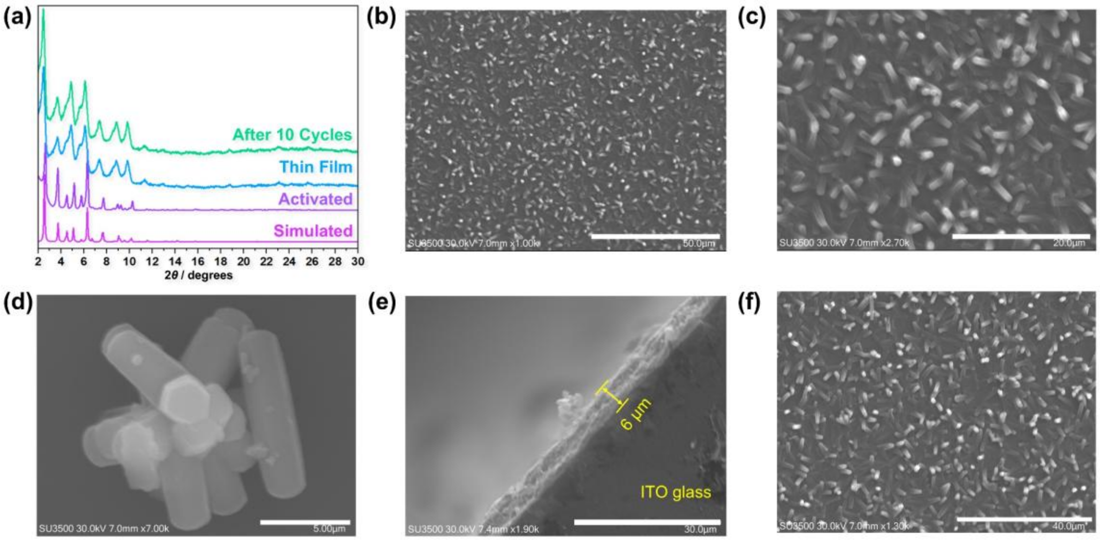

Figure S8. General characterizations of NKM-908-H showing (a) PXRD patterns of the simulated (magenta), activated crystals (purple), thin film (cyan) and thin film after 10 cycles of electrochromic tests (green); and SEM images of (b) film surface, (c) film surface zoomed-in, (d) crystals scratched off from the film, (e) cross-section of the film, and (f) film surface after 10 cycles of electrochromic tests.

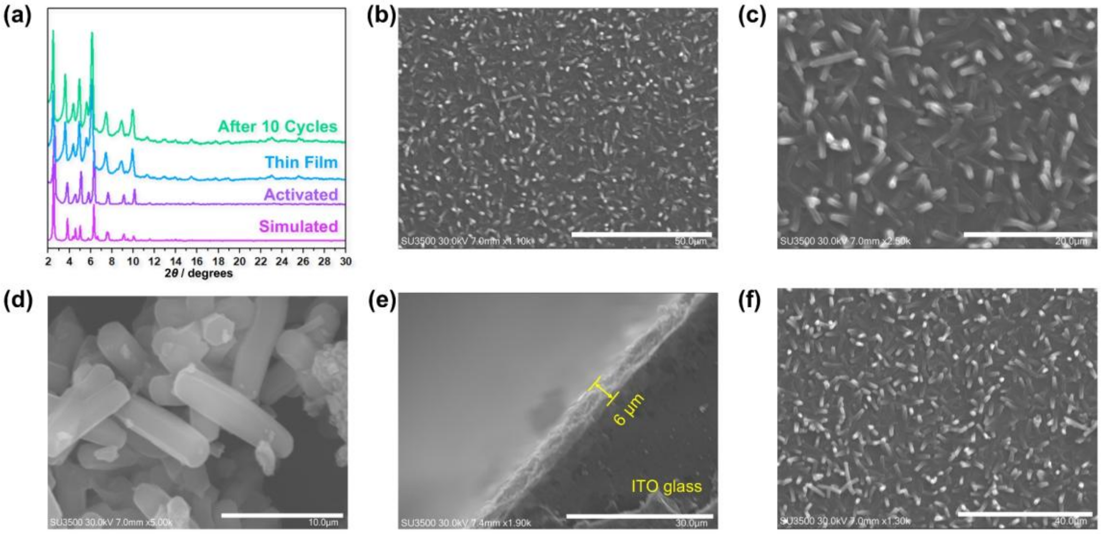

Figure S9. General characterizations of NKM-908-Me showing (a) PXRD patterns of the simulated (magenta), activated crystals (purple), thin film (cyan) and thin film after 10 cycles of electrochromic tests (green); and SEM images of (b) film surface, (c) film surface zoomed-in, (d) crystals scratched off from the film, (e) cross-section of the film, and (f) film surface after 10 cycles of electrochromic tests.

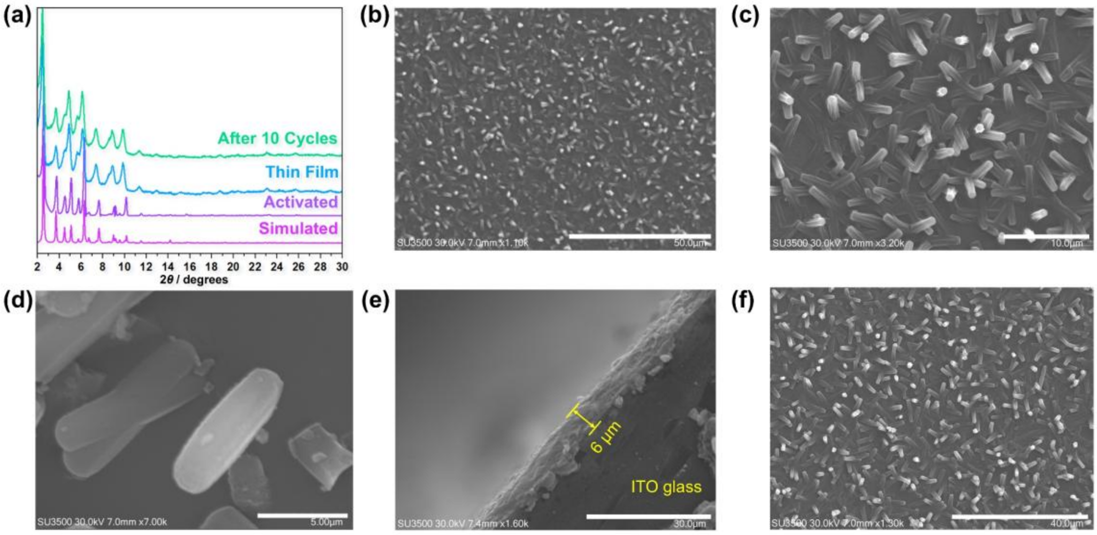

Figure S10. General characterizations of NKM-908-OMe showing (a) PXRD patterns of the simulated (magenta), activated crystals (purple), thin film (cyan) and thin film after 10 cycles of electrochromic tests (green); and SEM images of (b) film surface, (c) film surface zoomed-in, (d) crystals scratched off from the film, (e) cross-section of the film, and (f) film surface after 10 cycles of electrochromic tests.

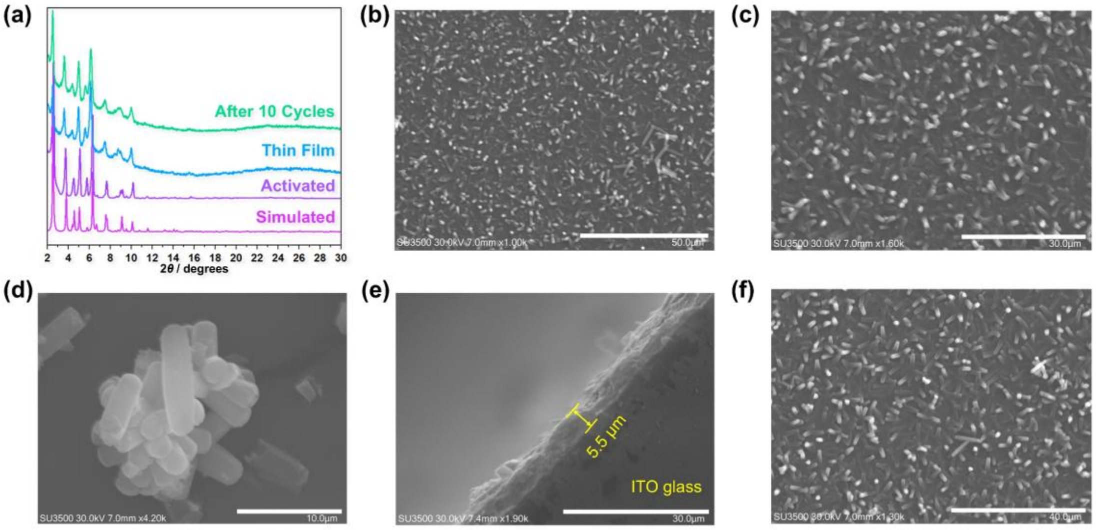

Figure S11. General characterizations of NKM-908-F showing (a) PXRD patterns of the simulated (magenta), activated crystals (purple), thin film (cyan) and thin film after 10 cycles of electrochromic tests (green); and SEM images of (b) film surface, (c) film surface zoomed-in, (d) crystals scratched off from the film, (e) cross-section of the film, and (f) film surface after 10 cycles of electrochromic tests.

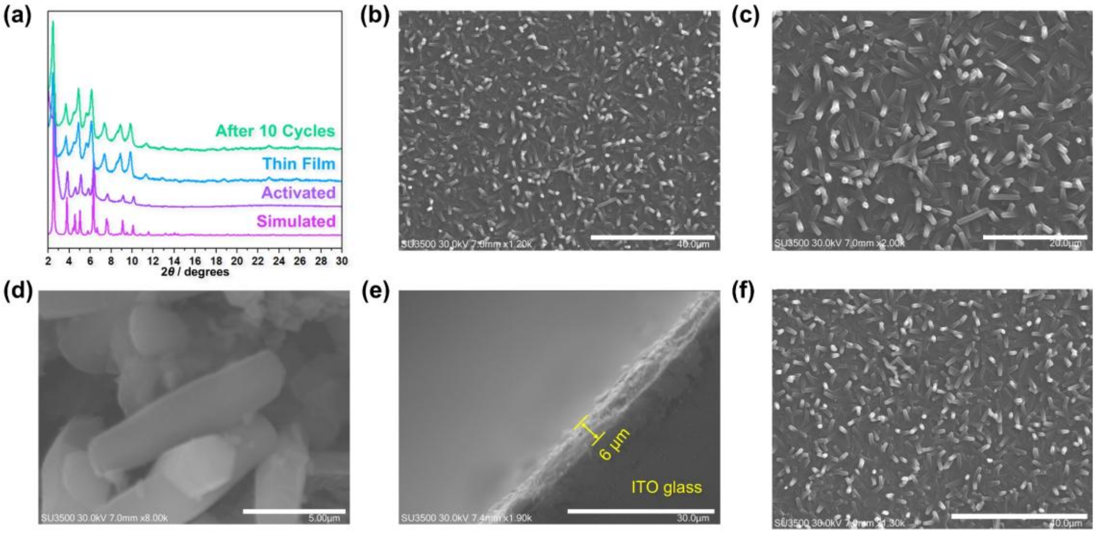

Figure S12. General characterizations of NKM-908-OH showing (a) PXRD patterns of the simulated (magenta), activated crystals (purple), thin film (cyan) and thin film after 10 cycles of electrochromic tests (green); and SEM images of (b) film surface, (c) film surface zoomed-in, (d) crystals scratched off from the film, (e) cross-section of the film, and (f) film surface after 10 cycles of electrochromic tests.

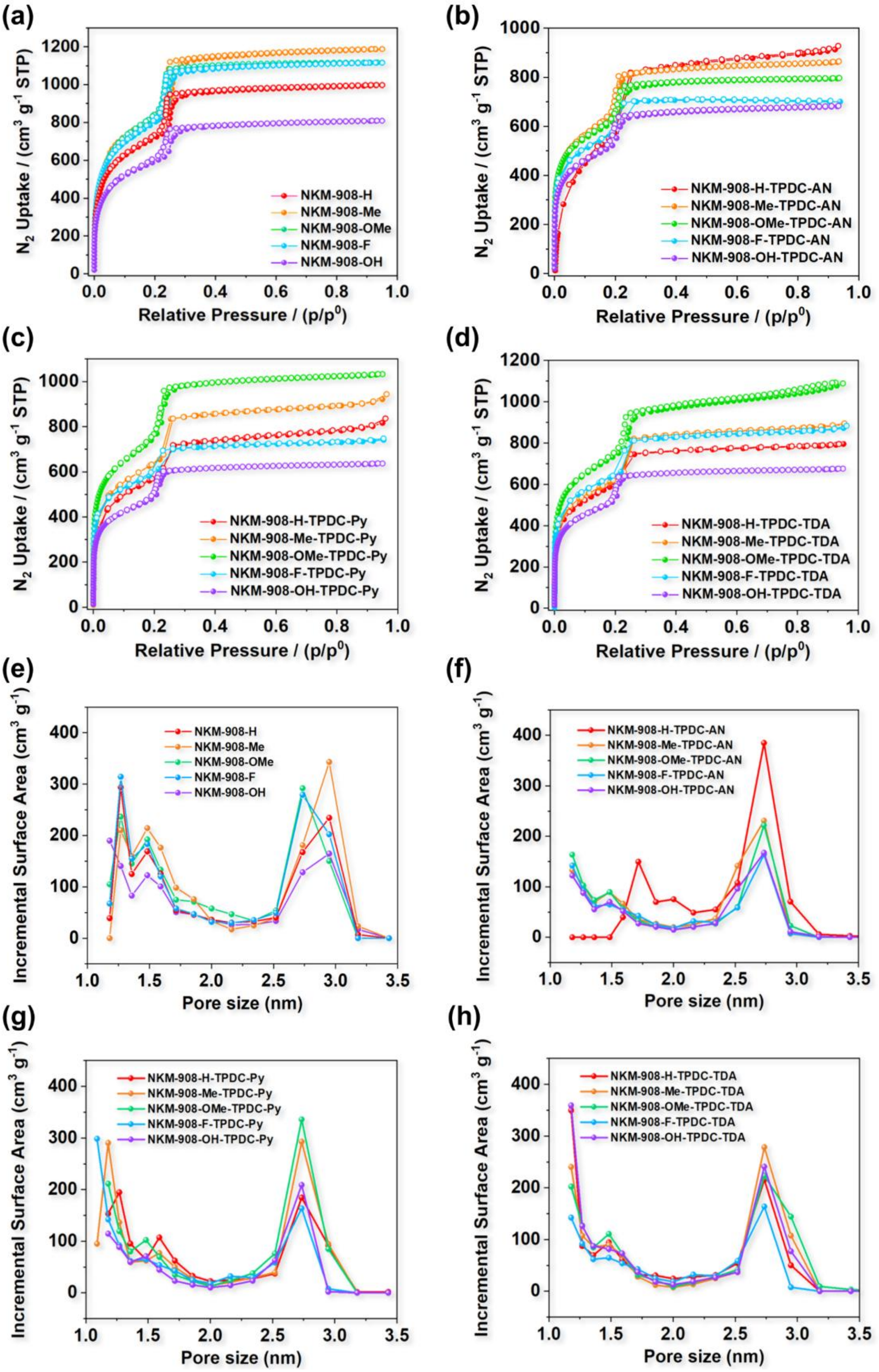

Figure S13. (a-d) Volumetric N_2 adsorption/desorption isotherms and (e-h) the corresponding pore size distributions for NKM-908-R series and NKM-908-R-TPDC-X series.

Table S3. Summary of the calculated Brunauer–Emmett–Teller (BET) specific surface areas for NKM-908-R series and NKM-908-R-TPDC-X series.

Compound	BET surface area (m^2 g^{-1})	Compound	BET surface area (m^2 g^{-1})
NKM-908-H	2530	NKM-908-H-TPDC-AN	2245
NKM-908-Me	2892	NKM-908-Me-TPDC-AN	2324
NKM-908-OMe	2925	NKM-908-OMe-TPDC-AN	2222
NKM-908-F	2824	NKM-908-F-TPDC-AN	2260
NKM-908-OH	2101	NKM-908-OH-TPDC-AN	1873
NKM-908-H-TPDC-Py	2319	NKM-908-H-TPDC-TDA	2095
NKM-908-Me-TPDC-Py	2691	NKM-908-Me-TPDC-TDA	2548
NKM-908-OMe-TPDC-Py	2669	NKM-908-OMe-TPDC-TDA	2630
NKM-908-F-TPDC-Py	2244	NKM-908-F-TPDC-TDA	2567
NKM-908-OH-TPDC-Py	1727	NKM-908-OH-TPDC-TDA	1846

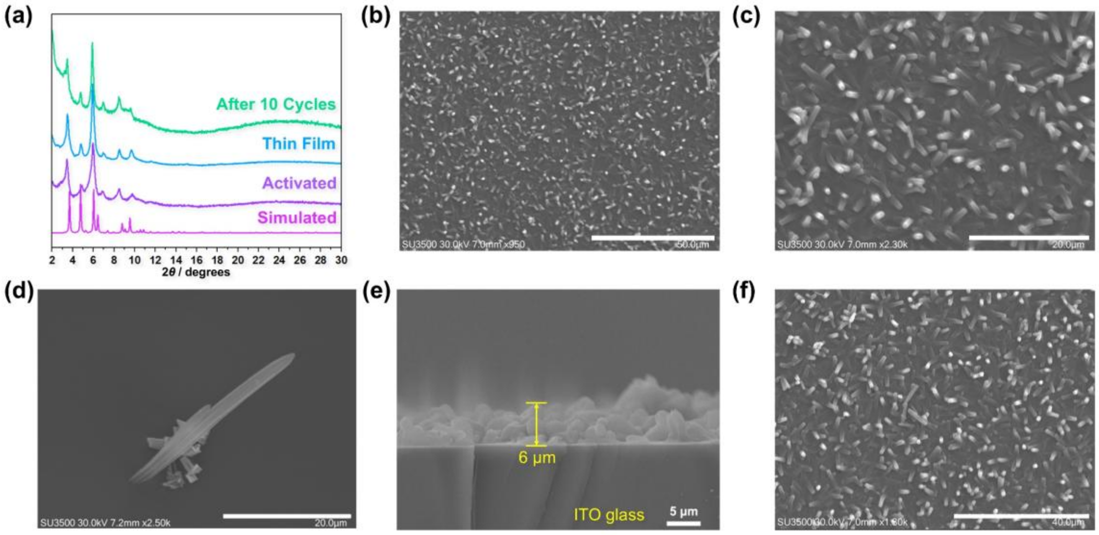

Figure S14. General characterizations of NKM-906-H showing (a) PXRD patterns of the simulated (magenta), activated crystals (purple), thin film (cyan) and thin film after 10 cycles of electrochromic tests (green); and SEM images of (b) film surface, (c) film surface zoomed-in, (d) crystals scratched off from the film, (e) cross-section of the film, and (f) film surface after 10 cycles of electrochromic tests.

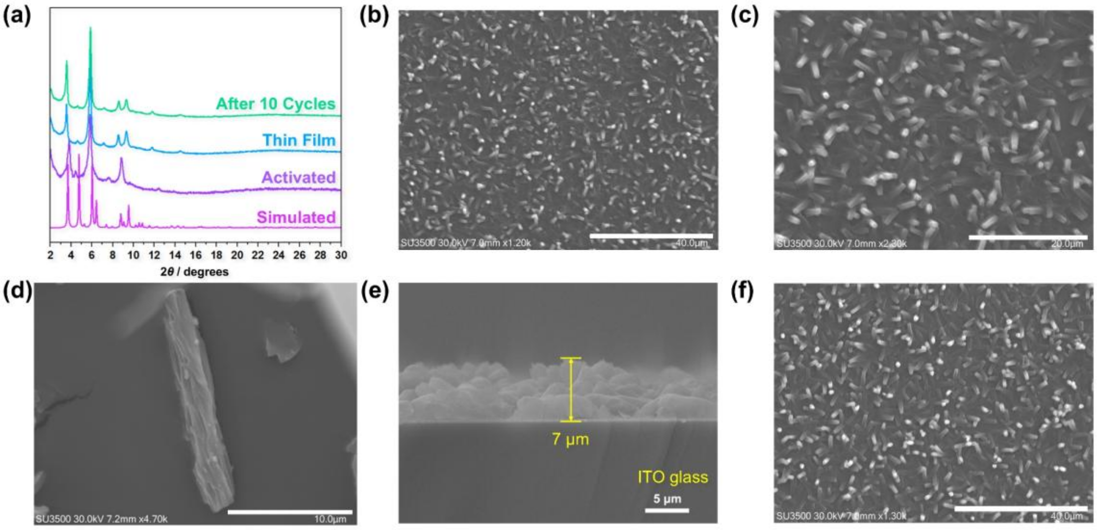

Figure S15. General characterizations of NKM-906-Me showing (a) PXRD patterns of the simulated (magenta), activated crystals (purple), thin film (cyan) and thin film after 10 cycles of electrochromic tests (green); and SEM images of (b) film surface, (c) film surface zoomed-in, (d) crystals scratched off from the film, (e) cross-section of the film, and (f) film surface after 10 cycles of electrochromic tests.

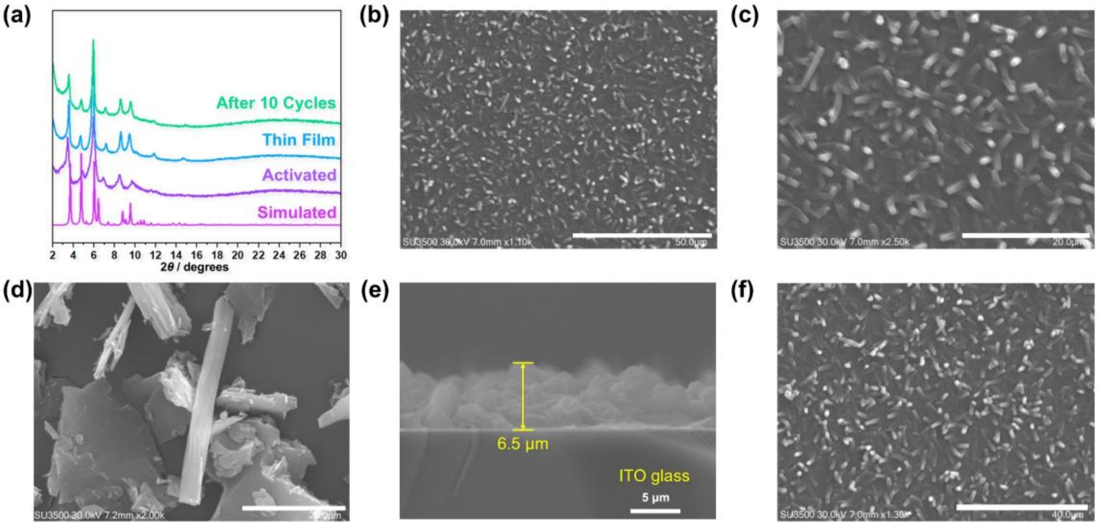

Figure S16. General characterizations of NKM-906-OMe showing (a) PXRD patterns of the simulated (magenta), activated crystals (purple), thin film (cyan) and thin film after 10 cycles of electrochromic tests (green); and SEM images of (b) film surface, (c) film surface zoomed-in, (d) crystals scratched off from the film, (e) cross-section of the film, and (f) film surface after 10 cycles of electrochromic tests.

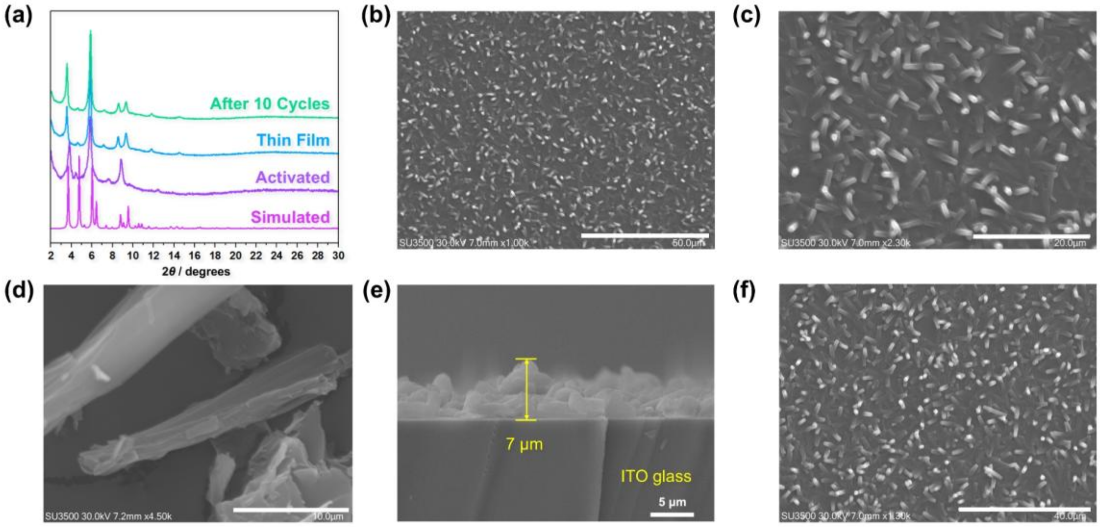

Figure S17. General characterizations of NKM-906-F showing (a) PXRD patterns of the simulated (magenta), activated crystals (purple), thin film (cyan) and thin film after 10 cycles of electrochromic tests (green); and SEM images of (b) film surface, (c) film surface zoomed-in, (d) crystals scratched off from the film, (e) cross-section of the film, and (f) film surface after 10 cycles of electrochromic tests.

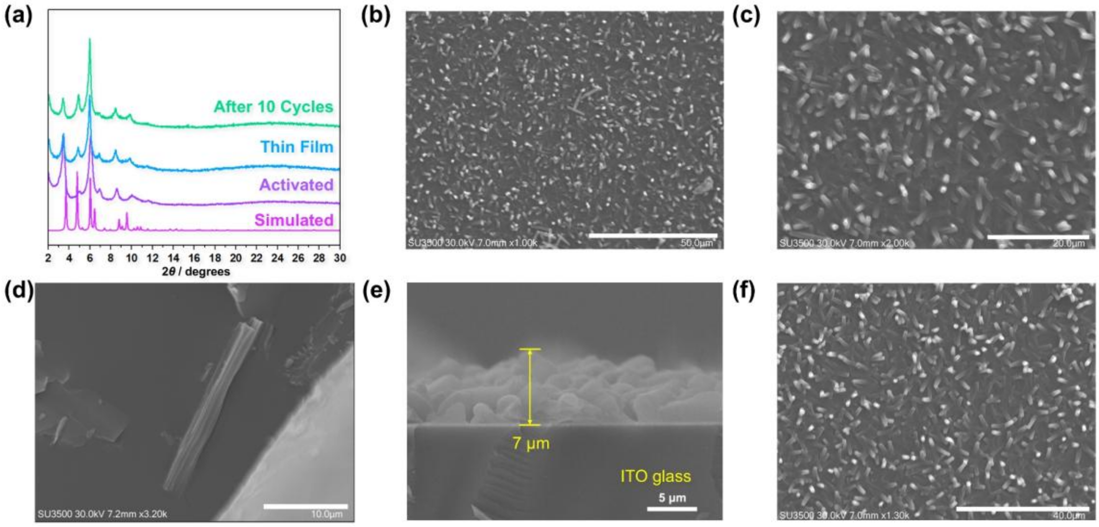

Figure S18. General characterizations of NKM-906-OH showing (a) PXRD patterns of the simulated (magenta), activated crystals (purple), thin film (cyan) and thin film after 10 cycles of electrochromic tests (green); and SEM images of (b) film surface, (c) film surface zoomed-in, (d) crystals scratched off from the film, (e) cross-section of the film, and (f) film surface after 10 cycles of electrochromic tests.

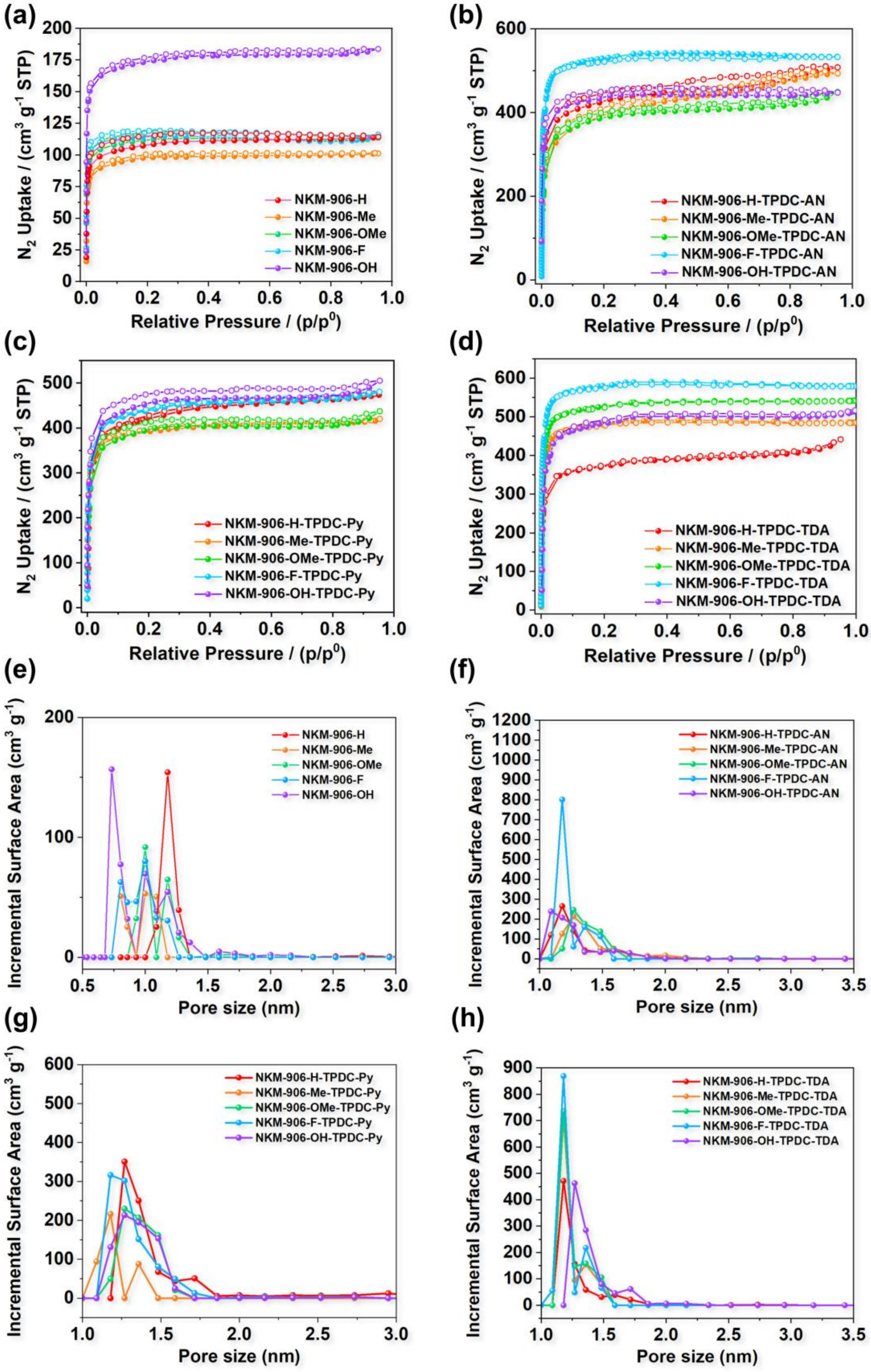

Figure S19. (a-d) Volumetric N_2 adsorption/desorption isotherms and (e-h) the corresponding pore size distributions for NKM-906-R series and NKM-906-R-TPDC-X series.

Table S4. Summary of the calculated Brunauer Emmett Teller (BET) specific surface areas for NKM-906-R series and NKM-906-R-TPDC-X series.

**Compound**	**BET surface area (m^2 g^{-1})**	**Compound**	**BET surface area (m^2 g^{-1})**
NKM-906-H	345	NKM-906-H-TPDC-AN	1394
NKM-906-Me	290	NKM-906-Me-TPDC-AN	1301
NKM-906-OMe	348	NKM-906-OMe-TPDC-AN	1242
NKM-906-F	337	NKM-906-F-TPDC-AN	1887
NKM-906-OH	533	NKM-906-OH-TPDC-AN	1386
NKM-906-H-TPDC-Py	1563	NKM-906-H-TPDC-TDA	1166
NKM-906-Me-TPDC-Py	1294	NKM-906-Me-TPDC-TDA	1637
NKM-906-OMe-TPDC-Py	1242	NKM-906-OMe-TPDC-TDA	1774
NKM-906-F-TPDC-Py	1344	NKM-906-F-TPDC-TDA	1914
NKM-906-OH-TPDC-Py	1417	NKM-906-OH-TPDC-TDA	1643

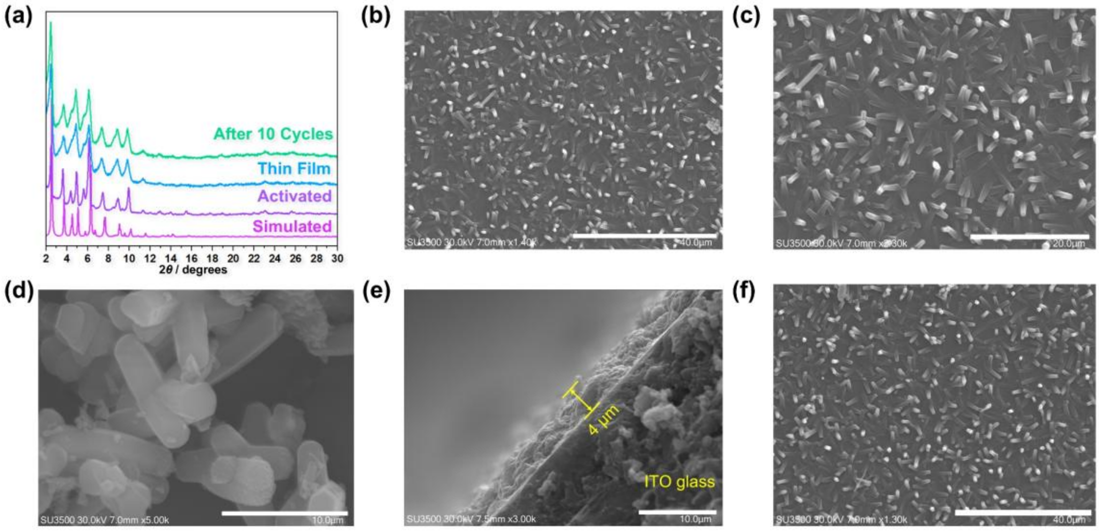

Figure S20. General characterizations of NKM-908-H-TPDC-AN showing (a) PXRD patterns of the simulated (magenta), activated crystals (purple), thin film (cyan) and thin film after 10 cycles of electrochromic tests (green); and SEM images of (b) film surface, (c) film surface zoomed-in, (d) crystals scratched off from the film, (e) cross-section of the film, and (f) film surface after 10 cycles of electrochromic tests.

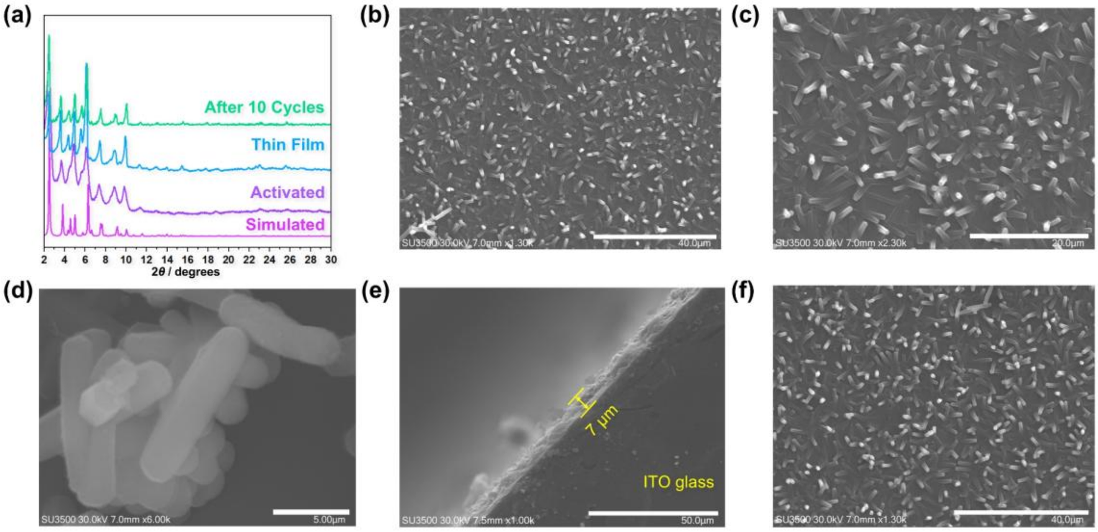

Figure S21. General characterizations of NKM-908-Me-TPDC-AN showing (a) PXRD patterns of the simulated (magenta), activated crystals (purple), thin film (cyan) and thin film after 10 cycles of electrochromic tests (green); and SEM images of (b) film surface, (c) film surface zoomed-in, (d) crystals scratched off from the film, (e) cross-section of the film, and (f) film surface after 10 cycles of electrochromic tests.

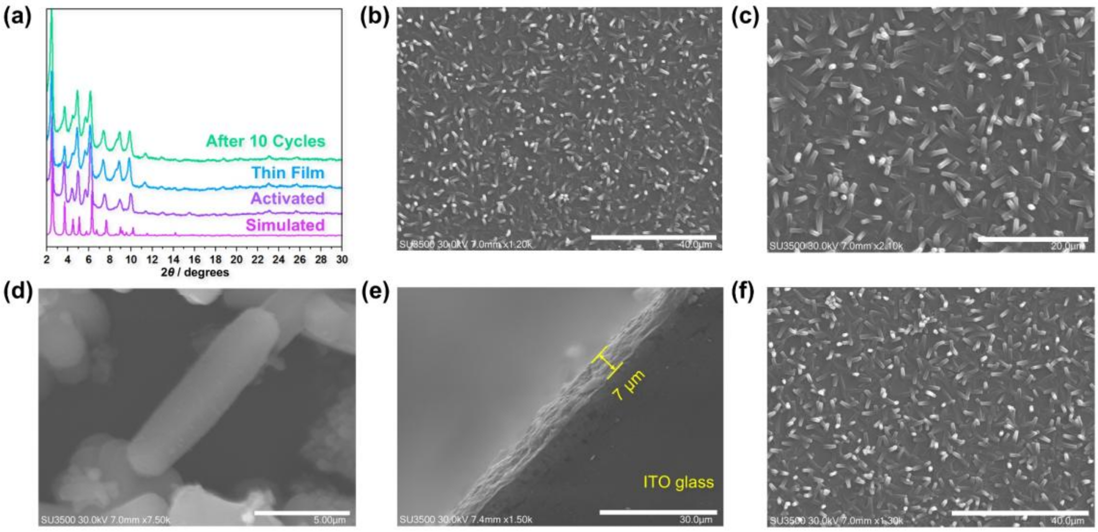

Figure S22. General characterizations of NKM-908-OMe-TPDC-AN showing (a) PXRD patterns of the simulated (magenta), activated crystals (purple), thin film (cyan) and thin film after 10 cycles of electrochromic tests (green); and SEM images of (b) film surface, (c) film surface zoomed-in, (d) crystals scratched off from the film, (e) cross-section of the film, and (f) film surface after 10 cycles of electrochromic tests.

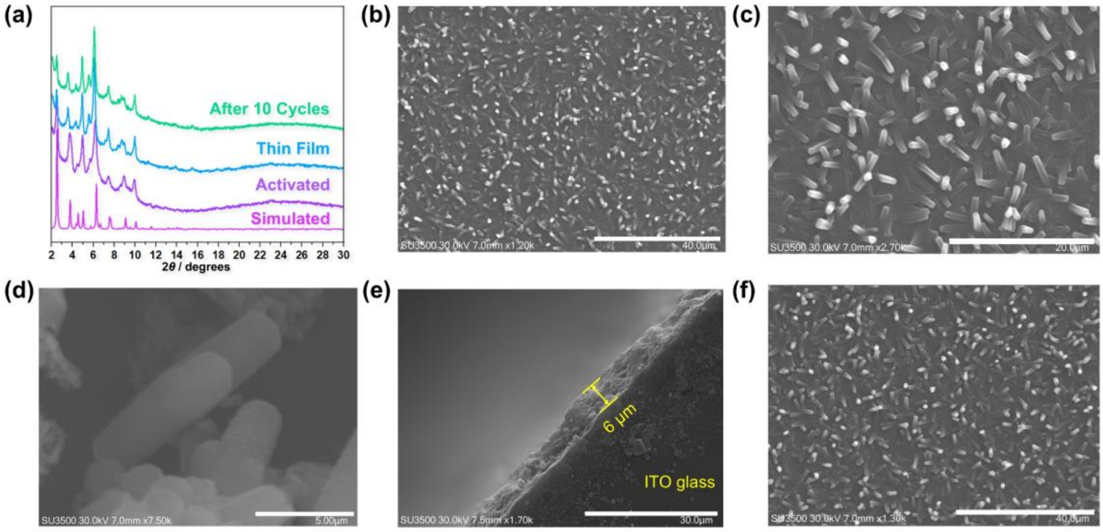

Figure S23. General characterizations of NKM-908-F-TPDC-AN showing (a) PXRD patterns of the simulated (magenta), activated crystals (purple), thin film (cyan) and thin film after 10 cycles of electrochromic tests (green); and SEM images of (b) film surface, (c) film surface zoomed-in, (d) crystals scratched off from the film, (e) cross-section of the film, and (f) film surface after 10 cycles of electrochromic tests.

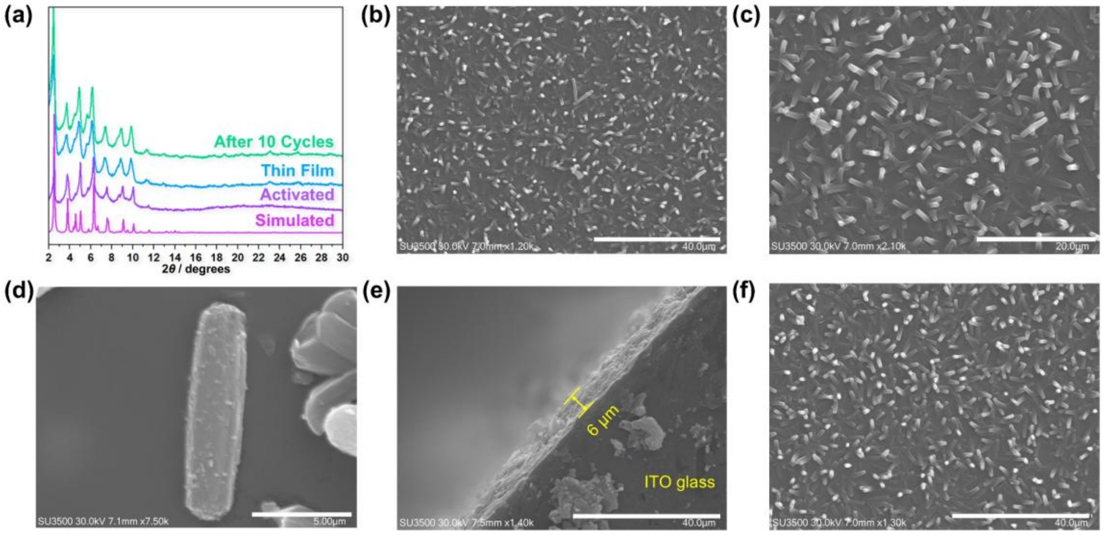

Figure S24. General characterizations of NKM-908-OH-TPDC-AN showing (a) PXRD patterns of the simulated (magenta), activated crystals (purple), thin film (cyan) and thin film after 10 cycles of electrochromic tests (green); and SEM images of (b) film surface, (c) film surface zoomed-in, (d) crystals scratched off from the film, (e) cross-section of the film, and (f) film surface after 10 cycles of electrochromic tests.

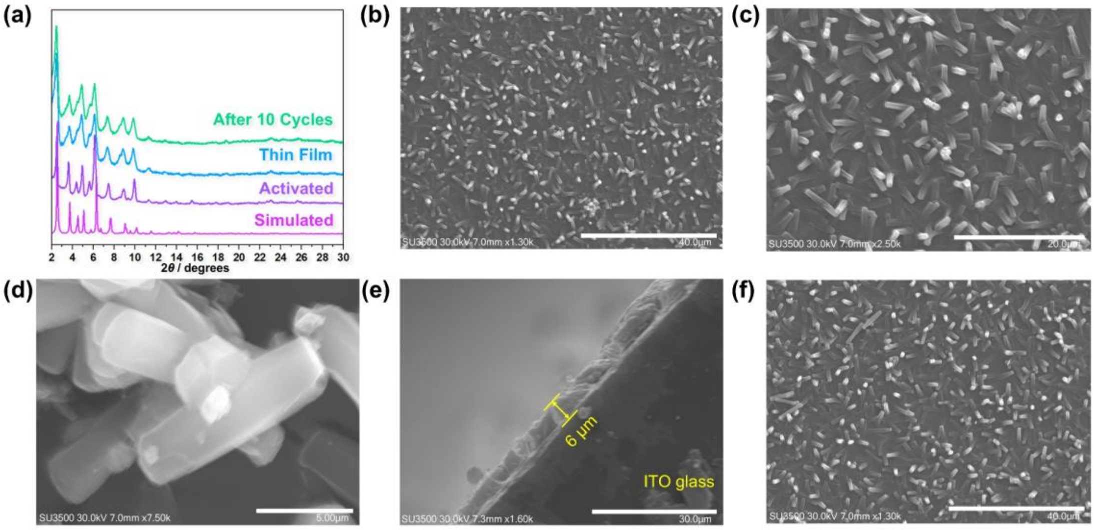

Figure S25. General characterizations of NKM-908-H-TPDC-Py showing (a) PXRD patterns of the simulated (magenta), activated crystals (purple), thin film (cyan) and thin film after 10 cycles of electrochromic tests (green); and SEM images of (b) film surface, (c) film surface zoomed-in, (d) crystals scratched off from the film, (e) cross-section of the film, and (f) film surface after 10 cycles of electrochromic tests.

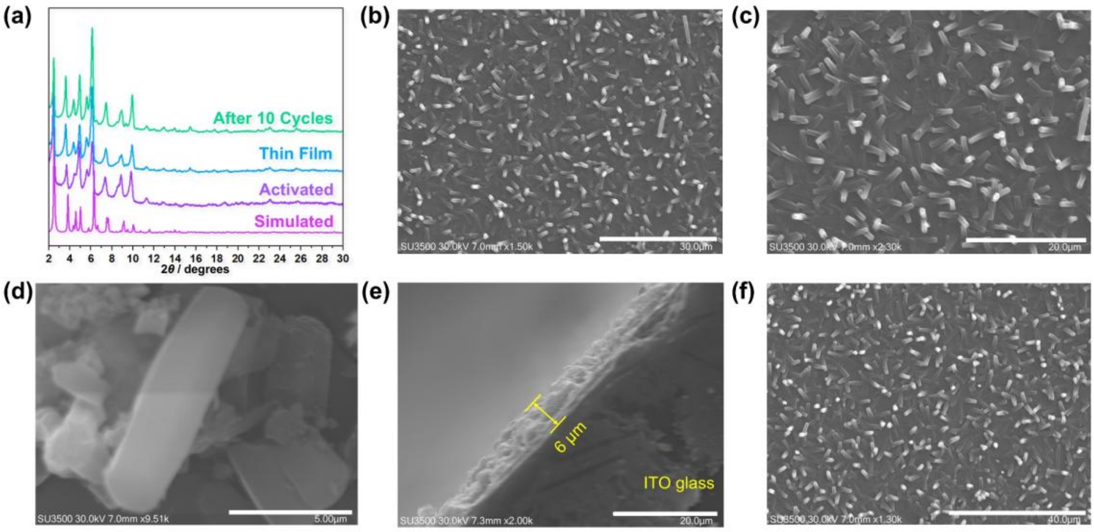

Figure S26. General characterizations of NKM-908-Me-TPDC-Py showing (a) PXRD patterns of the simulated (magenta), activated crystals (purple), thin film (cyan) and thin film after 10 cycles of electrochromic tests (green); and SEM images of (b) film surface, (c) film surface zoomed-in, (d) crystals scratched off from the film, (e) cross-section of the film, and (f) film surface after 10 cycles of electrochromic tests.

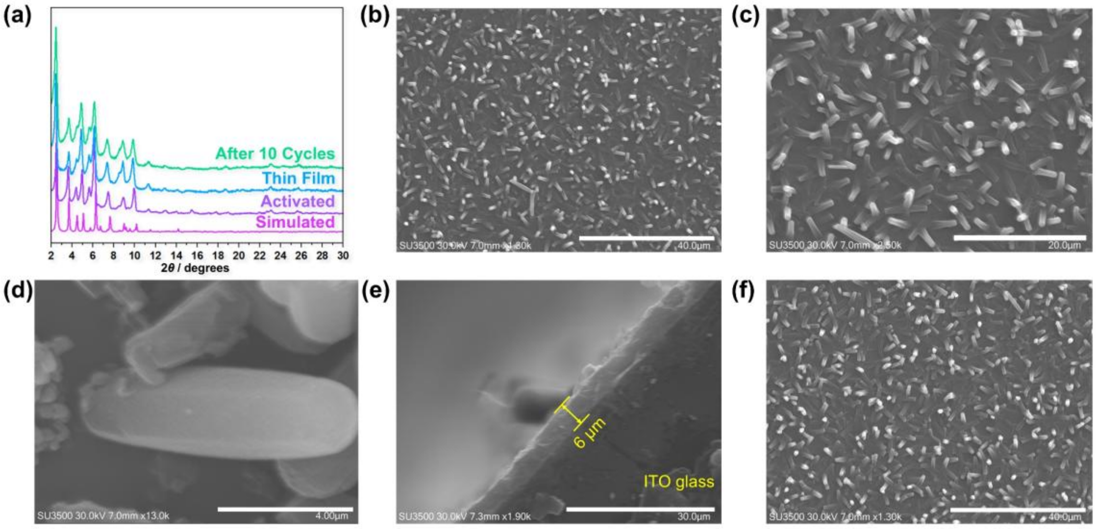

Figure S27. General characterizations of NKM-908-OMe-TPDC-Py showing (a) PXRD patterns of the simulated (magenta), activated crystals (purple), thin film (cyan) and thin film after 10 cycles of electrochromic tests (green); and SEM images of (b) film surface, (c) film surface zoomed-in, (d) crystals scratched off from the film, (e) cross-section of the film, and (f) film surface after 10 cycles of electrochromic tests.

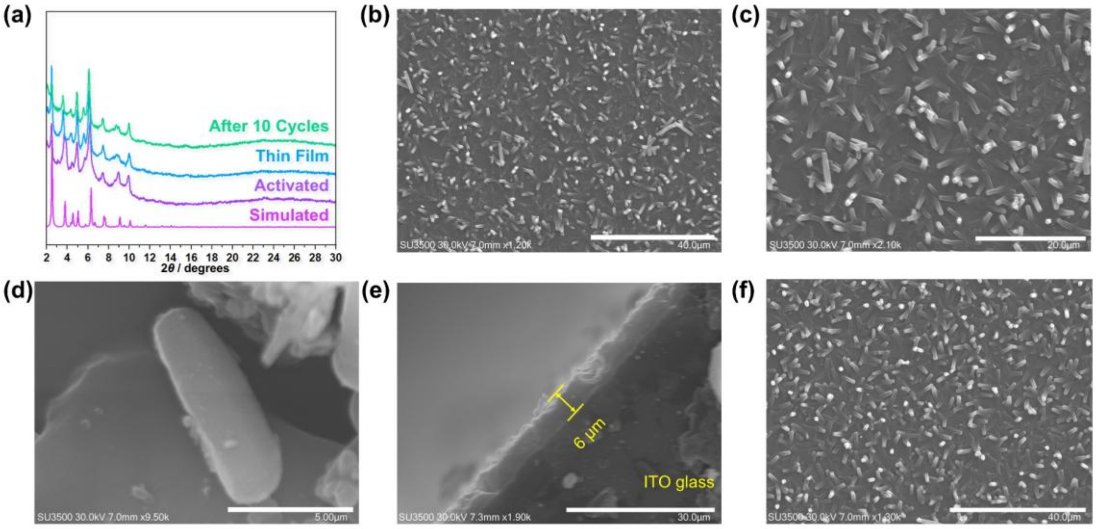

Figure S28. General characterizations of NKM-908-F-TPDC-Py showing (a) PXRD patterns of the simulated (magenta), activated crystals (purple), thin film (cyan) and thin film after 10 cycles of electrochromic tests (green); and SEM images of (b) film surface, (c) film surface zoomed-in, (d) crystals scratched off from the film, (e) cross-section of the film, and (f) film surface after 10 cycles of electrochromic tests.

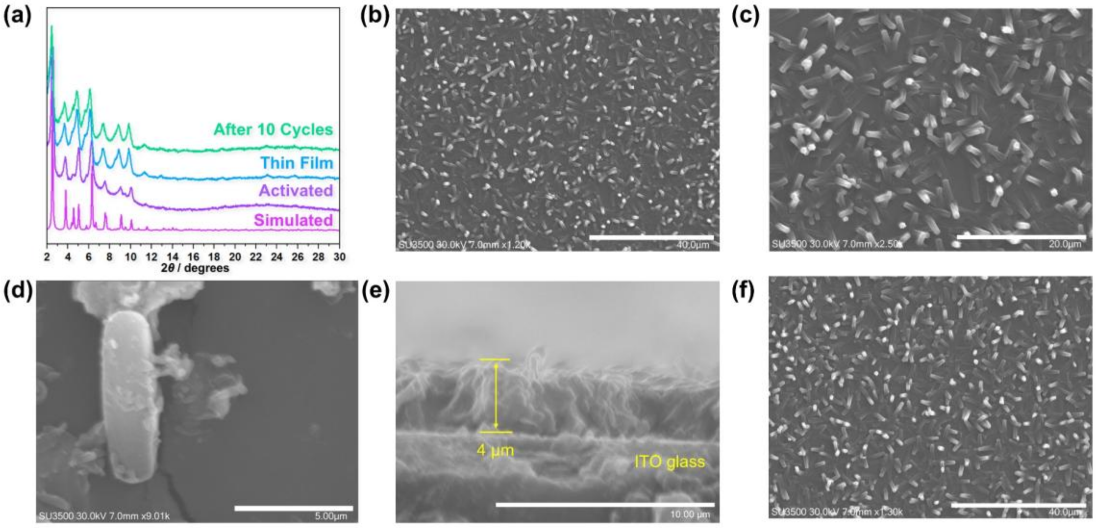

Figure S29. General characterizations of NKM-908-OH-TPDC-Py showing (a) PXRD patterns of the simulated (magenta), activated crystals (purple), thin film (cyan) and thin film after 10 cycles of electrochromic tests (green); and SEM images of (b) film surface, (c) film surface zoomed-in, (d) crystals scratched off from the film, (e) cross-section of the film, and (f) film surface after 10 cycles of electrochromic tests.

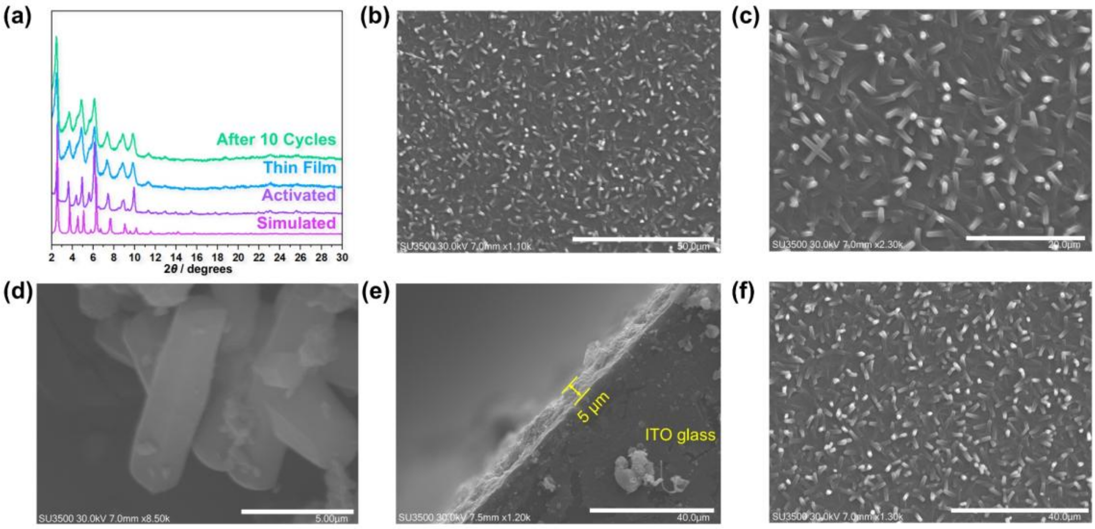

Figure S30. General characterizations of NKM-908-H-TPDC-TDA showing (a) PXRD patterns of the simulated (magenta), activated crystals (purple), thin film (cyan) and thin film after 10 cycles of electrochromic tests (green); and SEM images of (b) film surface, (c) film surface zoomed-in, (d) crystals scratched off from the film, (e) cross-section of the film, and (f) film surface after 10 cycles of electrochromic tests.

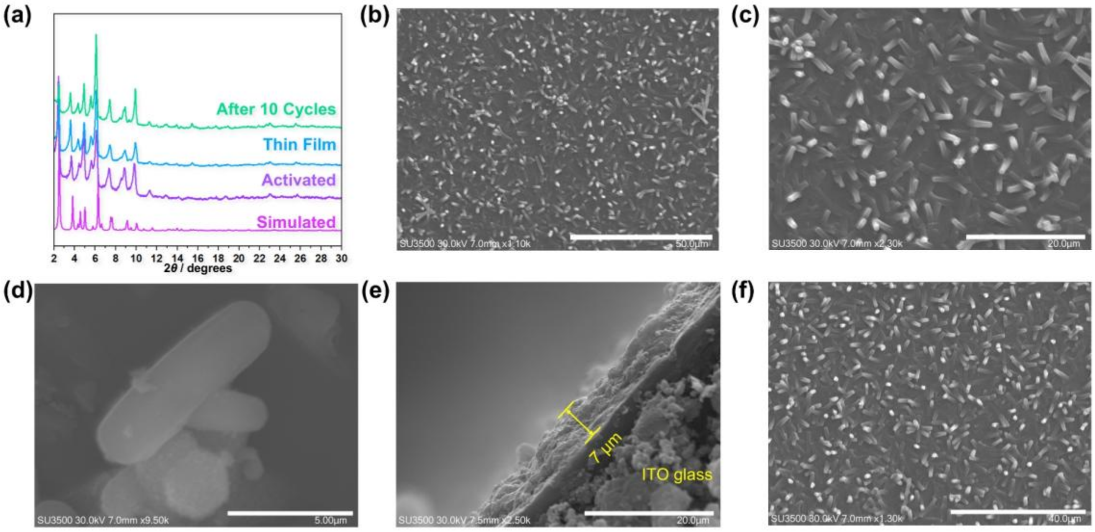

Figure S31. General characterizations of NKM-908-Me-TPDC-TDA showing (a) PXRD patterns of the simulated (magenta), activated crystals (purple), thin film (cyan) and thin film after 10 cycles of electrochromic tests (green); and SEM images of (b) film surface, (c) film surface zoomed-in, (d) crystals scratched off from the film, (e) cross-section of the film, and (f) film surface after 10 cycles of electrochromic tests.

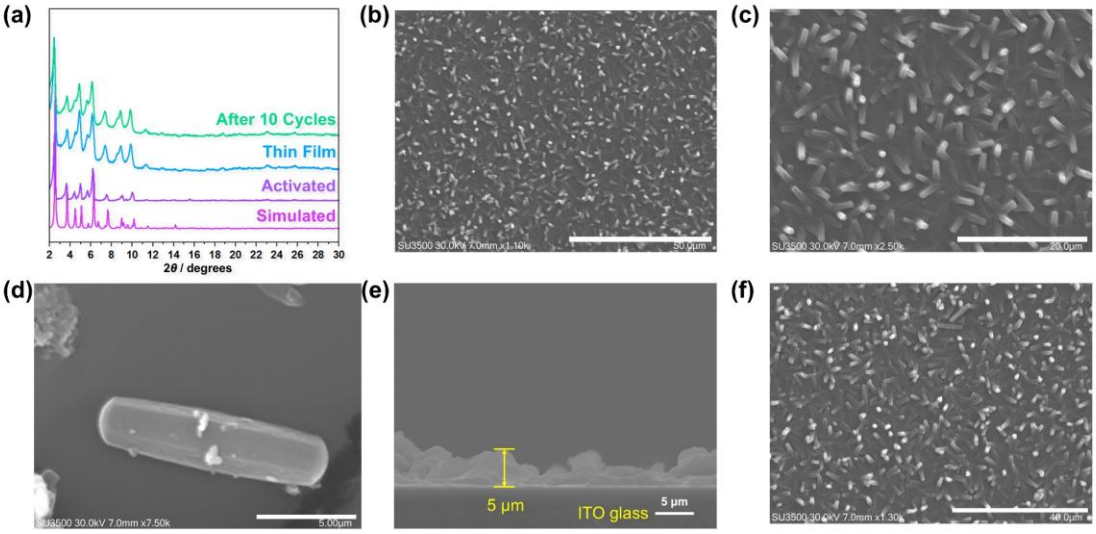

Figure S32. General characterizations of NKM-908-OMe-TPDC-TDA showing (a) PXRD patterns of the simulated (magenta), activated crystals (purple), thin film (cyan) and thin film after 10 cycles of electrochromic tests (green); and SEM images of (b) film surface, (c) film surface zoomed-in, (d) crystals scratched off from the film, (e) cross-section of the film, and (f) film surface after 10 cycles of electrochromic tests.

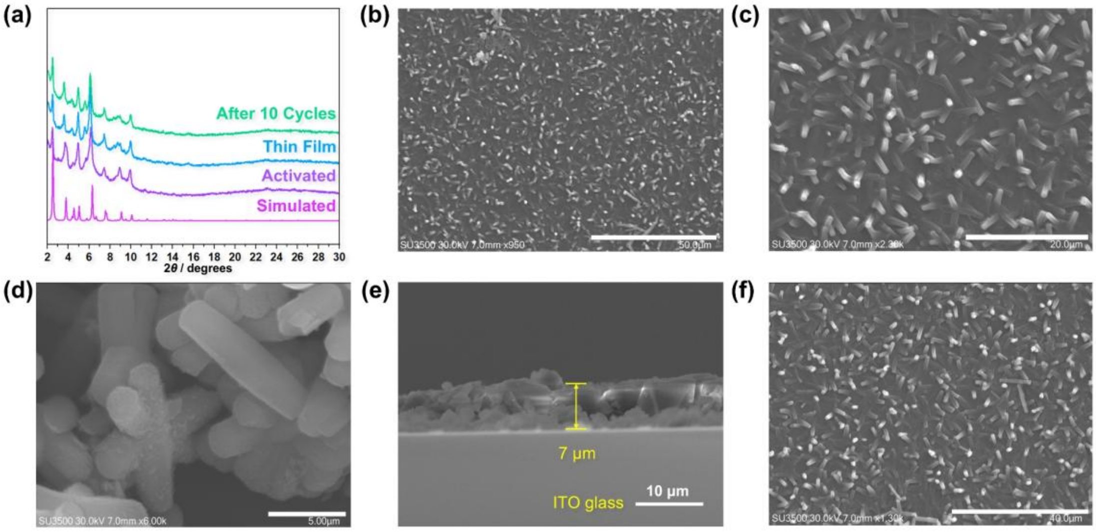

Figure S33. General characterizations of NKM-908-F-TPDC-TDA showing (a) PXRD patterns of the simulated (magenta), activated crystals (purple), thin film (cyan) and thin film after 10 cycles of electrochromic tests (green); and SEM images of (b) film surface, (c) film surface zoomed-in, (d) crystals scratched off from the film, (e) cross-section of the film, and (f) film surface after 10 cycles of electrochromic tests.

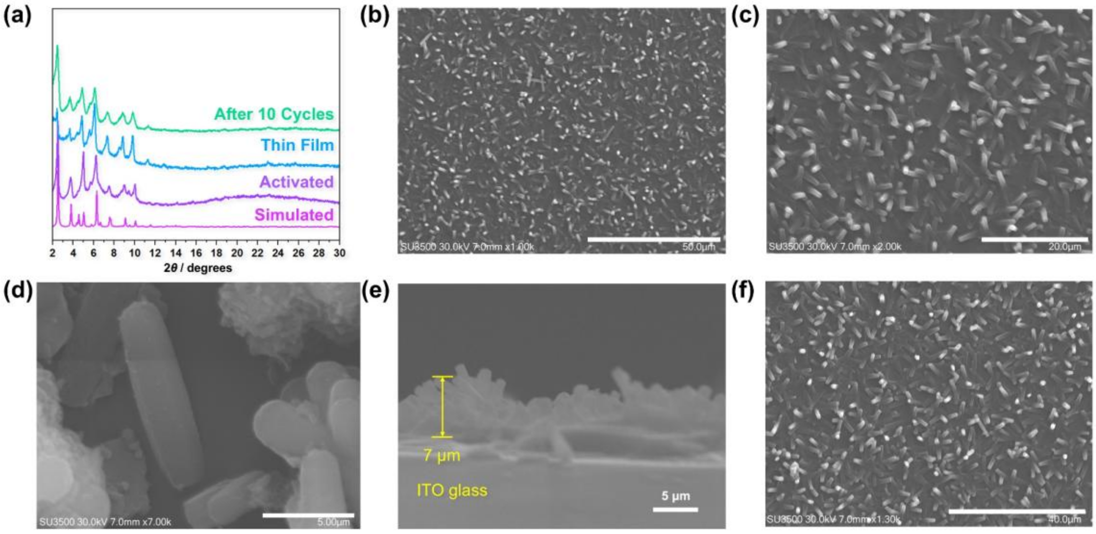

Figure S34. General characterizations of NKM-908-OH-TPDC-TDA showing (a) PXRD patterns of the simulated (magenta), activated crystals (purple), thin film (cyan) and thin film after 10 cycles of electrochromic tests (green); and SEM images of (b) film surface, (c) film surface zoomed-in, (d) crystals scratched off from the film, (e) cross-section of the film, and (f) film surface after 10 cycles of electrochromic tests.

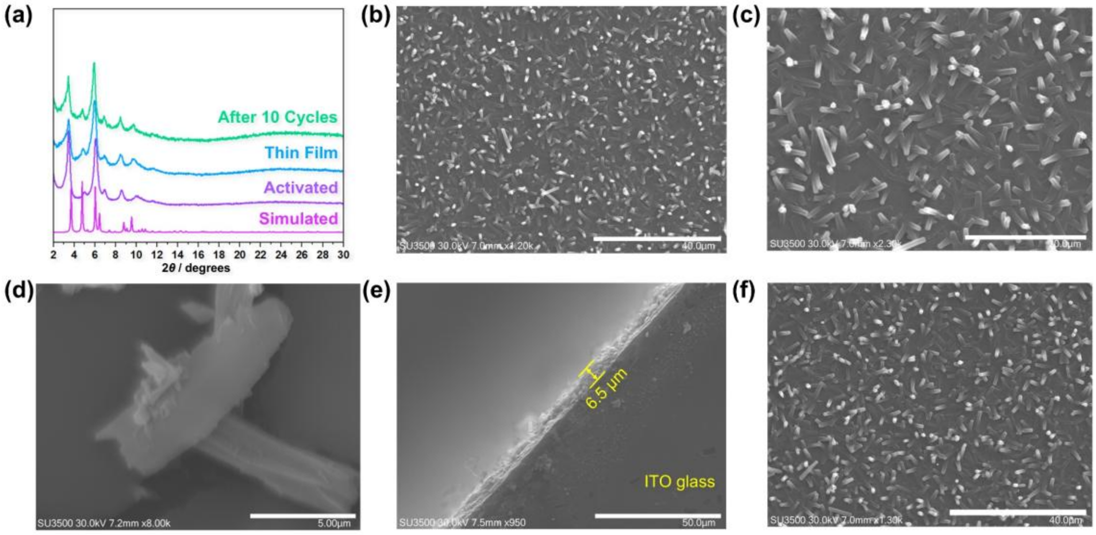

Figure S35. General characterizations of NKM-906-H-TPDC-AN showing (a) PXRD patterns of the simulated (magenta), activated crystals (purple), thin film (cyan) and thin film after 10 cycles of electrochromic tests (green); and SEM images of (b) film surface, (c) film surface zoomed-in, (d) crystals scratched off from the film, (e) cross-section of the film, and (f) film surface after 10 cycles of electrochromic tests.

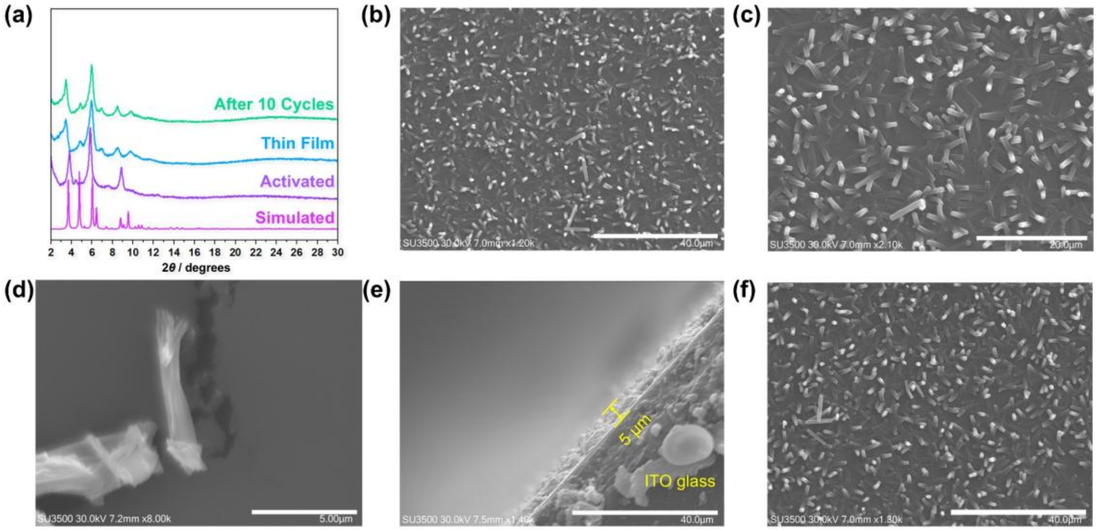

Figure S36. General characterizations of NKM-906-Me-TPDC-AN showing (a) PXRD patterns of the simulated (magenta), activated crystals (purple), thin film (cyan) and thin film after 10 cycles of electrochromic tests (green); and SEM images of (b) film surface, (c) film surface zoomed-in, (d) crystals scratched off from the film, (e) cross-section of the film, and (f) film surface after 10 cycles of electrochromic tests.

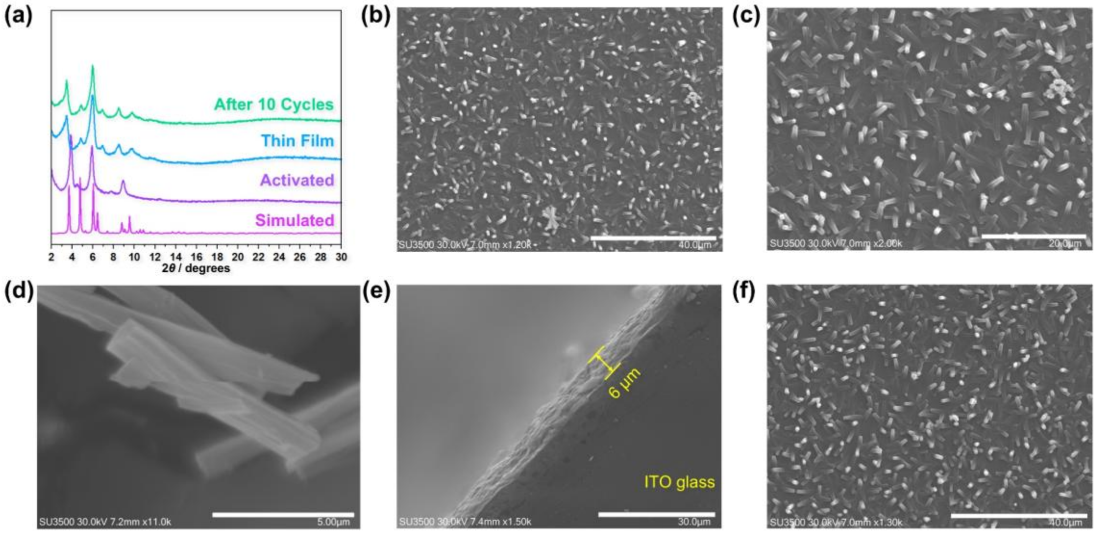

Figure S37. General characterizations of NKM-906-OMe-TPDC-AN showing (a) PXRD patterns of the simulated (magenta), activated crystals (purple), thin film (cyan) and thin film after 10 cycles of electrochromic tests (green); and SEM images of (b) film surface, (c) film surface zoomed-in, (d) crystals scratched off from the film, (e) cross-section of the film, and (f) film surface after 10 cycles of electrochromic tests.

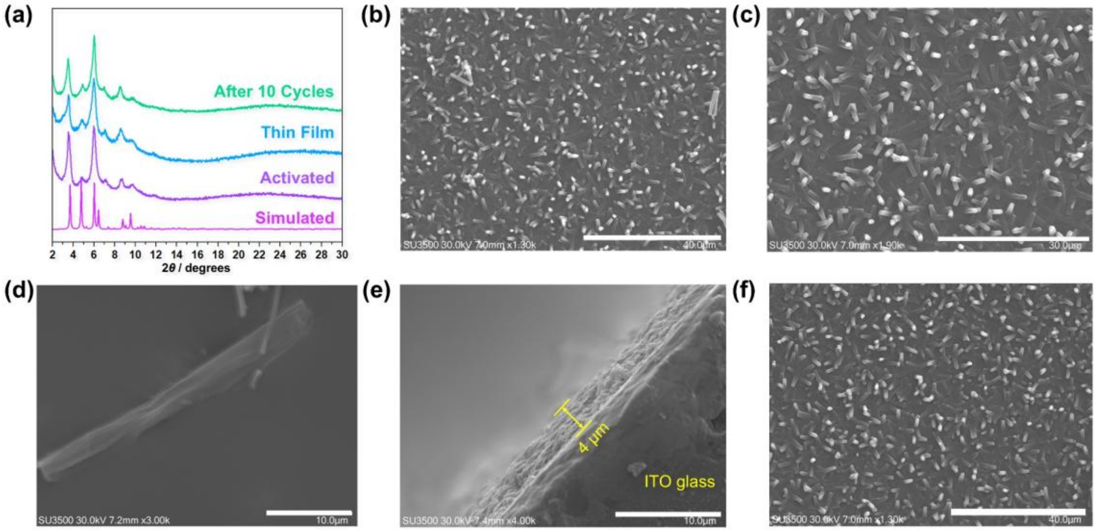

Figure S38. General characterizations of NKM-906-F-TPDC-AN showing (a) PXRD patterns of the simulated (magenta), activated crystals (purple), thin film (cyan) and thin film after 10 cycles of electrochromic tests (green); and SEM images of (b) film surface, (c) film surface zoomed-in, (d) crystals scratched off from the film, (e) cross-section of the film, and (f) film surface after 10 cycles of electrochromic tests.

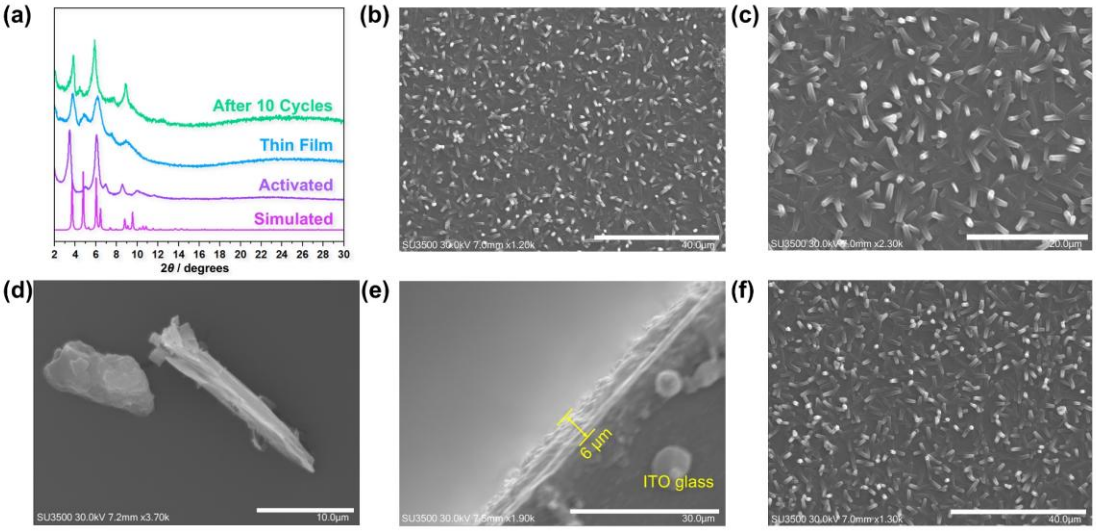

Figure S39. General characterizations of NKM-906-OH-TPDC-AN showing (a) PXRD patterns of the simulated (magenta), activated crystals (purple), thin film (cyan) and thin film after 10 cycles of electrochromic tests (green); and SEM images of (b) film surface, (c) film surface zoomed-in, (d) crystals scratched off from the film, (e) cross-section of the film, and (f) film surface after 10 cycles of electrochromic tests.

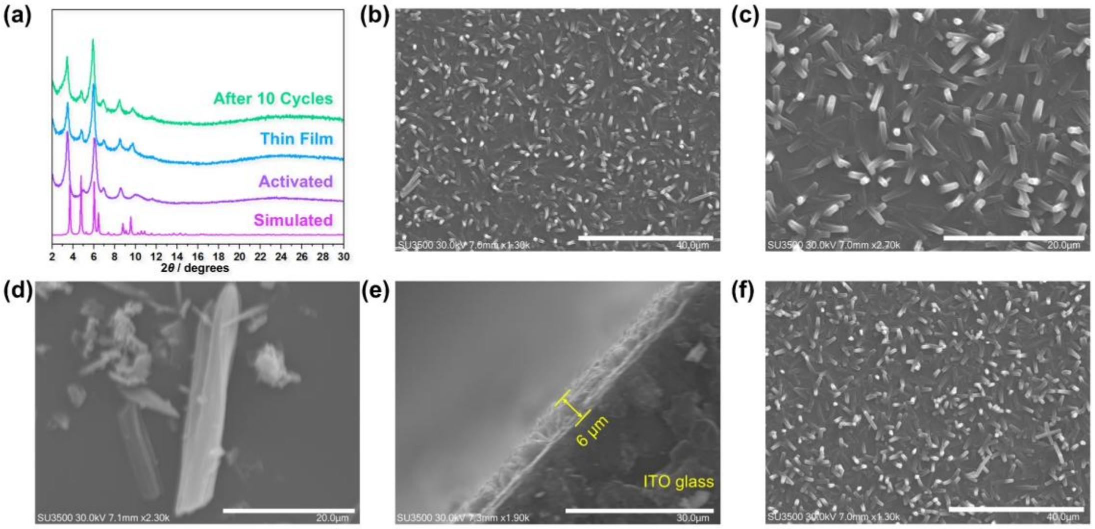

Figure S40. General characterizations of NKM-906-H-TPDC-Py showing (a) PXRD patterns of the simulated (magenta), activated crystals (purple), thin film (cyan) and thin film after 10 cycles of electrochromic tests (green); and SEM images of (b) film surface, (c) film surface zoomed-in, (d) crystals scratched off from the film, (e) cross-section of the film, and (f) film surface after 10 cycles of electrochromic tests.

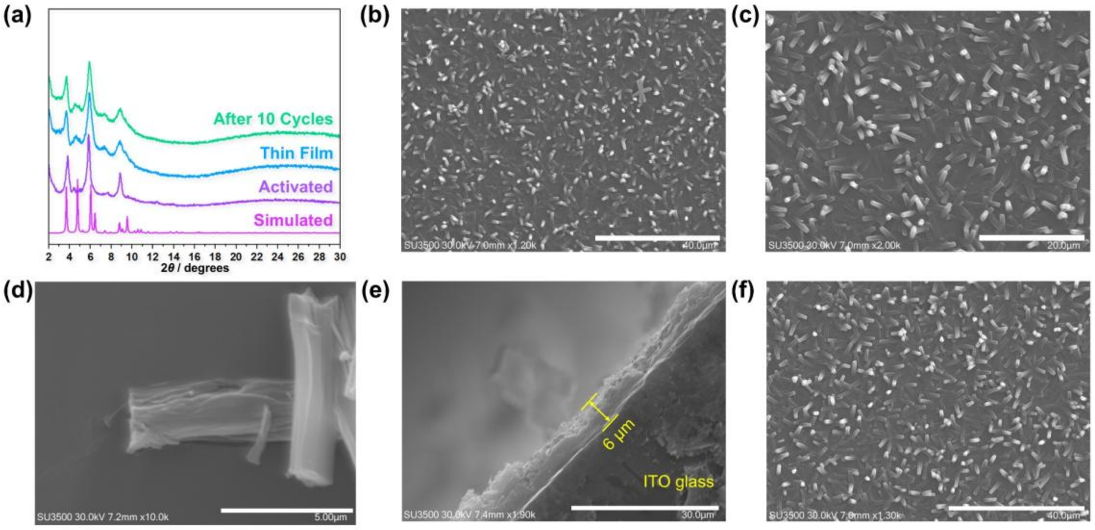

Figure S41. General characterizations of NKM-906-Me-TPDC-Py showing (a) PXRD patterns of the simulated (magenta), activated crystals (purple), thin film (cyan) and thin film after 10 cycles of electrochromic tests (green); and SEM images of (b) film surface, (c) film surface zoomed-in, (d) crystals scratched off from the film, (e) cross-section of the film, and (f) film surface after 10 cycles of electrochromic tests.

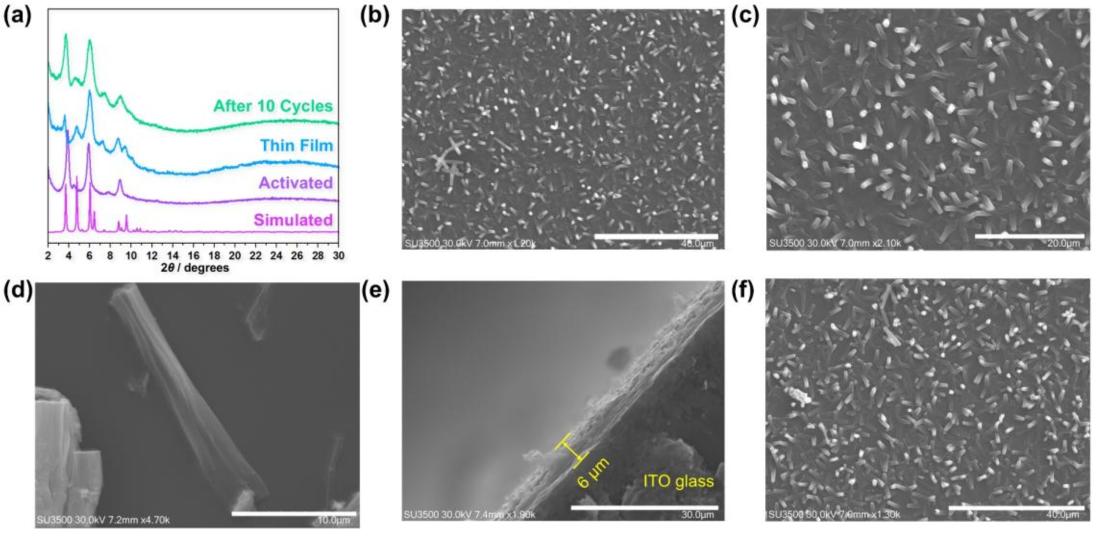

Figure S42. General characterizations of NKM-906-OMe-TPDC-Py showing (a) PXRD patterns of the simulated (magenta), activated crystals (purple), thin film (cyan) and thin film after 10 cycles of electrochromic tests (green); and SEM images of (b) film surface, (c) film surface zoomed-in, (d) crystals scratched off from the film, (e) cross-section of the film, and (f) film surface after 10 cycles of electrochromic tests.

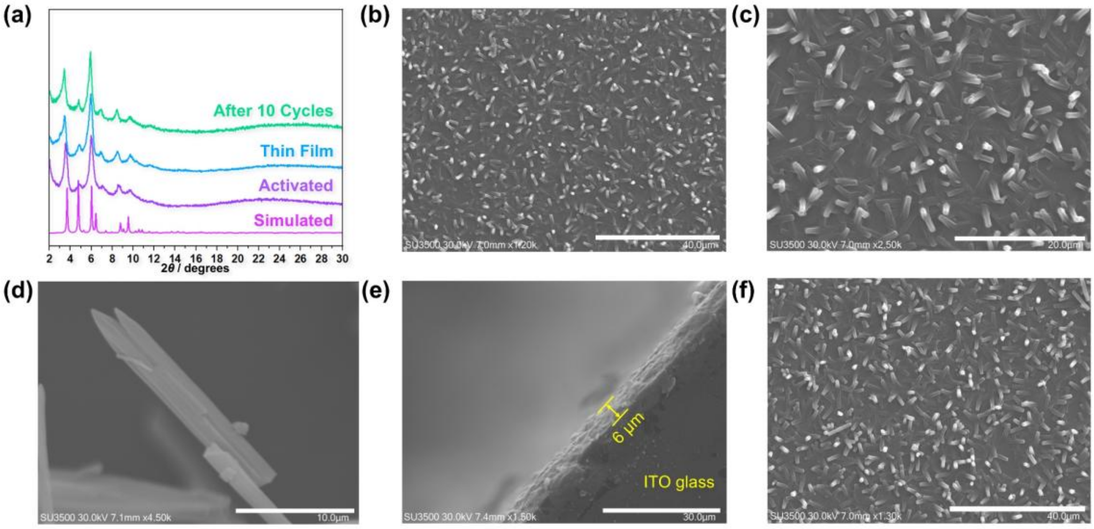

Figure S43. General characterizations of NKM-906-F-TPDC-Py showing (a) PXRD patterns of the simulated (magenta), activated crystals (purple), thin film (cyan) and thin film after 10 cycles of electrochromic tests (green); and SEM images of (b) film surface, (c) film surface zoomed-in, (d) crystals scratched off from the film, (e) cross-section of the film, and (f) film surface after 10 cycles of electrochromic tests.

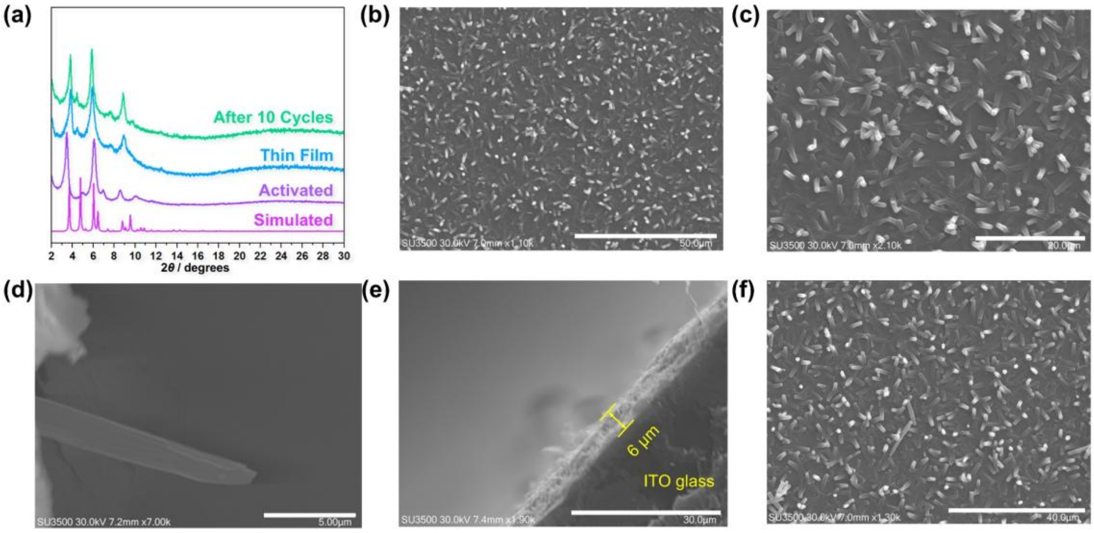

Figure S44. General characterizations of NKM-906-OH-TPDC-Py showing (a) PXRD patterns of the simulated (magenta), activated crystals (purple), thin film (cyan) and thin film after 10 cycles of electrochromic tests (green); and SEM images of (b) film surface, (c) film surface zoomed-in, (d) crystals scratched off from the film, (e) cross-section of the film, and (f) film surface after 10 cycles of electrochromic tests.

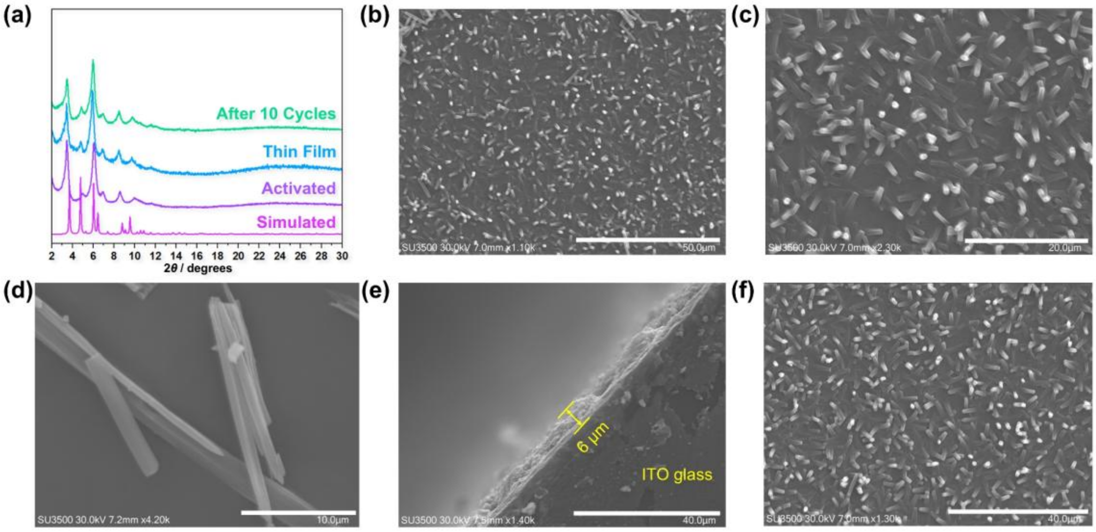

Figure S45. General characterizations of NKM-906-H-TPDC-TDA showing (a) PXRD patterns of the simulated (magenta), activated crystals (purple), thin film (cyan) and thin film after 10 cycles of electrochromic tests (green); and SEM images of (b) film surface, (c) film surface zoomed-in, (d) crystals scratched off from the film, (e) cross-section of the film, and (f) film surface after 10 cycles of electrochromic tests.

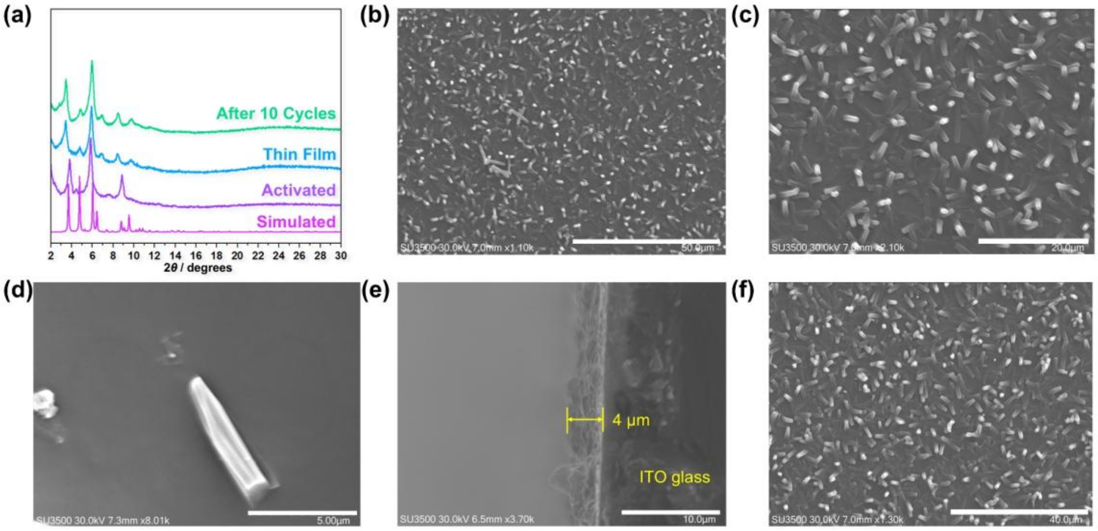

Figure S46. General characterizations of NKM-906-Me-TPDC-TDA showing (a) PXRD patterns of the simulated (magenta), activated crystals (purple), thin film (cyan) and thin film after 10 cycles of electrochromic tests (green); and SEM images of (b) film surface, (c) film surface zoomed-in, (d) crystals scratched off from the film, (e) cross-section of the film, and (f) film surface after 10 cycles of electrochromic tests.

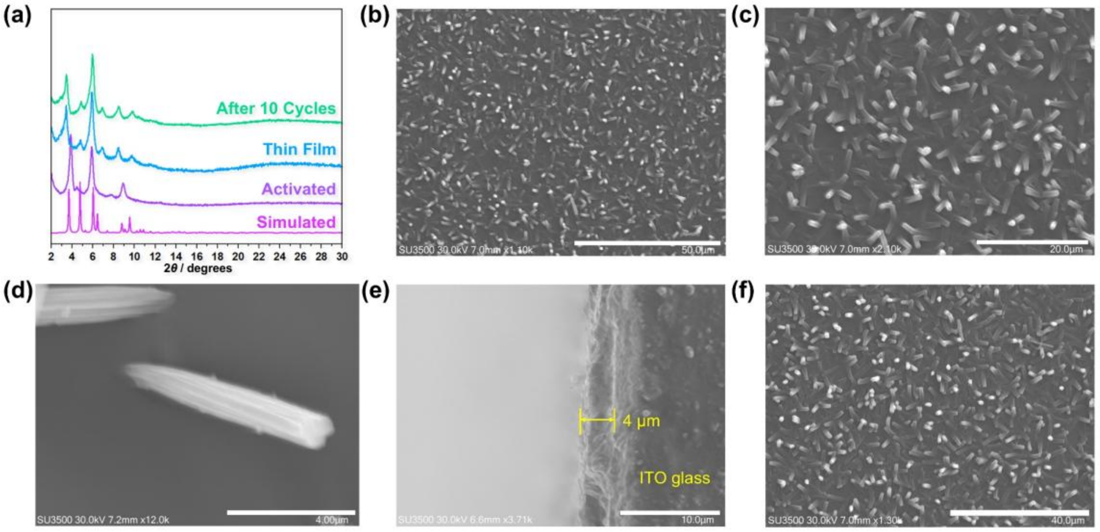

Figure S47. General characterizations of NKM-906-OMe-TPDC-TDA showing (a) PXRD patterns of the simulated (magenta), activated crystals (purple), thin film (cyan) and thin film after 10 cycles of electrochromic tests (green); and SEM images of (b) film surface, (c) film surface zoomed-in, (d) crystals scratched off from the film, (e) cross-section of the film, and (f) film surface after 10 cycles of electrochromic tests.

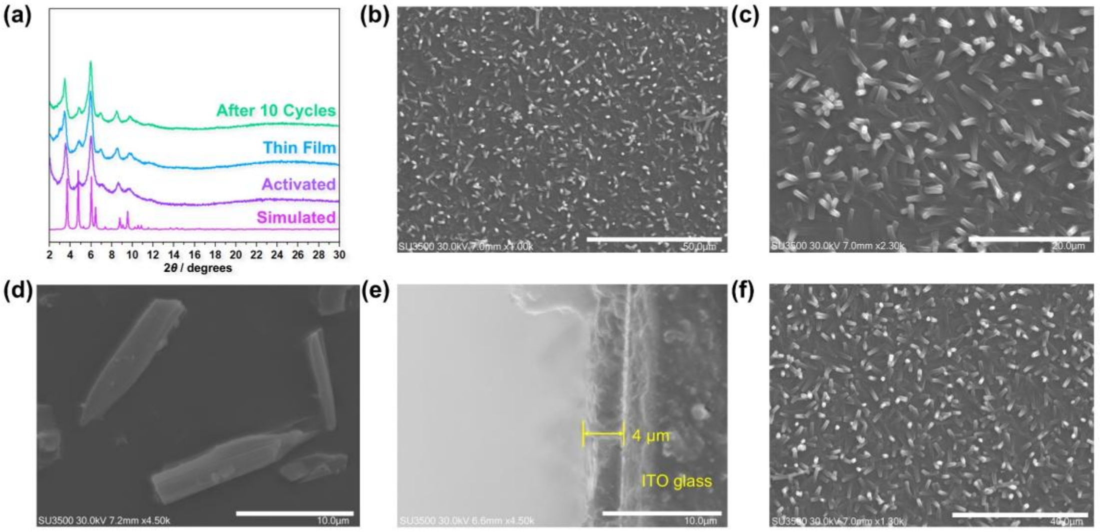

Figure S48. General characterizations of NKM-906-F-TPDC-TDA showing (a) PXRD patterns of the simulated (magenta), activated crystals (purple), thin film (cyan) and thin film after 10 cycles of electrochromic tests (green); and SEM images of (b) film surface, (c) film surface zoomed-in, (d) crystals scratched off from the film, (e) cross-section of the film, and (f) film surface after 10 cycles of electrochromic tests.

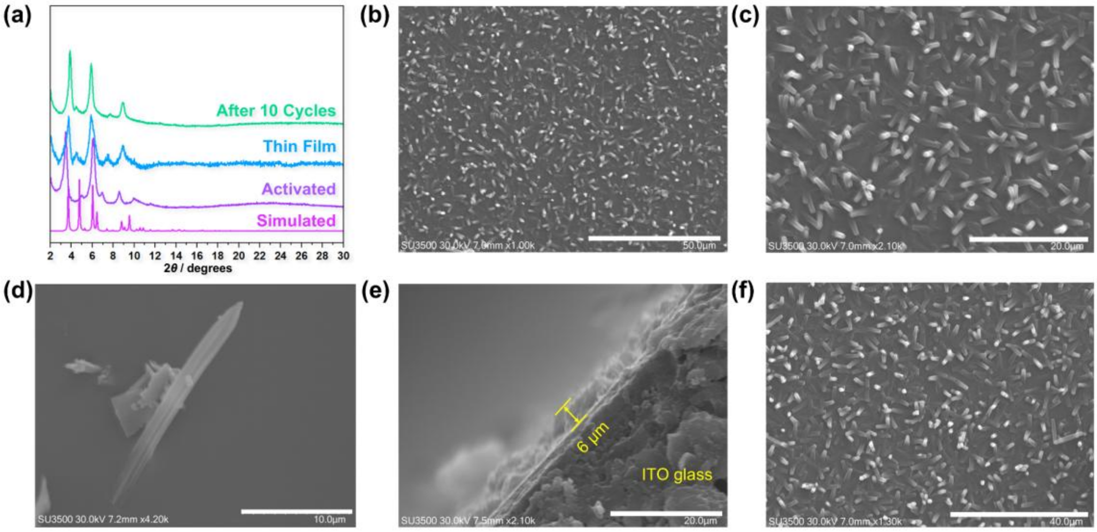

Figure S49. General characterizations of NKM-906-OH-TPDC-TDA showing (a) PXRD patterns of the simulated (magenta), activated crystals (purple), thin film (cyan) and thin film after 10 cycles of electrochromic tests (green); and SEM images of (b) film surface, (c) film surface zoomed-in, (d) crystals scratched off from the film, (e) cross-section of the film, and (f) film surface after 10 cycles of electrochromic tests.

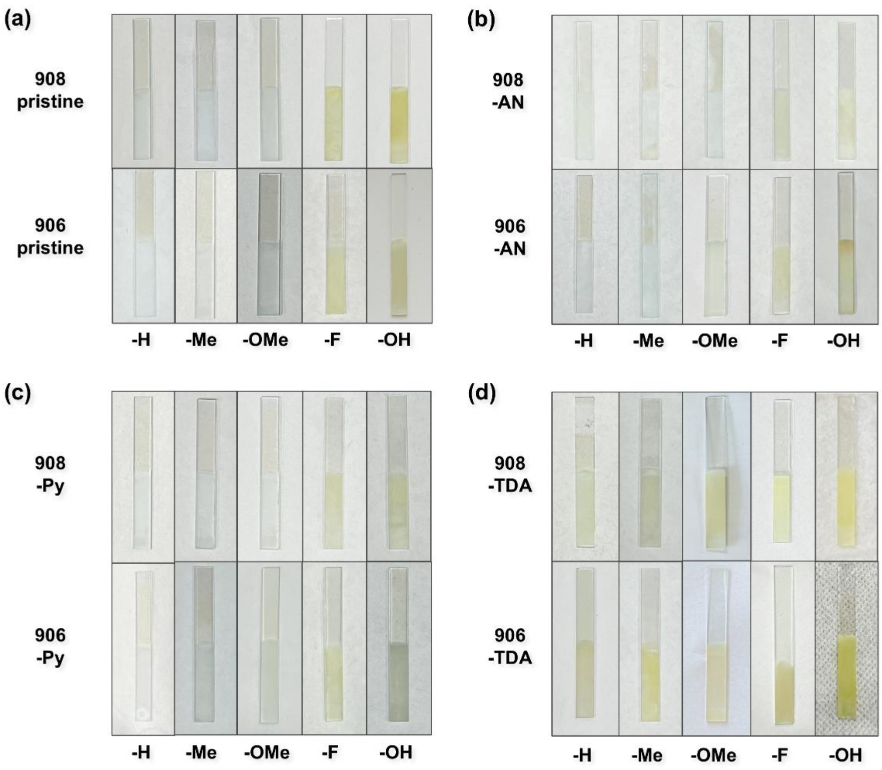

Figure S50. Optical photographs for all forty as-synthesized MOF thin films (dry) on ITO glass.

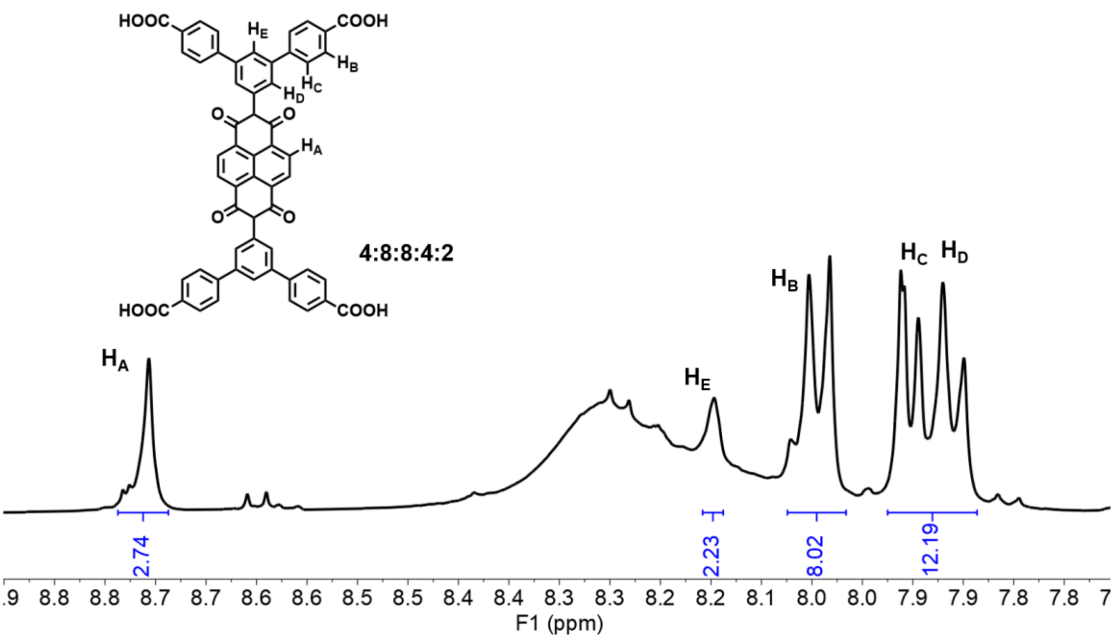

Figure S51. ^{1}H NMR (400 MHz, DMSO-d_6) spectrum of digested NKM-908-H.

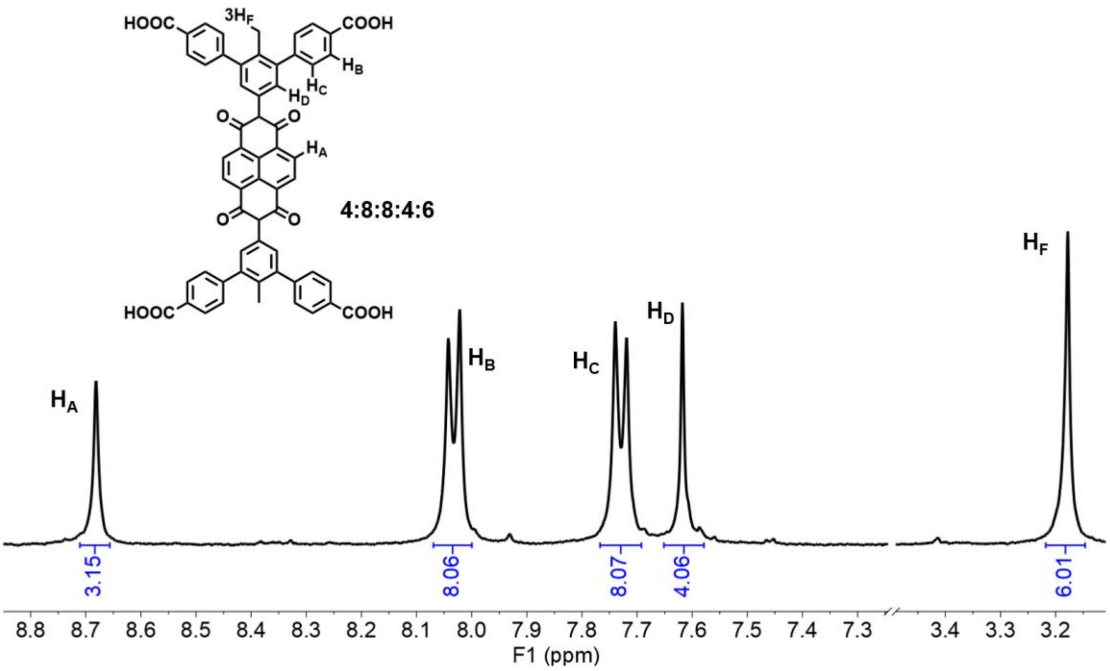

Figure S52. ^{1}H NMR (400 MHz, DMSO-d_6) spectrum of digested NKM-908-Me.

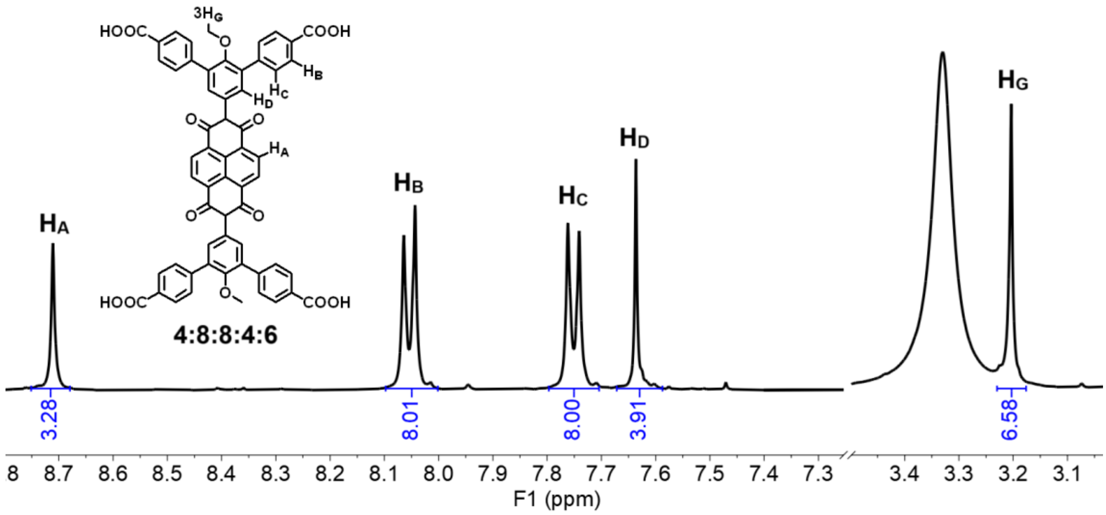

Figure S53. ^{1}H NMR (400 MHz, DMSO-d_6) spectrum of digested NKM-908-OMe.

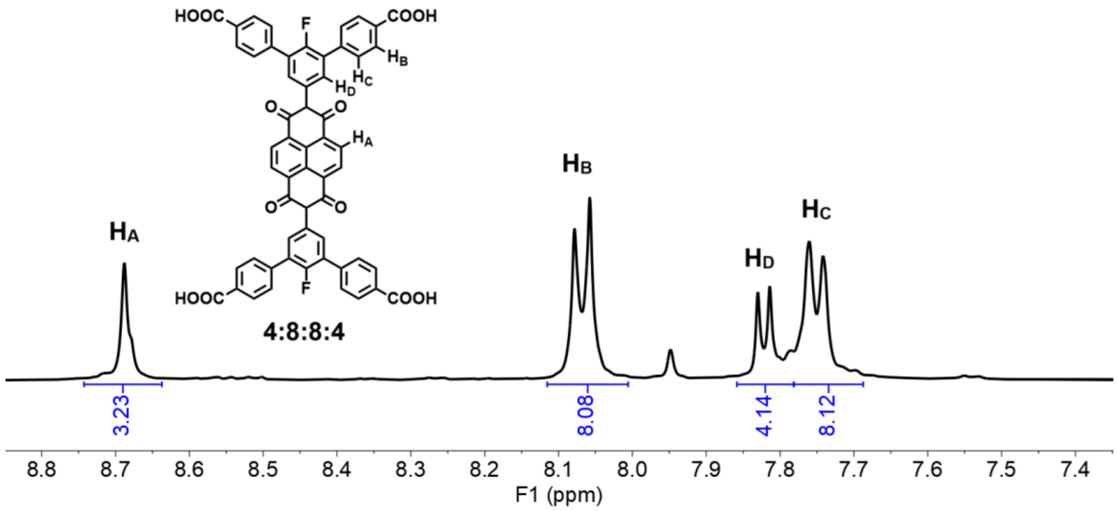

Figure S54. ^{1}H NMR (400 MHz, DMSO-d_6) spectrum of digested NKM-908-F.

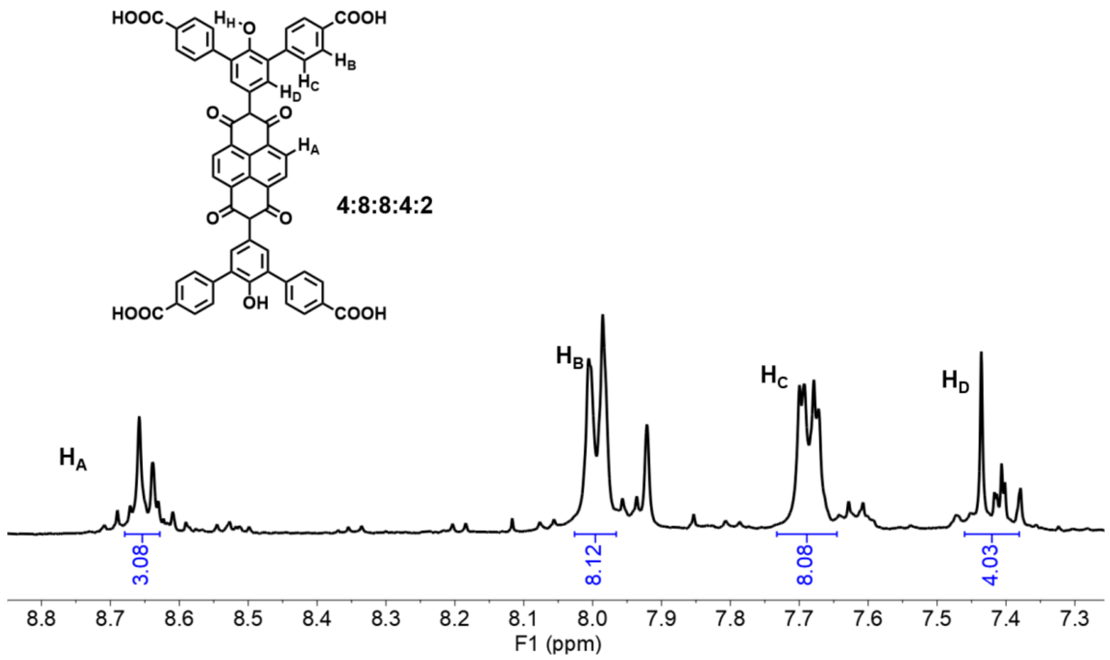

Figure S55. ^{1}H NMR (400 MHz, DMSO-d_6) spectrum of digested NKM-908-OH.

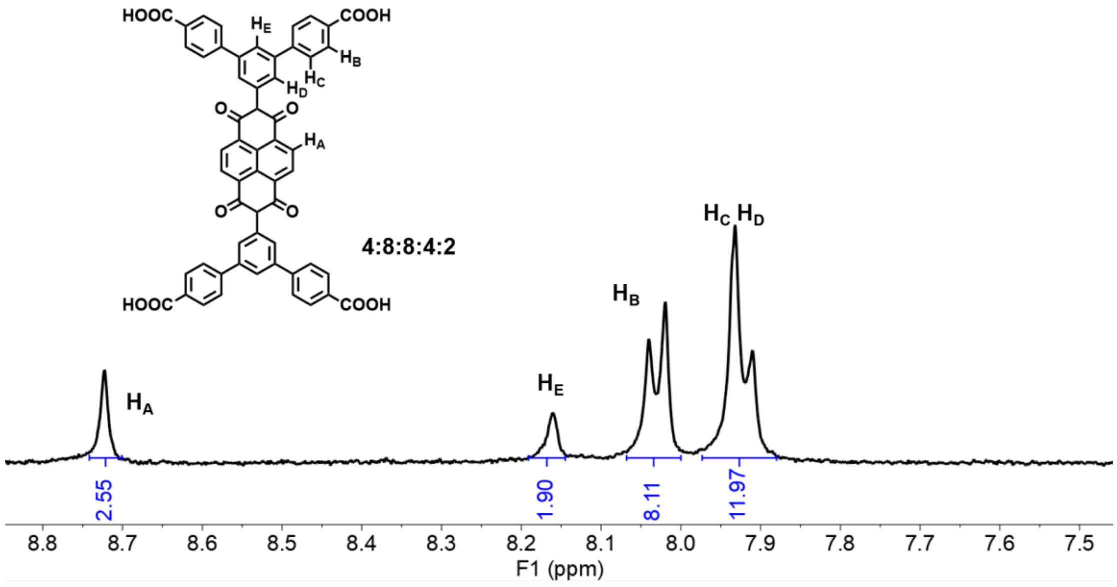

Figure S56. ^{1}H NMR (400 MHz, DMSO-d_6) spectrum of digested NKM-906-H.

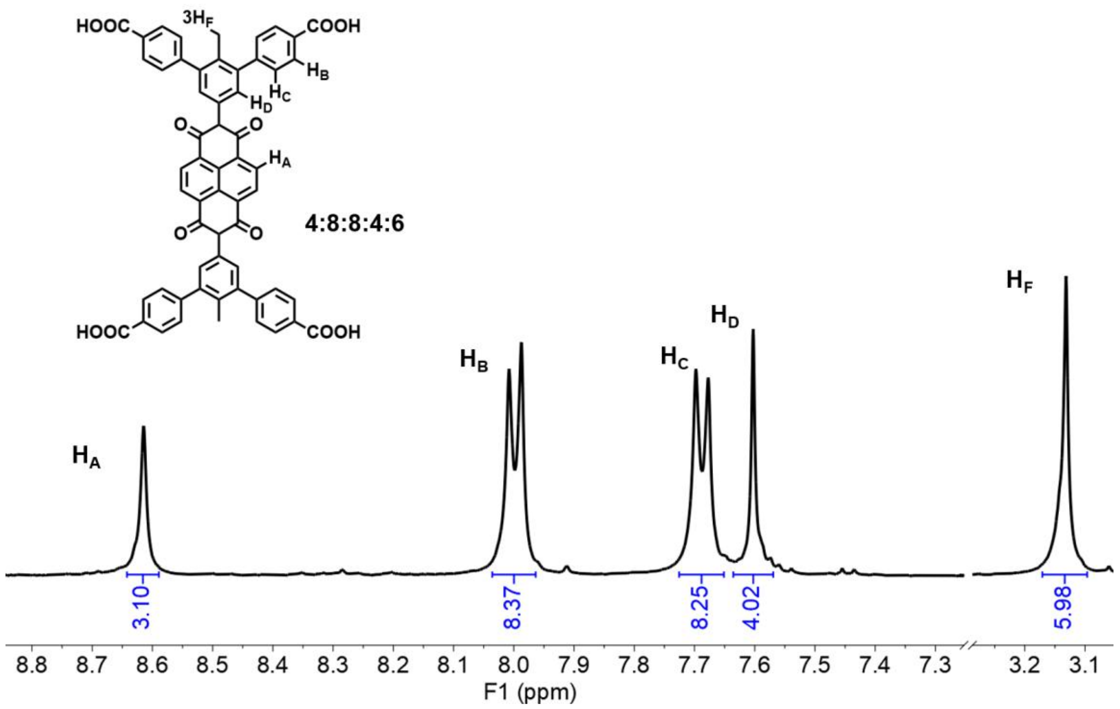

Figure S57. ^{1}H NMR (400 MHz, DMSO-d_6) spectrum of digested NKM-906-Me.

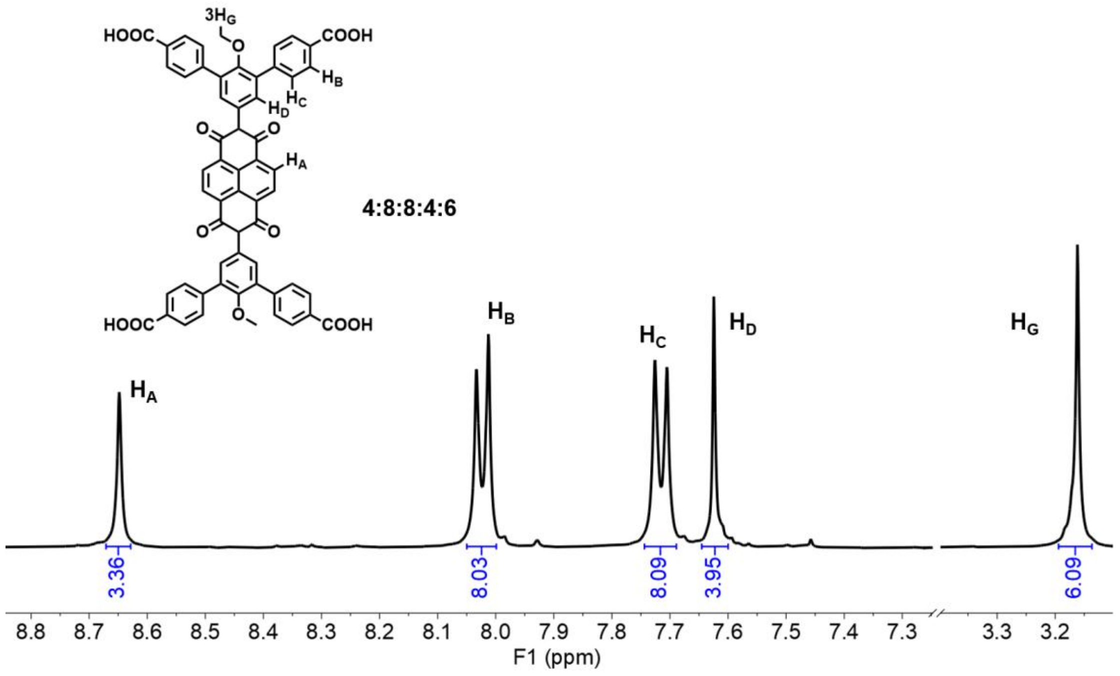

Figure S58. ^{1}H NMR (400 MHz, DMSO-d_6) spectrum of digested NKM-906-OMe.

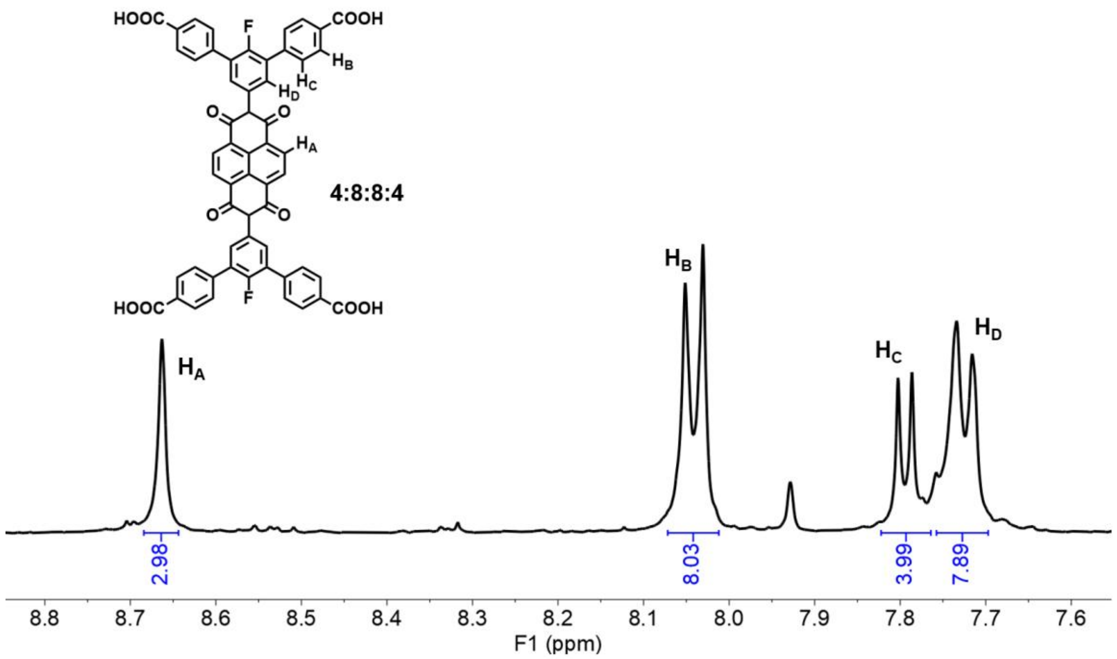

Figure S59. ^{1}H NMR (400 MHz, DMSO-d_6) spectrum of digested NKM-906-F.

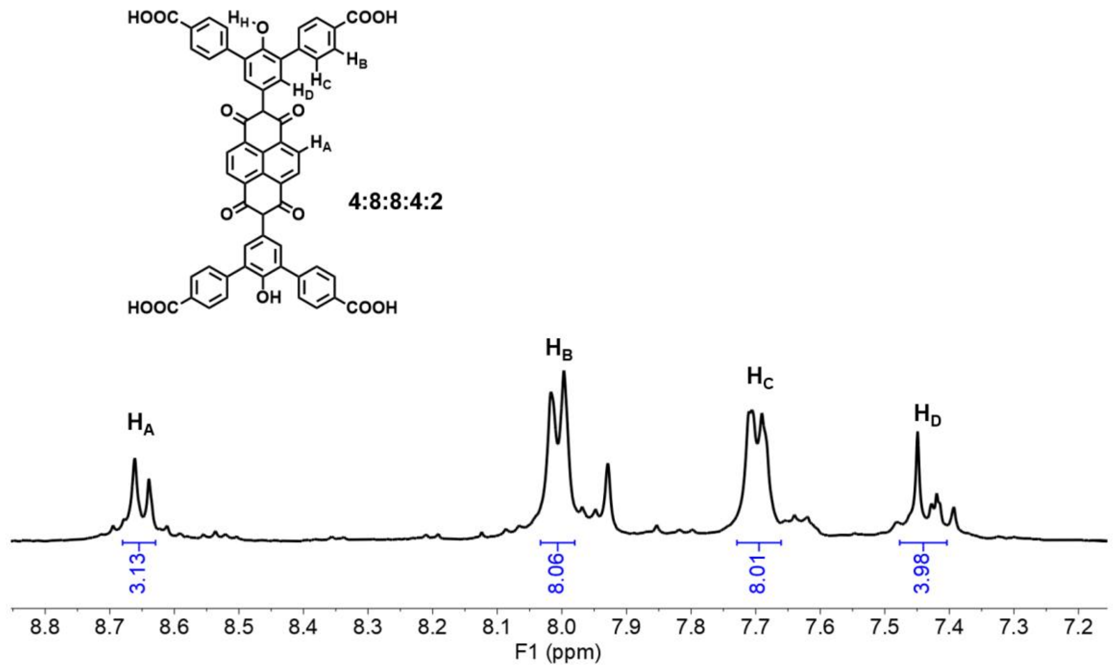

Figure S60. ^{1}H NMR (400 MHz, DMSO-d_6) spectrum of digested NKM-906-OH.

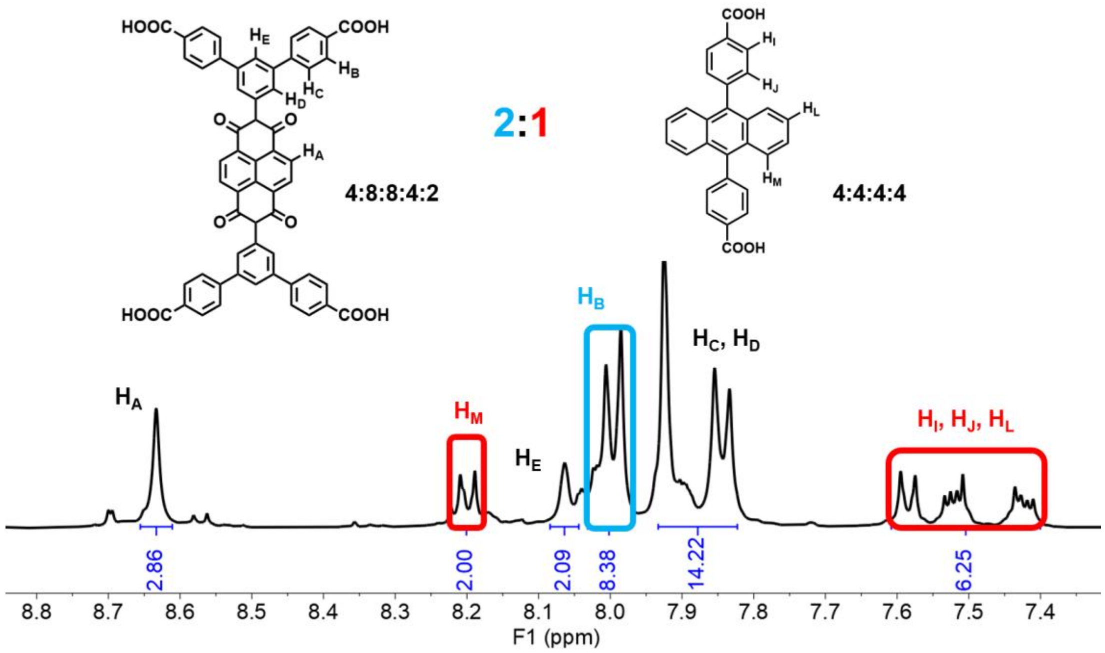

Figure S61. ^{1}H NMR (400 MHz, DMSO-d_6) spectrum of digested NKM-908-H-TPDC-AN.

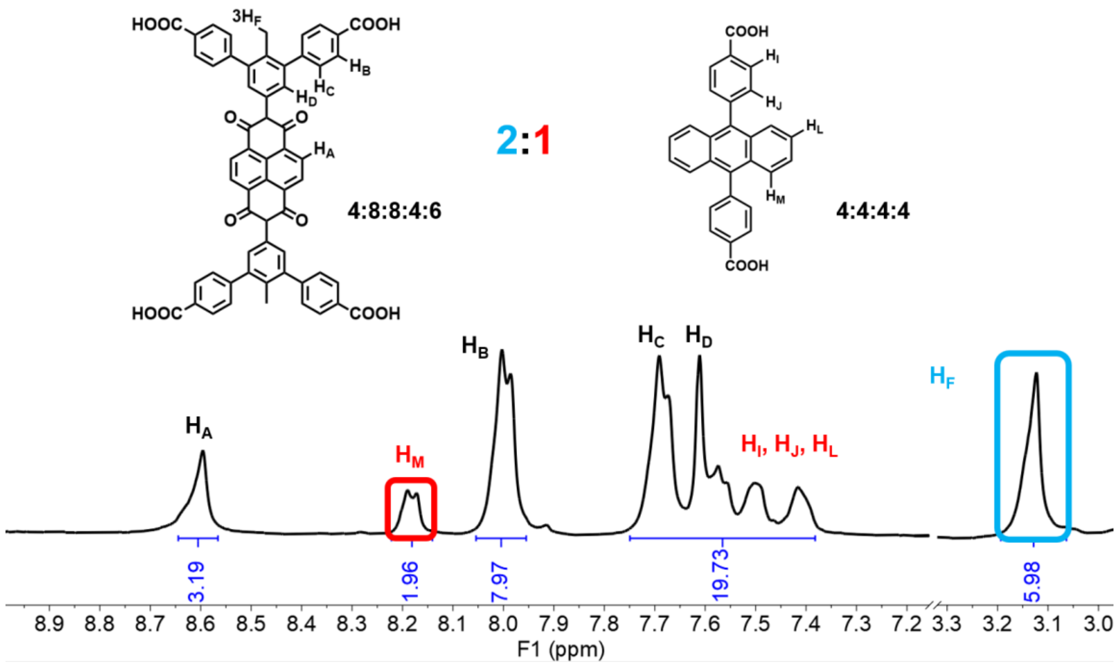

Figure S62. ^{1}H NMR (400 MHz, DMSO-d_6) spectrum of digested NKM-908-Me-TPDC-AN.

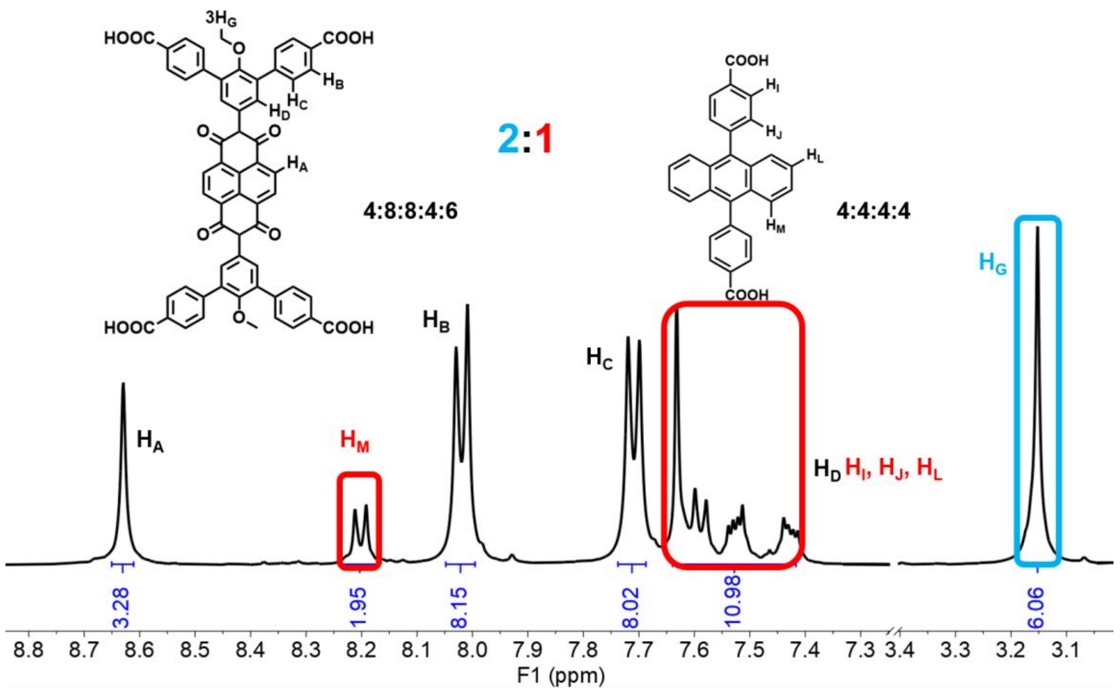

Figure S63. ^{1}H NMR (400 MHz, DMSO-d_6) spectrum of digested NKM-908-OMe-TPDC-AN.

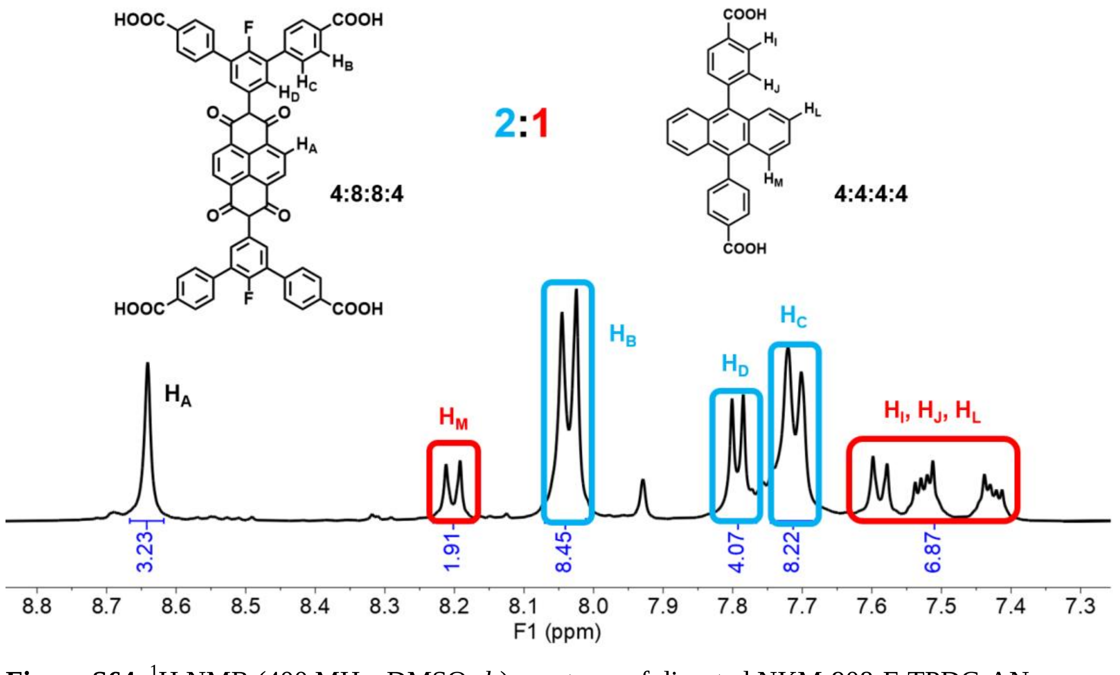

Figure S64. ^{1}H NMR (400 MHz, DMSO-d_6) spectrum of digested NKM-908-F-TPDC-AN.

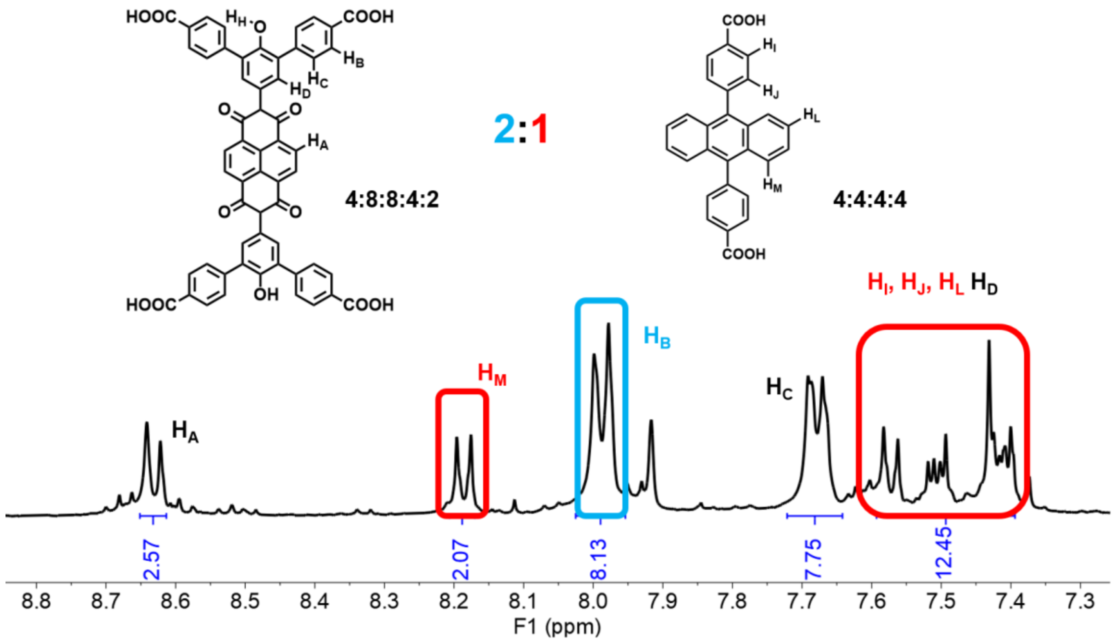

Figure S65. ^{1}H NMR (400 MHz, DMSO-d_6) spectrum of digested NKM-908-OH-TPDC-AN.

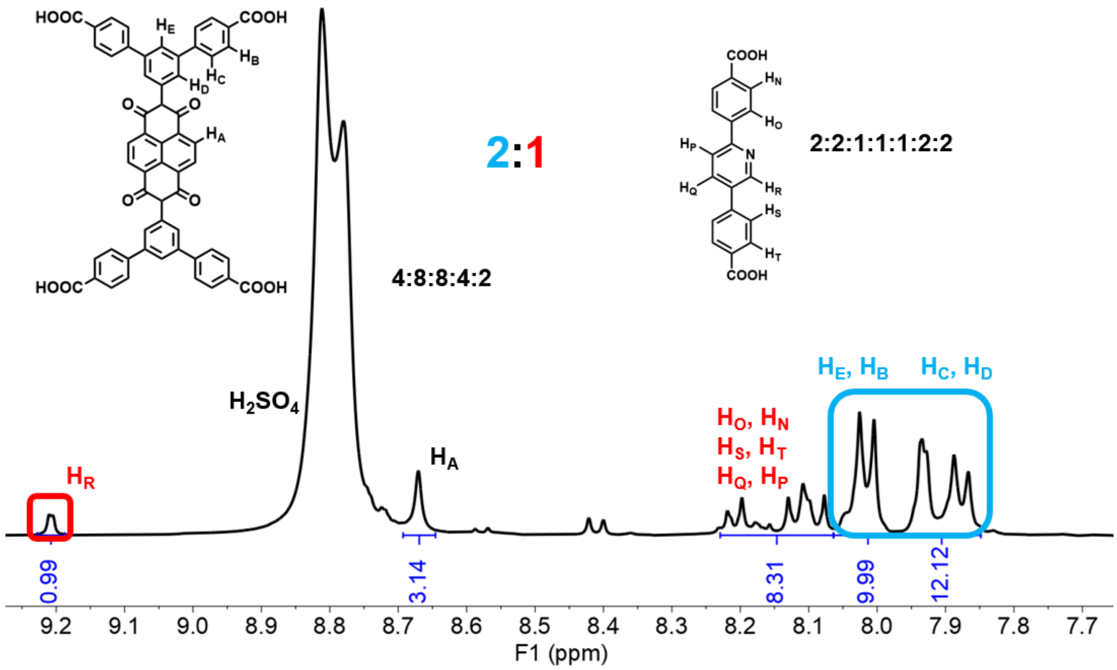

Figure S66. ^{1}H NMR (400 MHz, DMSO-d_6) spectrum of digested NKM-908-H-TPDC-Py.

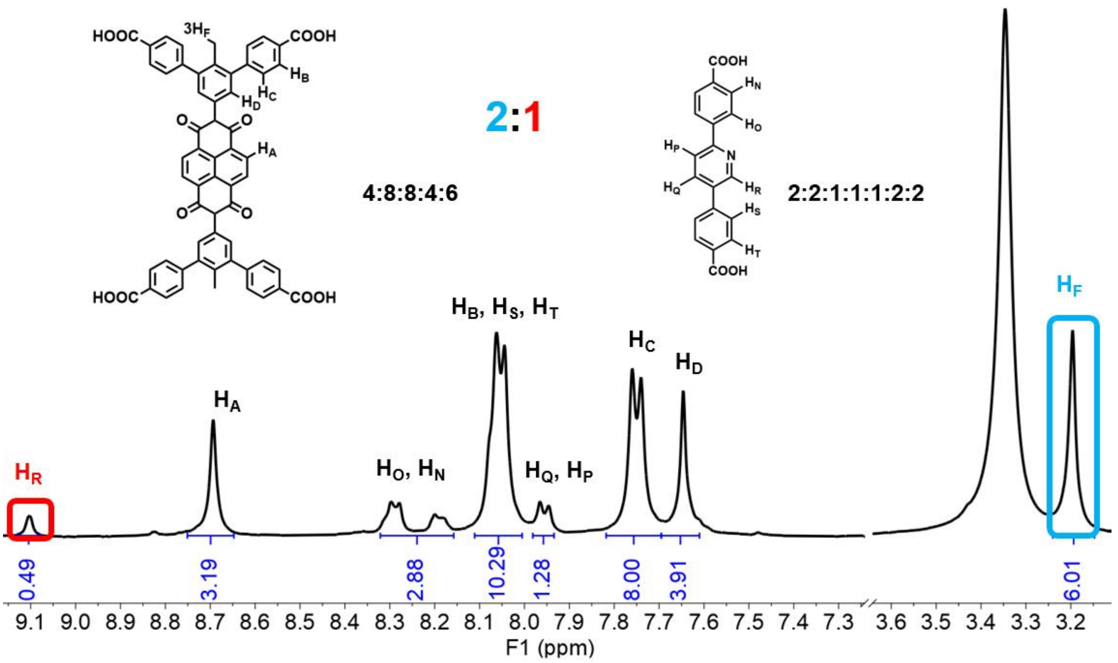

Figure S67. ^{1}H NMR (400 MHz, DMSO-d_6) spectrum of digested NKM-908-Me-TPDC-Py.

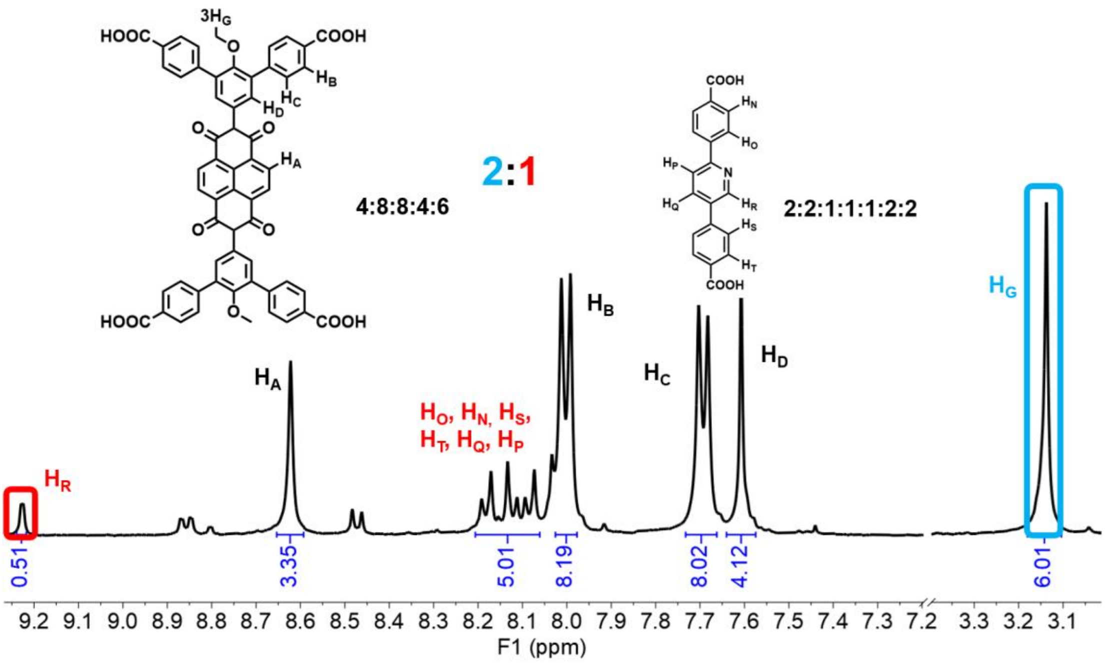

Figure S68. ^{1}H NMR (400 MHz, DMSO-d_6) spectrum of digested NKM-908-OMe-TPDC-Py.

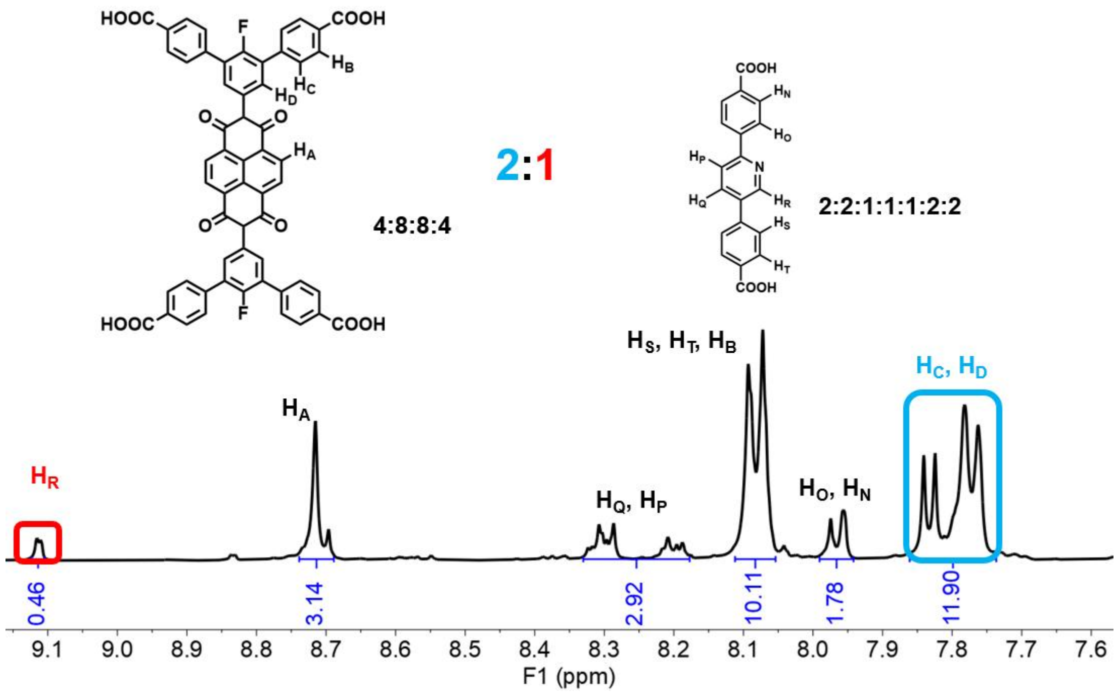

Figure S69. ^{1}H NMR (400 MHz, DMSO-d_6) spectrum of digested NKM-908-F-TPDC-Py.

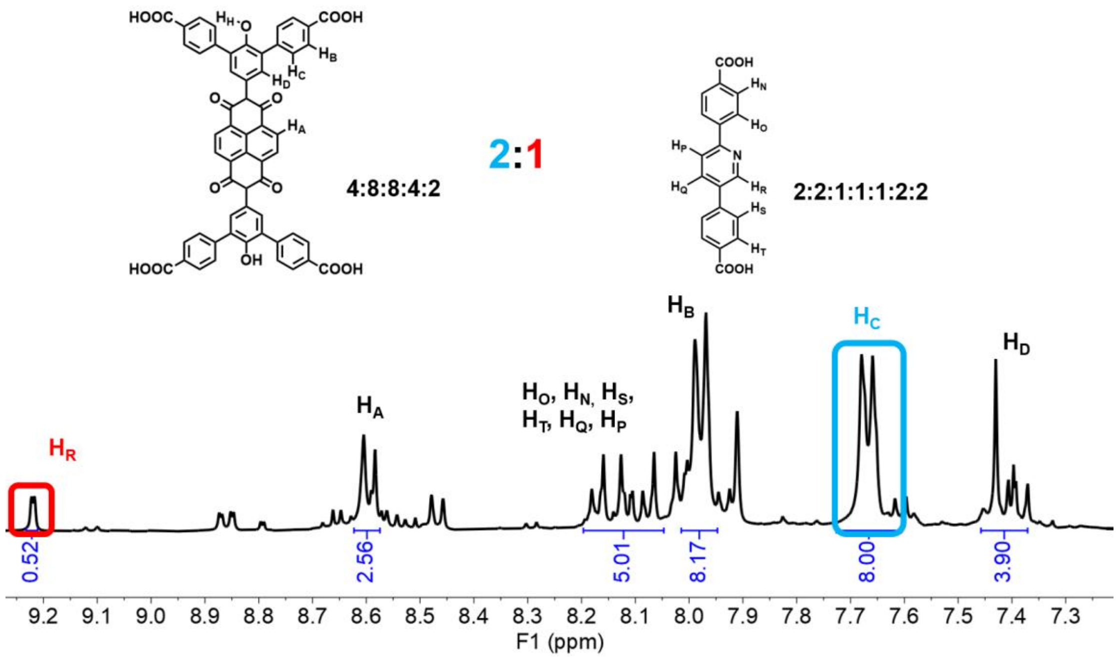

Figure S70. ^{1}H NMR (400 MHz, DMSO-d_6) spectrum of digested NKM-908-OH-TPDC-Py.

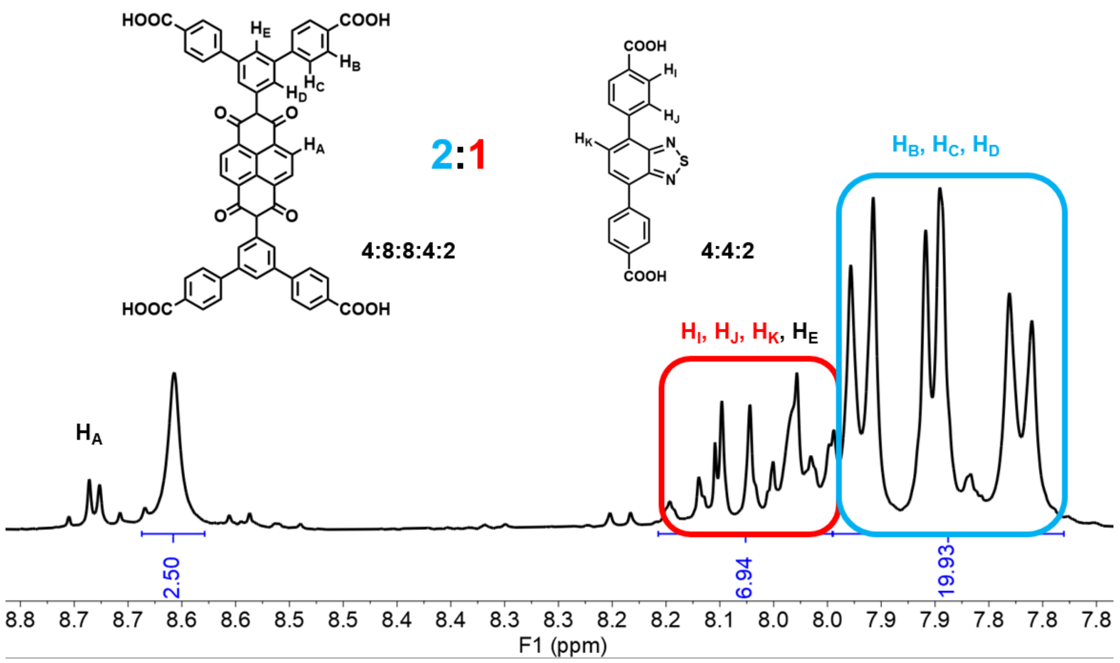

Figure S71. ^{1}H NMR (400 MHz, DMSO-d_6) spectrum of digested NKM-908-H-TPDC-TDA.

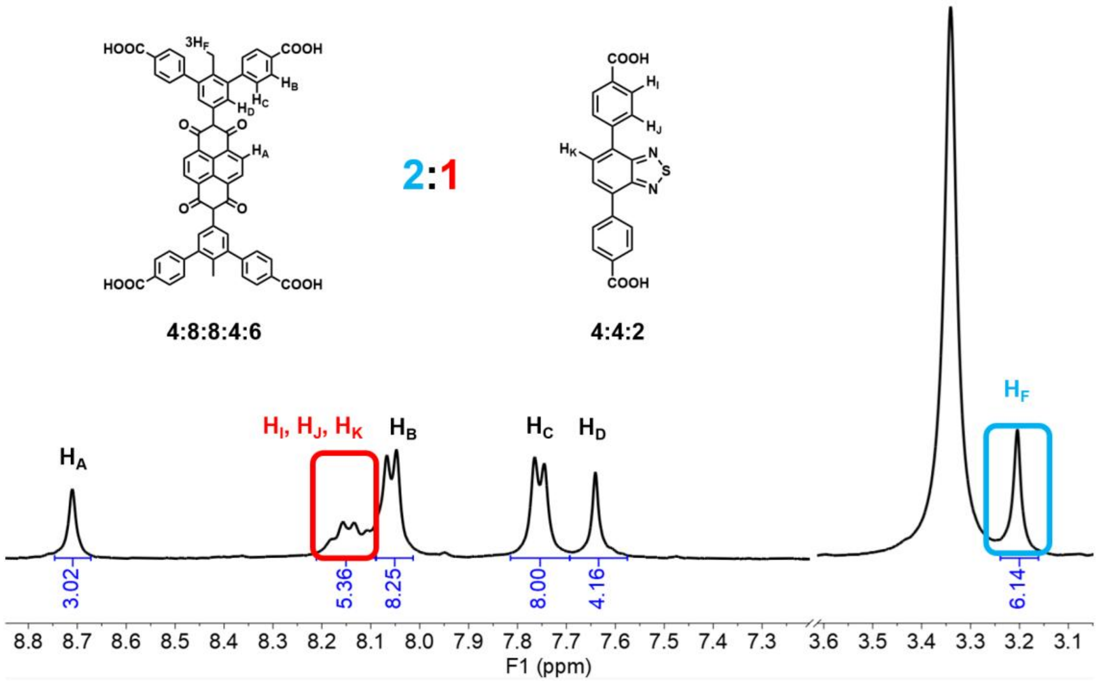

Figure S72. ^{1}H NMR (400 MHz, DMSO-d_6) spectrum of digested NKM-908-Me-TPDC-TDA.

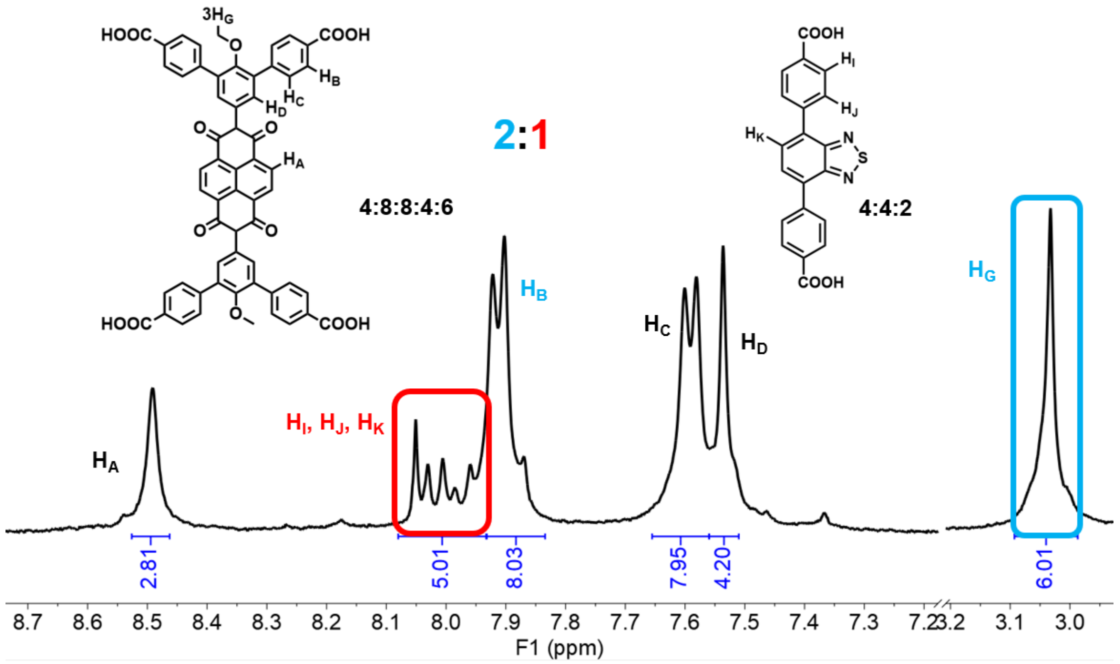

Figure S73. ^{1}H NMR (400 MHz, DMSO-d_6) spectrum of digested NKM-908-OMe-TPDC-TDA.

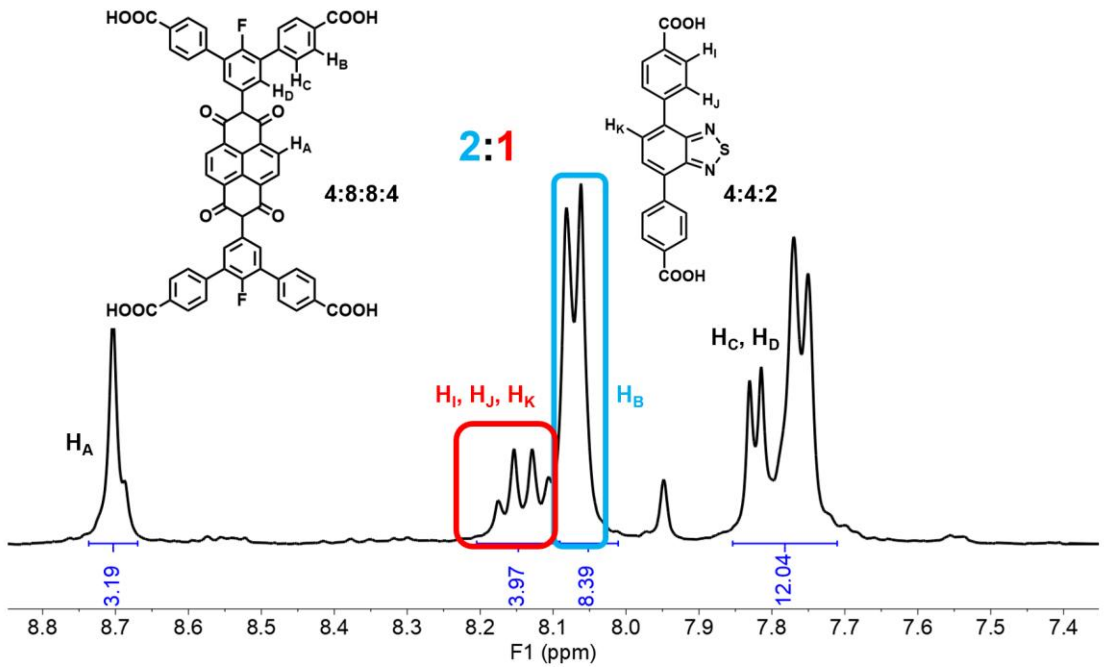

Figure S74. ^{1}H NMR (400 MHz, DMSO-d_6) spectrum of digested NKM-908-F-TPDC-TDA.

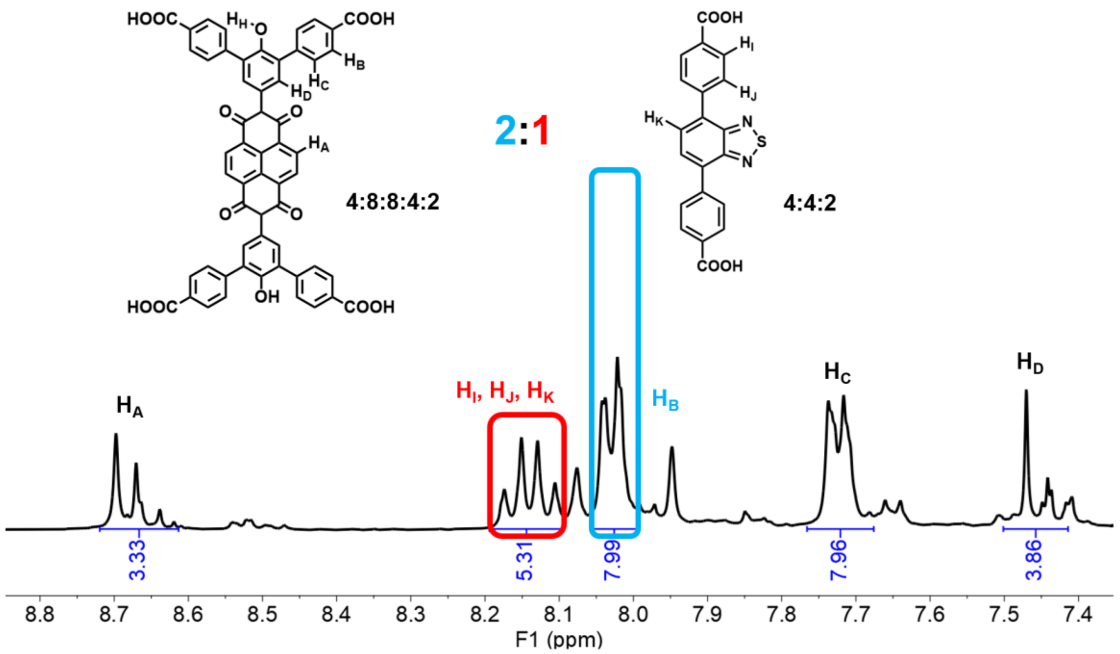

Figure S75. ^{1}H NMR (400 MHz, DMSO-d_6) spectrum of digested NKM-908-OH-TPDC-TDA.

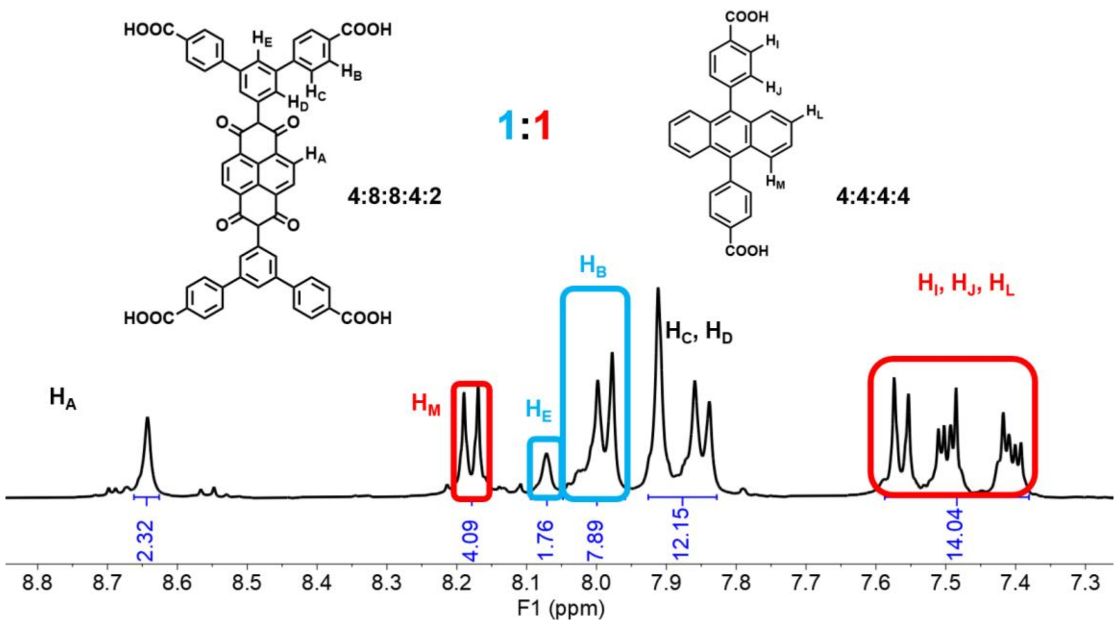

Figure S76. ^{1}H NMR (400 MHz, DMSO-d_6) spectrum of digested NKM-906-H-TPDC-AN.

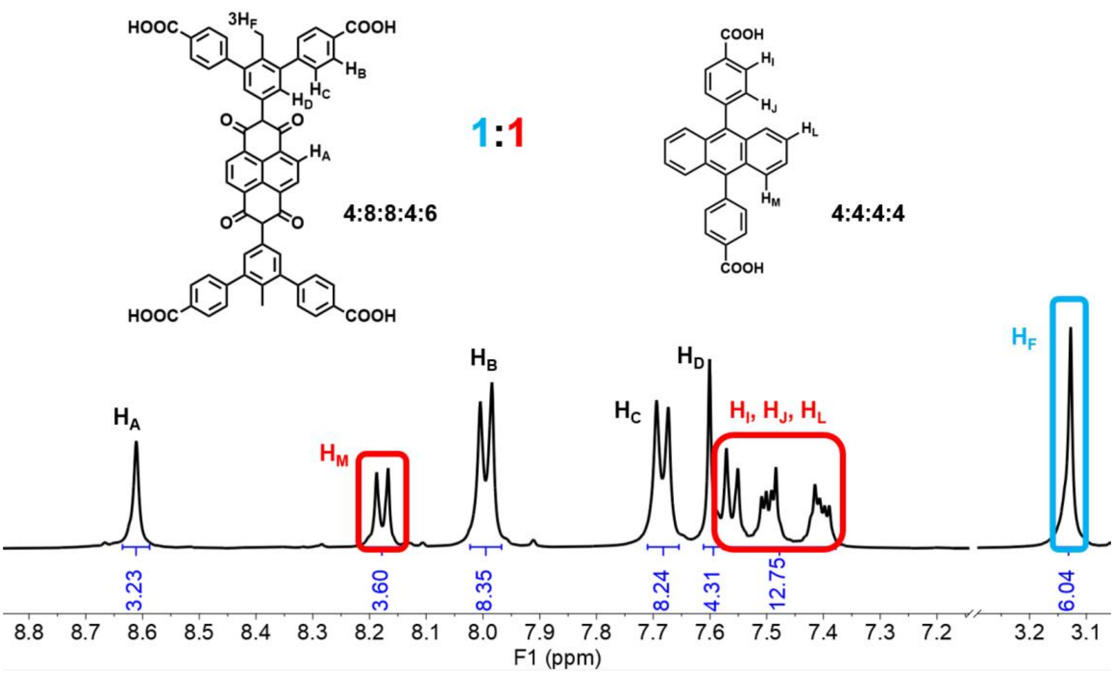

Figure S77. ^{1}H NMR (400 MHz, DMSO-d_6) spectrum of digested NKM-906-Me-TPDC-AN.

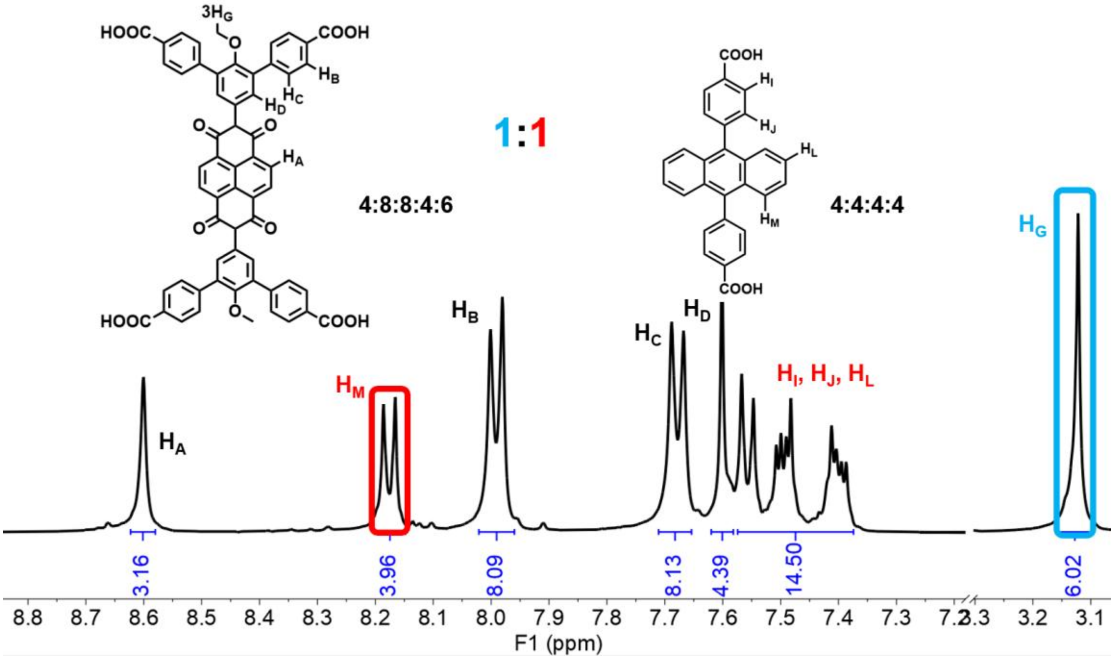

Figure S78. ^{1}H NMR (400 MHz, DMSO-d_6) spectrum of digested NKM-906-OMe-TPDC-AN.

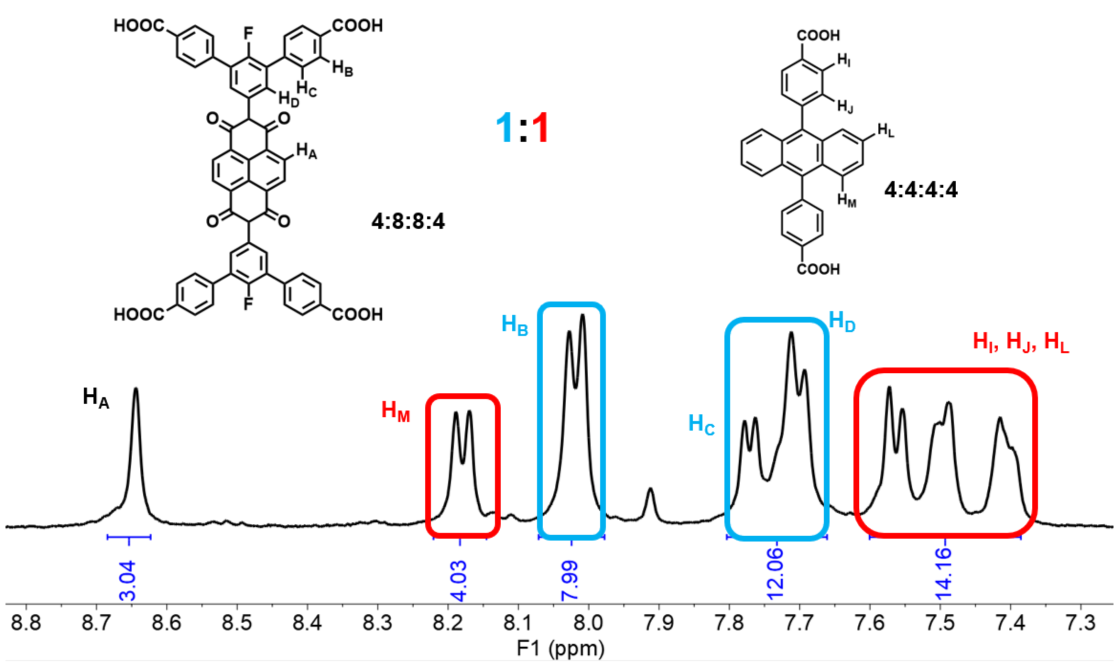

Figure S79. ^{1}H NMR (400 MHz, DMSO-d_6) spectrum of digested NKM-906-F-TPDC-AN.

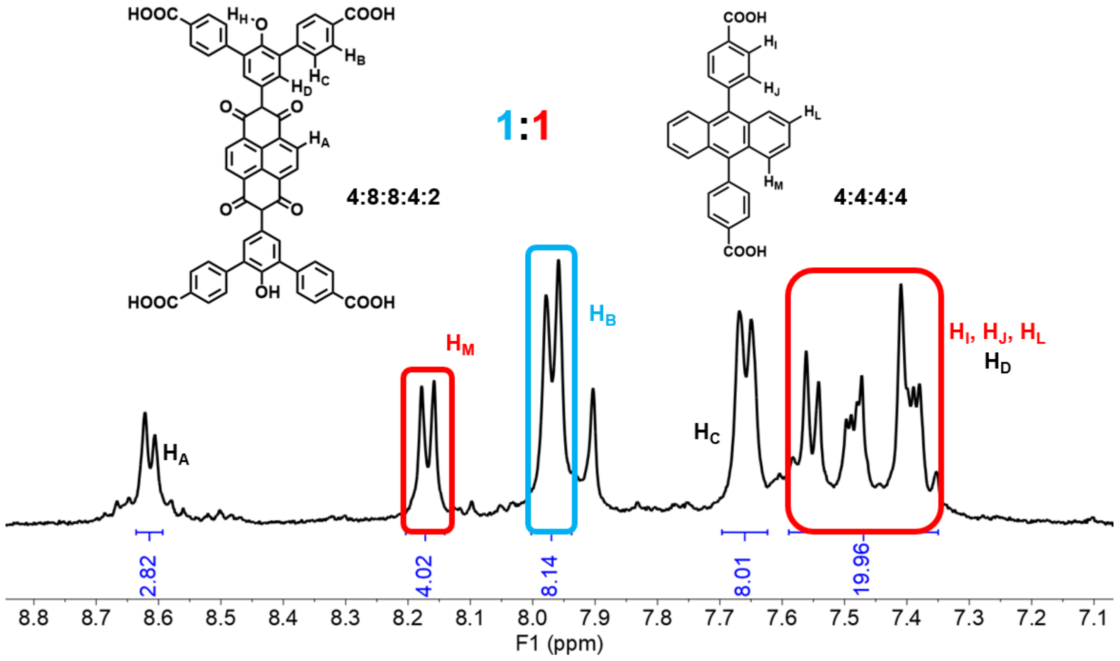

Figure S80. ^{1}H NMR (400 MHz, DMSO-d_6) spectrum of digested NKM-906-OH-TPDC-AN.

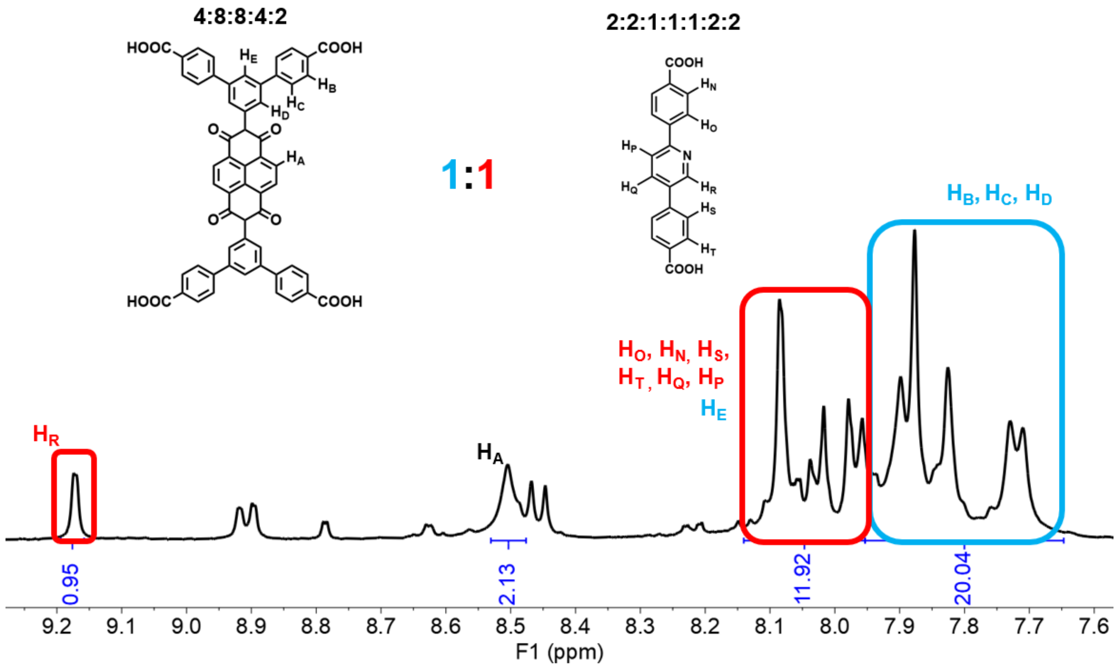

Figure S81. ^{1}H NMR (400 MHz, DMSO-d_6) spectrum of digested NKM-906-H-TPDC-Py.

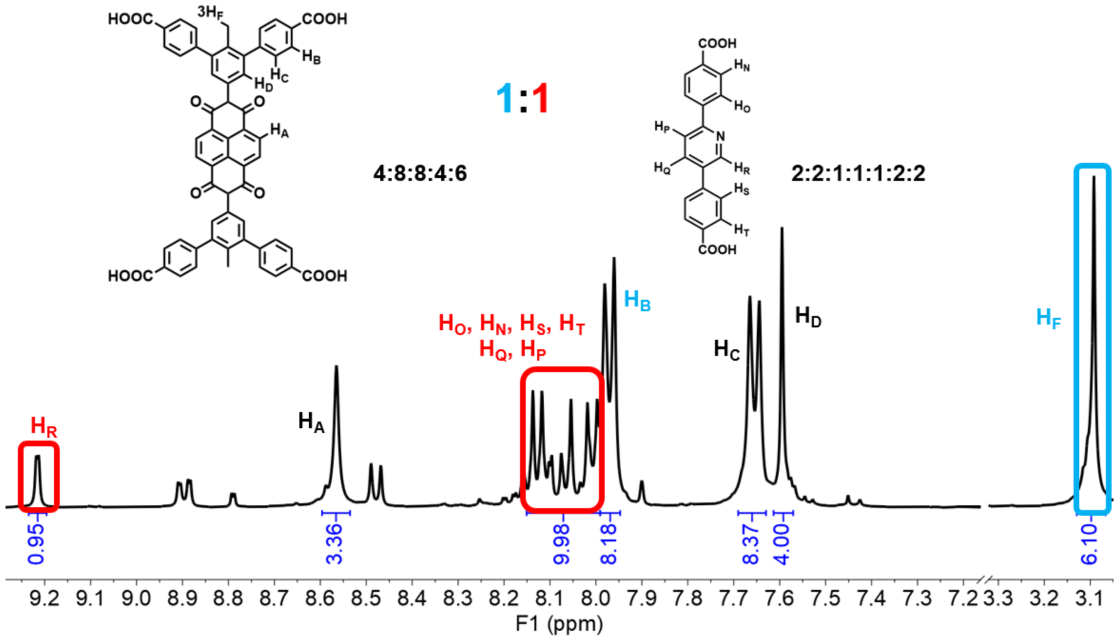

Figure S82. ^{1}H NMR (400 MHz, DMSO-d_6) spectrum of digested NKM-906-Me-TPDC-Py.

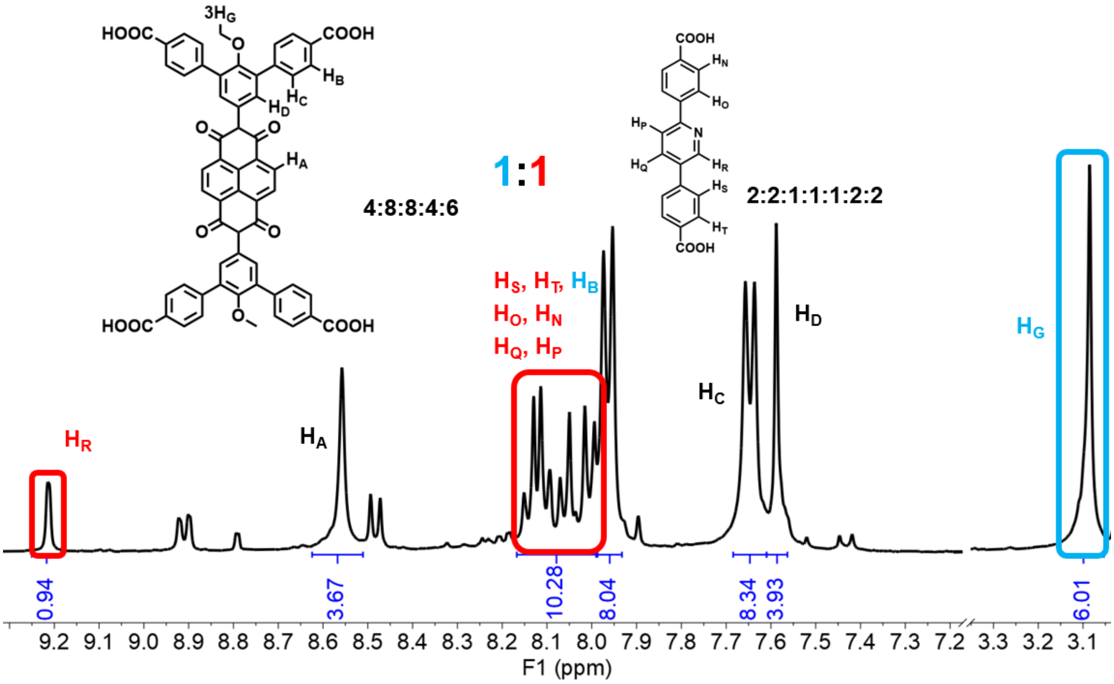

Figure S83. ^{1}H NMR (400 MHz, DMSO-d_6) spectrum of digested NKM-906-OMe-TPDC-Py.

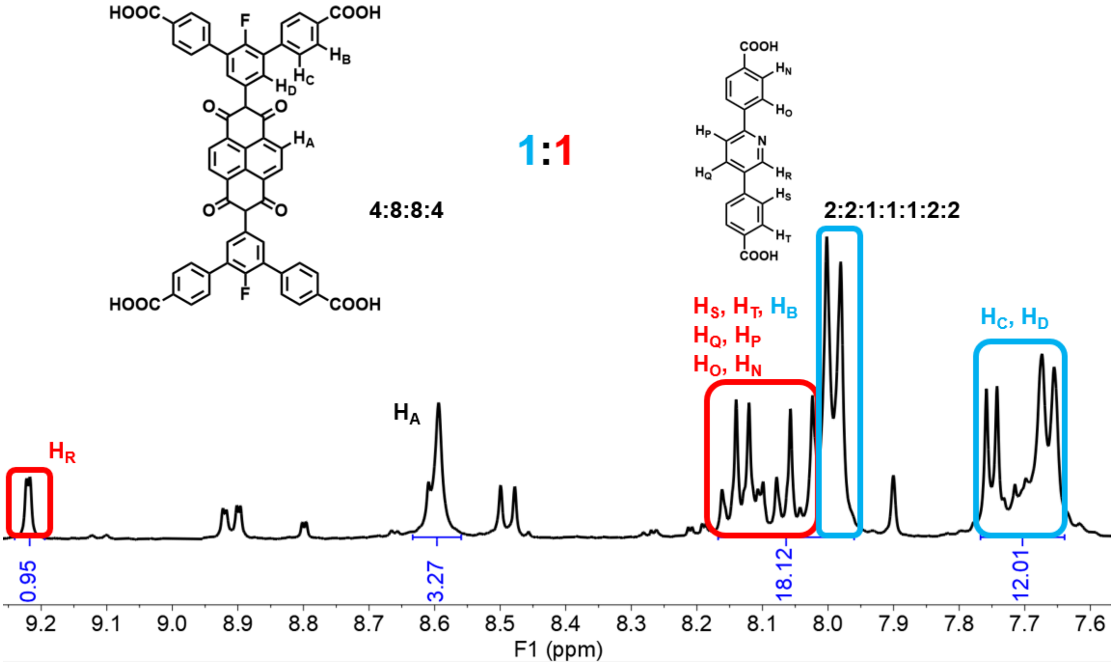

Figure S84. ^{1}H NMR (400 MHz, DMSO-d_6) spectrum of digested NKM-906-F-TPDC-Py.

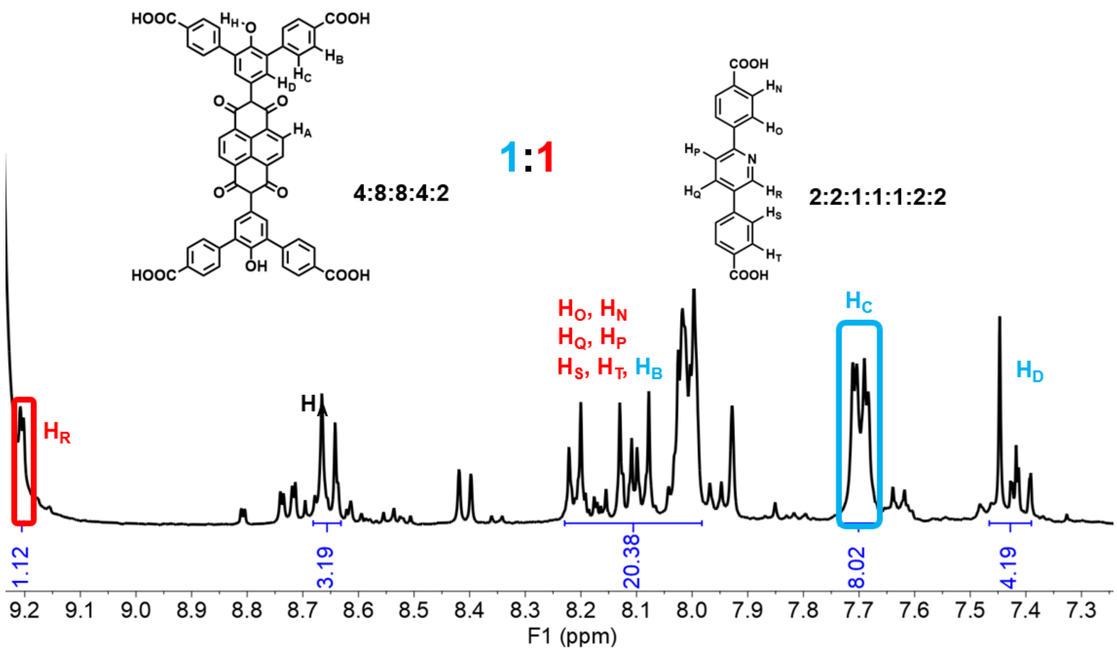

Figure S85. ^{1}H NMR (400 MHz, DMSO-d_6) spectrum of digested NKM-906-OH-TPDC-Py.

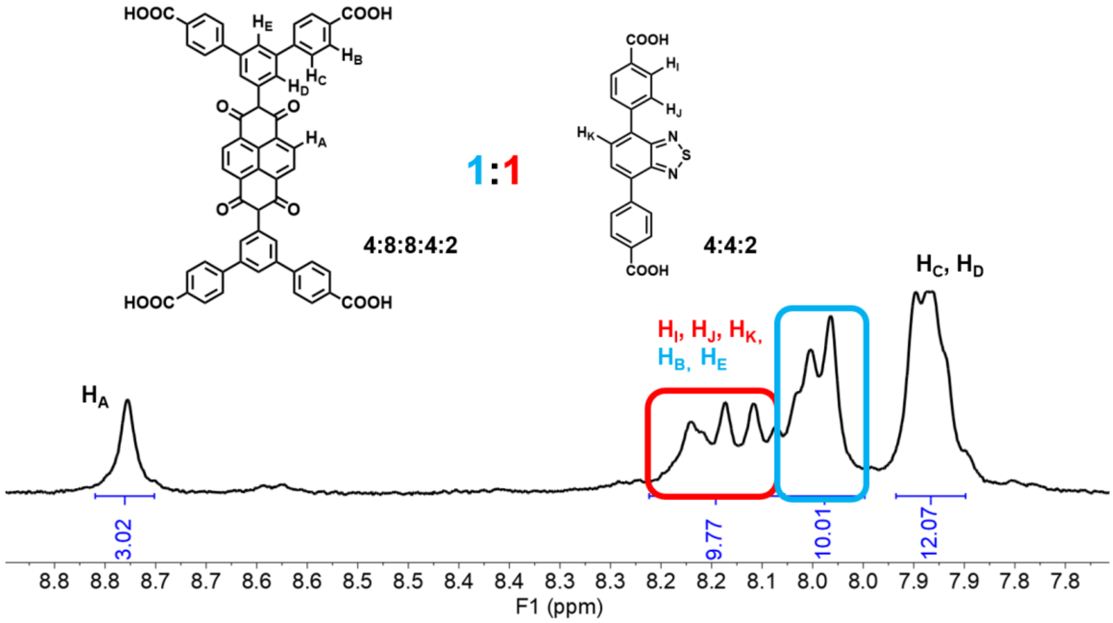

Figure S86. ^{1}H NMR (400 MHz, DMSO-d_6) spectrum of digested NKM-906-H-TPDC-TDA.

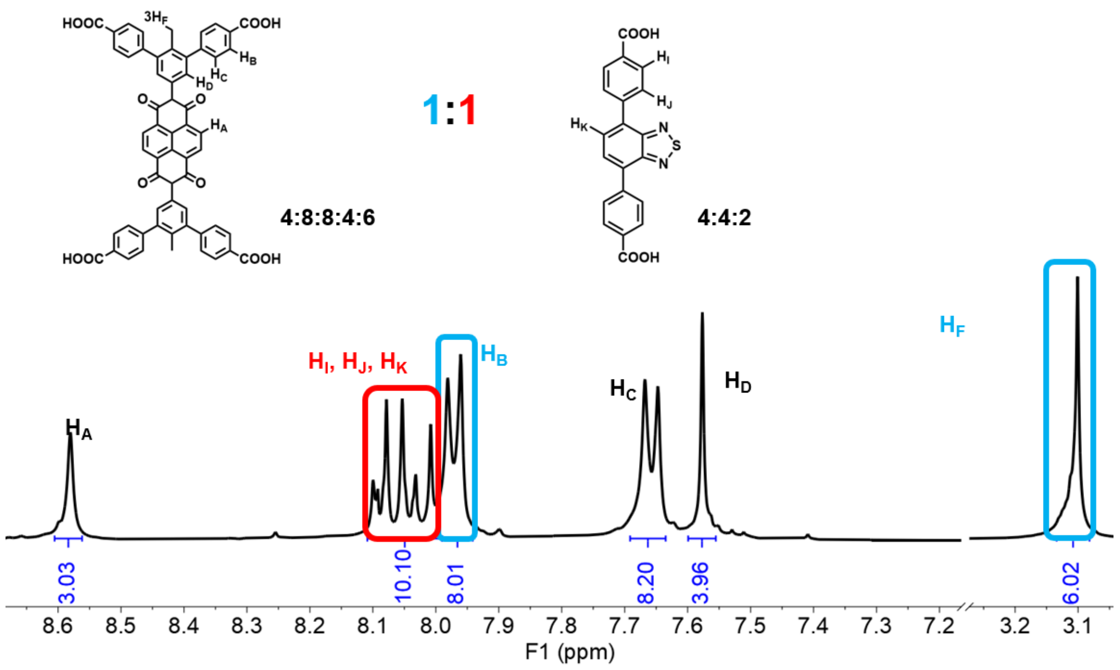

Figure S87. ^{1}H NMR (400 MHz, DMSO-d_6) spectrum of digested NKM-906-Me-TPDC-TDA.

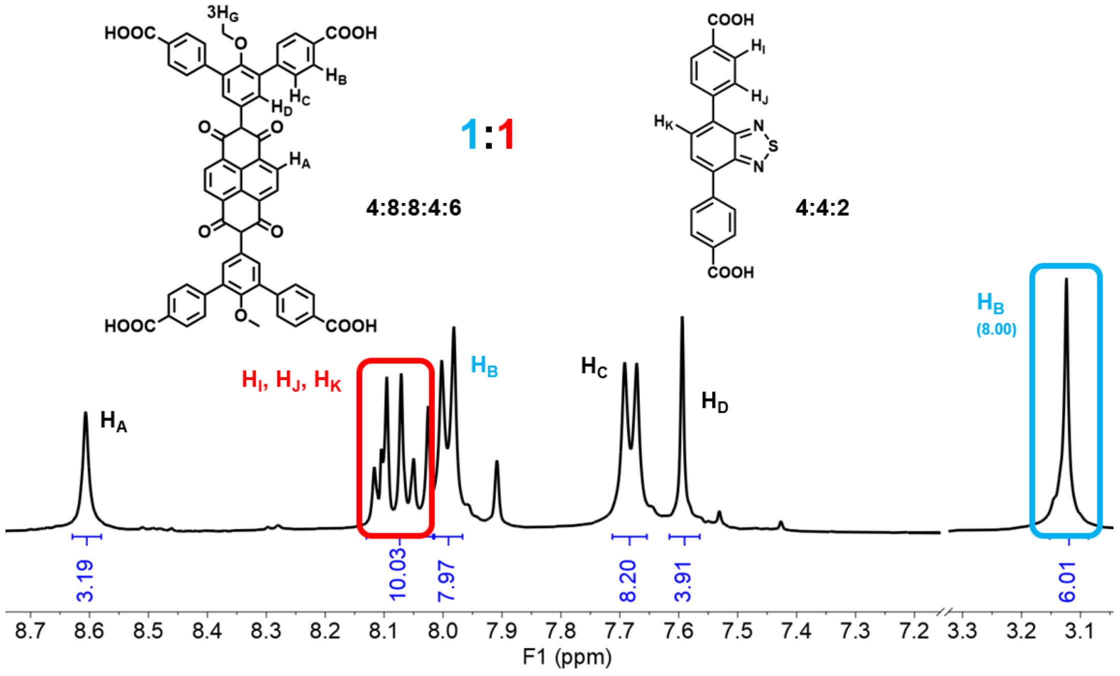

Figure S88. ^{1}H NMR (400 MHz, DMSO-d_6) spectrum of digested NKM-906-OMe-TPDC-TDA.

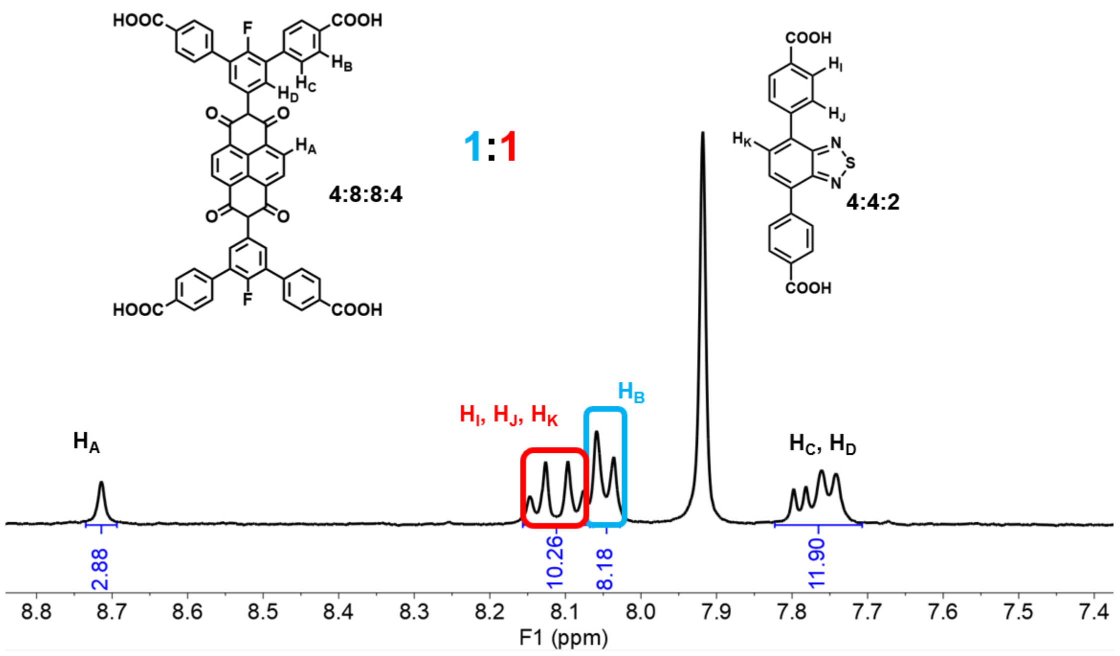

Figure S89. ^{1}H NMR (400 MHz, DMSO-d_6) spectrum of digested NKM-906-F-TPDC-TDA.

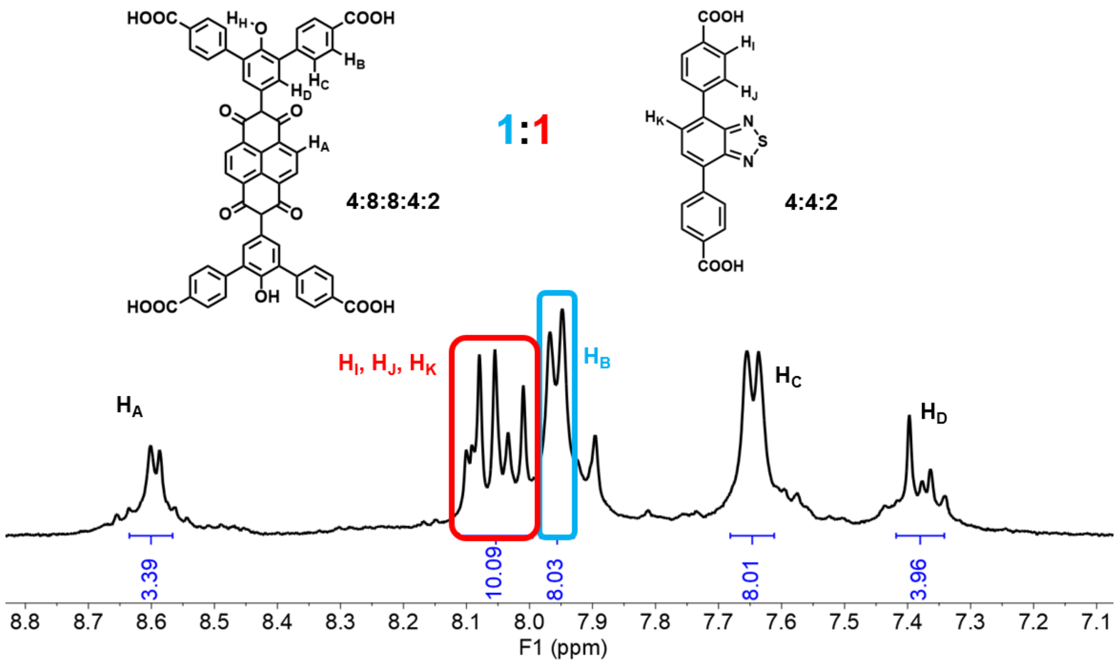

Figure S90. ^{1}H NMR (400 MHz, DMSO-d_6) spectrum of digested NKM-906-OH-TPDC-TDA.

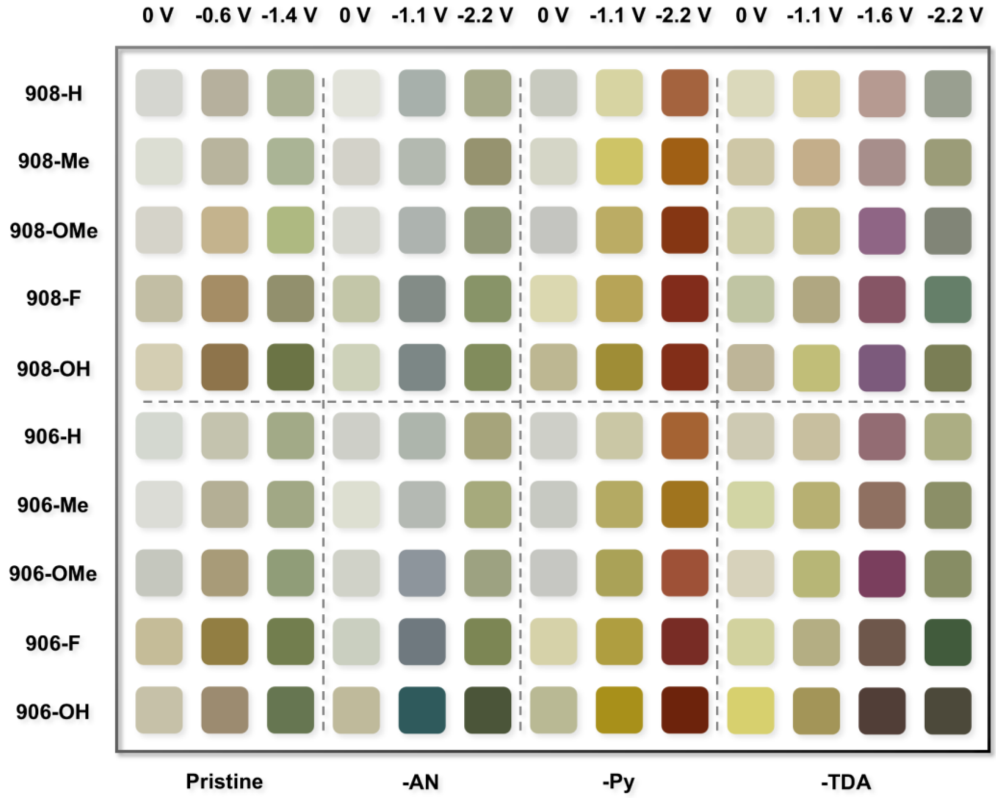

Figure S91. The acquired reference colors for all forty MOF thin films at representative potentials during electrochromic processes according to **Figure 4** (wet). These pure colors were converted into the RBG and HSL coordinates as of in **Table S5** for further analyses.

Table S5. Summary of reference labels and representative potentials of all forty MOF thin films during electrochromic process, and the corresponding RGB/HSL coordinates of the colors.

Compound	Potential (V)	Label	R	G	B	H (°)	S (%)	L (%)
**NKM-908-H**	0.0	1-I	200	201	195	70.00	5.26	77.65
	-0.6	1-II	170	165	146	47.50	12.37	61.96
	-1.4	1-III	160	166	138	72.86	13.59	59.61
**NKM-908-Me**	0.0	2-I	207	209	198	70.91	10.68	79.80
	-0.6	2-II	172	168	146	50.77	13.54	62.35
	-1.4	2-III	159	168	139	78.62	14.29	60.20
**NKM-908-OMe**	0.0	3-I	200	198	189	49.09	9.09	76.27
	-0.6	3-II	184	167	131	40.75	27.18	61.76
	-1.4	3-III	163	179	119	76.00	28.30	58.43
**NKM-908-F**	0.0	4-I	182	178	153	51.72	16.57	65.69
	-0.6	4-II	154	131	92	37.74	25.20	48.24
	-1.4	4-III	136	134	100	56.67	15.25	46.27
**NKM-908-OH**	0.0	5-I	199	193	167	48.75	22.22	71.76
	-0.6	5-II	132	107	67	36.92	32.66	39.02
	-1.4	5-III	98	107	62	72.00	26.63	33.14
**NKM-906-H**	0.0	6-I	199	203	195	90.00	7.14	78.04
	-0.6	6-II	184	183	163	57.14	12.88	68.04
	-1.4	6-III	151	159	125	74.12	15.04	55.69
**NKM-906-Me**	0.0	7-I	206	207	201	70.00	5.88	80.00
	-0.6	7-II	168	164	139	51.72	14.29	60.20
	-1.4	7-III	150	157	123	72.35	14.78	54.90
**NKM-906-OMe**	0.0	8-I	185	187	178	73.33	6.21	71.57
	-0.6	8-II	157	144	111	43.04	19.00	52.54
	-1.4	8-III	134	146	111	80.57	13.83	50.39
**NKM-906-F**	0.0	9-I	185	176	141	47.73	23.91	63.92
	-0.6	9-II	136	116	59	44.42	39.49	38.24
	-1.4	9-III	105	116	70	74.35	24.73	36.47
**NKM-906-OH**	0.0	10-I	186	181	157	49.66	17.37	67.25
	-0.6	10-II	154	136	99	40.36	21.74	49.61
	-1.4	10-III	93	109	73	86.67	19.78	35.69
**NKM-908-H-TPDC-AN**	0.0	11-I	213	214	205	66.67	9.90	82.16
	-1.1	11-II	156	165	160	146.67	4.76	62.94
	-2.2	11-III	156	159	128	65.81	13.90	56.27
**NKM-908-Me-TPDC-AN**	0.0	12-I	198	197	189	53.33	7.32	75.88
	-1.1	12-II	167	173	165	105.00	4.65	66.27
	-2.2	12-III	140	137	102	55.26	15.70	47.45
**NKM-908-OMe-TPDC-AN**	0.0	13-I	202	203	195	67.50	7.14	78.04
	-1.1	13-II	162	167	164	144.00	2.76	64.51
	-2.2	13-III	136	141	111	70.00	11.90	49.41
**NKM-908-F-TPDC-AN**	0.0	14-I	183	186	157	66.21	17.37	67.25
	-1.1	14-II	121	130	125	146.67	3.59	49.22
	-2.2	14-III	126	138	95	76.74	18.45	45.69
**NKM-908-OH-TPDC-AN**	0.0	15-I	193	197	174	70.43	16.55	72.75
	-1.1	15-II	115	125	124	174.00	4.17	47.06
	-2.2	15-III	119	130	84	74.35	21.50	41.96
**NKM-906-H-TPDC-AN**	0.0	16-I	193	194	188	70.00	4.69	74.90
	-1.1	16-II	162	169	161	112.50	4.44	64.71
	-2.2	16-III	155	153	114	57.07	17.01	52.75

Table S5. (Continued).

Compound	Potential (V)	Label	R	G	B	H (°)	S (%)	L (%)
**NKM-906-Me-TPDC-AN**	0.0	17-I	208	210	196	68.57	13.46	79.61
	-1.1	17-II	168	173	167	110.00	3.53	66.67
	-2.2	17-III	155	159	115	65.45	18.64	53.73
**NKM-906-OMe-TPDC-AN**	0.0	18-I	195	197	188	73.33	7.20	75.49
	-1.1	18-II	131	139	145	205.71	5.98	54.12
	-2.2	18-III	146	151	119	69.38	13.33	52.94
**NKM-906-F-TPDC-AN**	0.0	19-I	191	194	180	77.14	10.29	73.33
	-1.1	19-II	102	112	117	200.00	6.85	42.94
	-2.2	19-III	115	124	76	71.25	24.00	39.22
**NKM-906-OH-TPDC-AN**	0.0	20-I	179	174	144	51.43	18.72	63.33
	-1.1	20-II	40	82	84	182.73	35.48	24.31
	-2.2	20-III	67	77	50	82.22	21.26	24.90
**NKM-908-H-TPDC-Py**	0.0	21-I	188	190	179	70.91	7.80	72.35
	-1.1	21-II	202	199	151	56.47	32.48	69.22
	-2.2	21-III	153	90	55	21.65	46.86	40.59
**NKM-908-Me-TPDC-Py**	0.0	22-I	200	201	187	64.29	11.48	76.08
	-1.1	22-II	193	184	93	54.60	44.64	56.08
	-2.2	22-III	149	87	14	32.44	82.82	31.96
**NKM-908-OMe-TPDC-Py**	0.0	23-I	184	185	180	72.00	3.45	71.57
	-1.1	23-II	175	161	91	50.00	34.43	52.16
	-2.2	23-III	123	47	13	18.55	80.88	26.67
**NKM-908-F-TPDC-Py**	0.0	24-I	206	203	165	55.61	29.50	72.74
	-1.1	24-II	171	153	79	48.26	36.80	49.02
	-2.2	24-III	120	38	21	10.30	70.21	27.65
**NKM-908-OH-TPDC-Py**	0.0	25-I	177	171	136	51.22	20.81	61.37
	-1.1	25-II	148	131	47	49.90	51.79	38.24
	-2.2	25-III	120	39	18	12.35	73.91	27.06
**NKM-906-H-TPDC-Py**	0.0	26-I	193	194	188	70.00	4.69	74.90
	-1.1	26-II	190	187	154	55.00	21.69	67.45
	-2.2	26-III	154	90	44	25.09	55.56	38.82
**NKM-906-Me-TPDC-Py**	0.0	27-I	187	189	182	77.14	5.04	72.75
	-1.1	27-II	168	159	90	53.08	30.95	50.59
	-2.2	27-III	149	107	24	39.84	72.25	33.92
**NKM-906-OMe-TPDC-Py**	0.0	28-I	186	187	182	72.00	3.55	72.35
	-1.1	28-II	159	151	79	54.00	33.61	46.67
	-2.2	28-III	147	73	48	15.15	50.77	38.24
**NKM-906-F-TPDC-Py**	0.0	29-I	201	197	158	54.42	28.48	70.39
	-1.1	29-II	164	147	57	50.47	48.42	43.33
	-2.2	29-III	111	38	31	5.25	56.34	27.84
**NKM-906-OH-TPDC-Py**	0.0	30-I	173	173	138	60.00	17.59	60.98
	-1.1	30-II	157	134	20	49.93	77.40	34.71
	-2.2	30-III	100	29	7	14.19	86.92	20.98
**NKM-908-H-TPDC-TDA**	0.0	31-I	206	204	175	56.13	24.03	74.71
	-1.1	31-II	201	193	149	50.77	32.50	68.63
	-1.6	31-III	170	143	135	13.71	17.07	59.80
	-2.2	31-IV	142	148	134	85.71	6.14	55.29
**NKM-908-Me-TPDC-TDA**	0.0	32-I	193	187	155	50.53	23.46	68.24
	-1.1	32-II	184	163	128	37.50	28.28	61.18
	-1.6	32-III	156	132	129	6.67	12.00	55.88
	-2.2	32-IV	144	145	111	61.76	13.38	50.20

Table S5. (Continued).

Compound	Potential (V)	Label	R	G	B	H (°)	S (%)	L (%)
**NKM-908-OMe-TPDC-TDA**	0.0	33-I	193	192	156	58.38	22.98	68.43
	-1.1	33-II	179	172	126	52.08	25.85	59.80
	-1.6	33-III	133	92	123	314.63	18.22	44.11
	-2.2	33-IV	119	123	110	78.46	5.58	45.69
**NKM-908-F-TPDC-TDA**	0.0	34-I	180	185	152	69.09	19.08	66.08
	-1.1	34-II	165	156	119	48.26	20.35	55.69
	-1.6	34-III	124	77	92	340.85	23.38	39.41
	-2.2	34-IV	92	117	96	129.60	11.96	40.98
**NKM-908-OH-TPDC-TDA**	0.0	35-I	178	169	141	45.40	19.37	62.55
	-1.1	35-II	181	178	111	57.43	32.11	57.26
	-1.6	35-III	115	82	115	300.00	16.75	38.63
	-2.2	35-IV	113	116	77	64.61	20.21	37.84
**NKM-906-H-TPDC-TDA**	0.0	36-I	193	190	167	53.08	17.33	70.59
	-1.1	36-II	188	179	148	46.50	22.99	65.88
	-1.6	36-III	137	99	106	348.95	16.10	46.28
	-2.2	36-IV	161	163	121	62.86	18.58	55.69
**NKM-906-Me-TPDC-TDA**	0.0	37-I	197	200	153	63.83	29.94	69.22
	-1.1	37-II	171	165	105	54.55	28.20	54.12
	-1.6	37-III	133	103	89	19.09	19.82	43.53
	-2.2	37-IV	129	133	94	66.15	17.18	44.51
**NKM-906-OMe-TPDC-TDA**	0.0	38-I	202	197	175	48.89	20.30	73.92
	-1.1	38-II	171	170	109	59.03	26.96	54.90
	-1.6	38-III	113	55	85	328.97	34.52	32.94
	-2.2	38-IV	125	131	91	69.00	18.02	43.53
**NKM-906-F-TPDC-TDA**	0.0	39-I	197	197	147	60.00	30.12	67.45
	-1.1	39-II	168	163	121	53.62	21.27	56.67
	-1.6	39-III	101	79	67	21.18	20.24	32.94
	-2.2	39-IV	58	83	53	110.00	22.06	26.67
**NKM-906-OH-TPDC-TDA**	0.0	40-I	202	195	101	55.84	48.79	59.41
	-1.1	40-II	152	139	80	49.17	31.03	45.49
	-1.6	40-III	73	55	48	16.80	20.66	23.72
	-2.2	40-IV	68	65	51	49.41	14.29	23.33

Note: sRGB (0-255) model was used, and were converted into HSL coordinates accordingly. These representative potentials were not those where the redox events started to occur, but were the “turning points” of absorption behaviors.

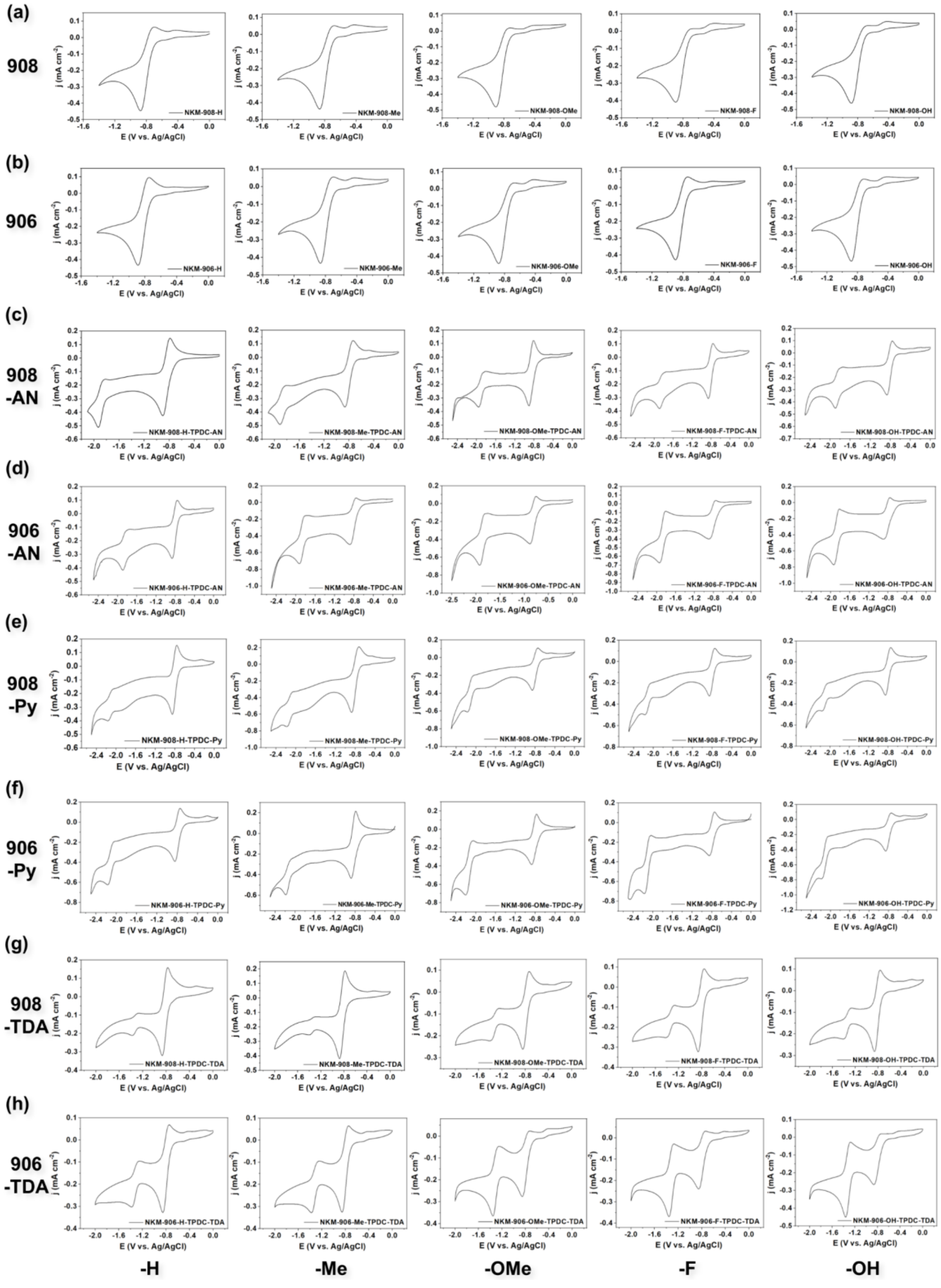

Figure S92. CV curves of all forty MOF thin films over one electrochromic process.

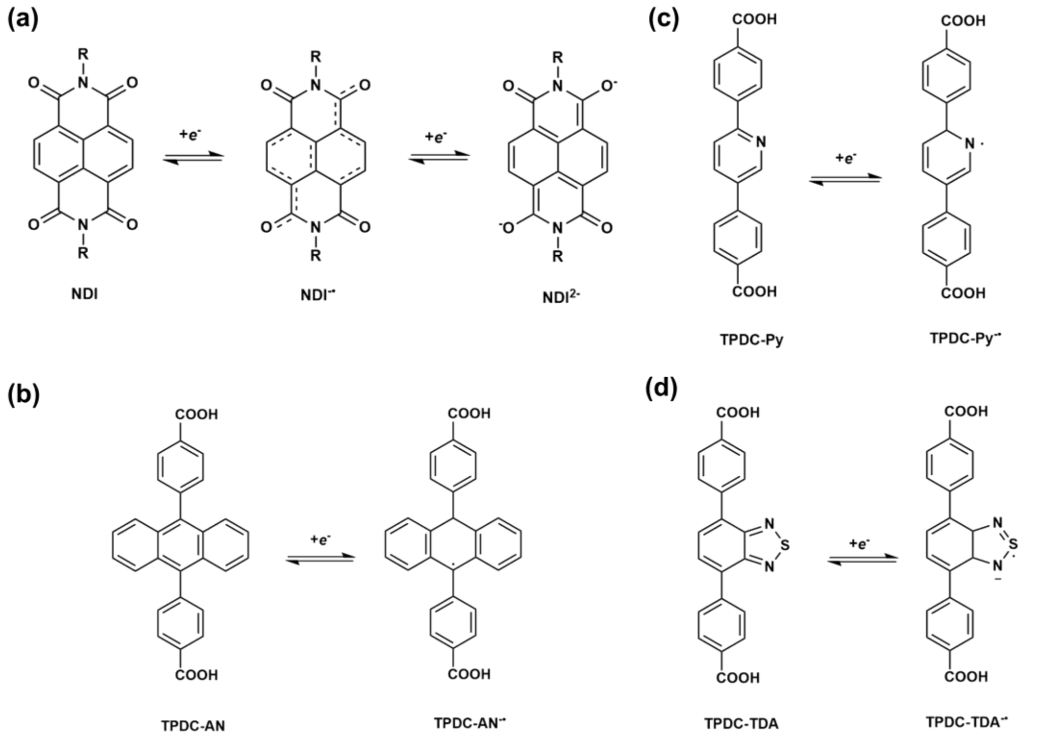

Figure S93. Proposed redox mechanisms for (a) the NDI core of H_4NDTB-R and (b-d) H_2TPDC-X (X = AN, Py, TDA).

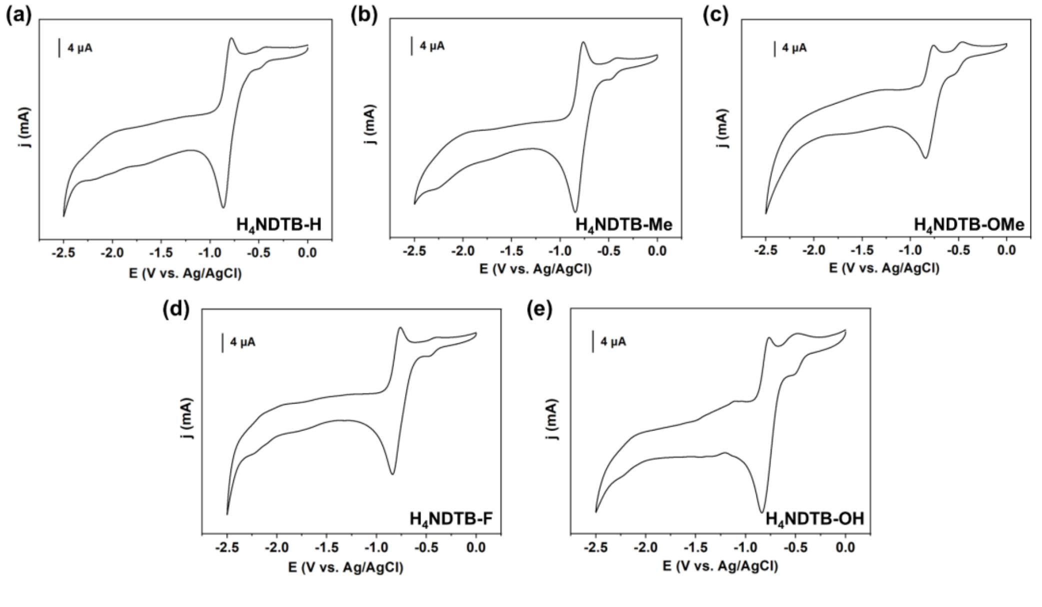

Figure S94. CV curves of five pristine H_4NDTB-R (R = H, Me, OMe,[8] F, OH) in N_2-saturated 0.1 M [(^{n}Bu)$_4$N]PF_6/DMF solution at a scan rate of 50 mV s^{-1}.

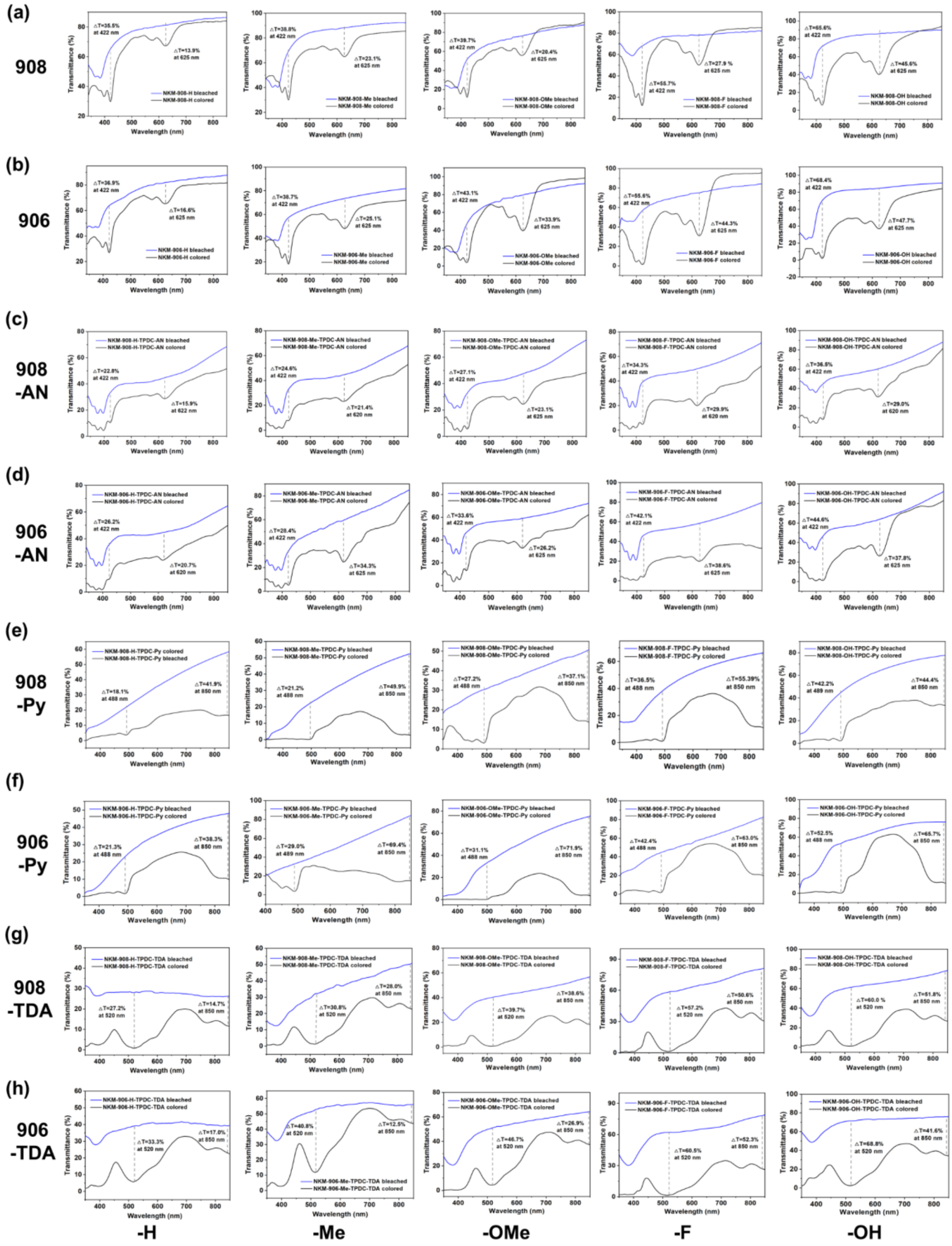

Figure S95. Optical transmittance spectra and corresponding optical contrast (ΔT) values of all forty MOF thin films over one electrochromic process. See **Table S6** for summary.

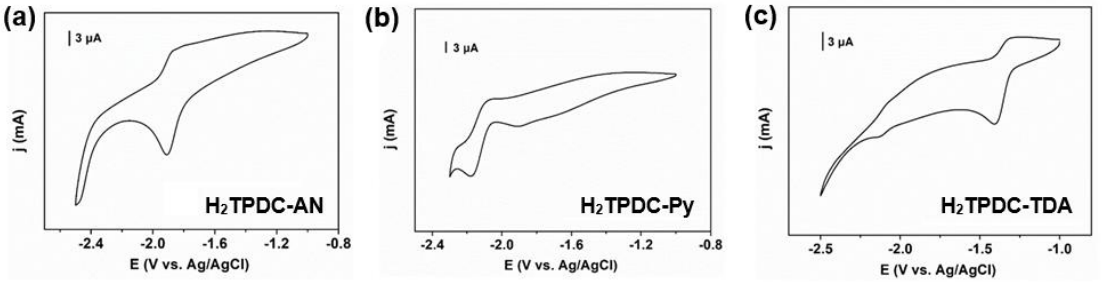

Figure S96. CV curves of three pristine H_2TPDC-X (X = AN, Py, TDA) in N_2-saturated 0.1 M $[(^{n}Bu)_4N]PF_6$/DMF solution at a scan rate of 50 mV s^{-1}.[8]

Figure S97: Photographs of the drop-cast film fabricated from using ink of organic linkers

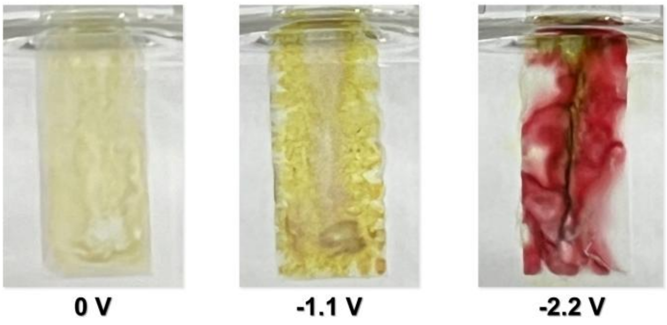

Figure S97. Photographs of the drop-cast film fabricated from using ink of organic linkers (mixture of H_4NDTB-OMe and H_2TPDC-Py) during electrochromic processes.

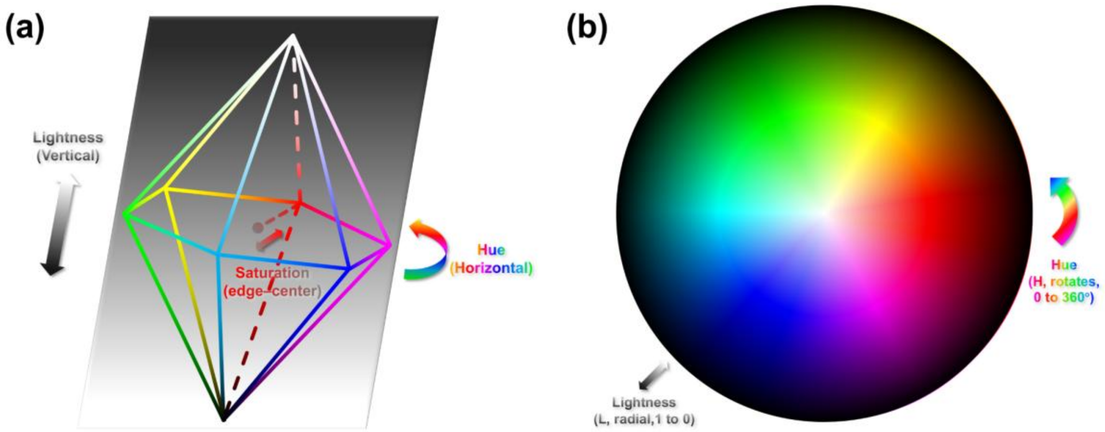

Figure S98. Illustration of the (a) double-cone model of HSL model and (b) its 2D extension of the model surface (where S = 100%).

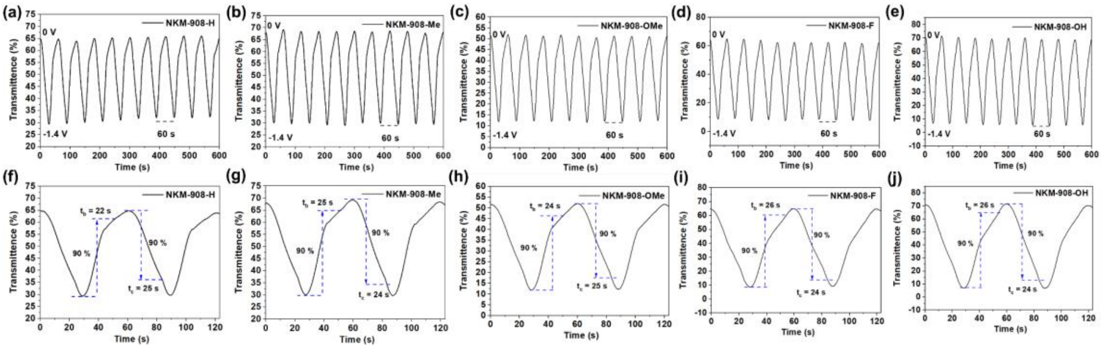

Figure S99. (a-e) Dynamic optical transmittance spectra upon applying a potential step of 0 V and -2.2 V at 422 nm (in 0.1 M [(^{n}Bu)$_4$N]PF$_6$/DMF solution), and (f-j) the corresponding coloring time (t_c) and bleaching time (t_b) for NKM-908-R.

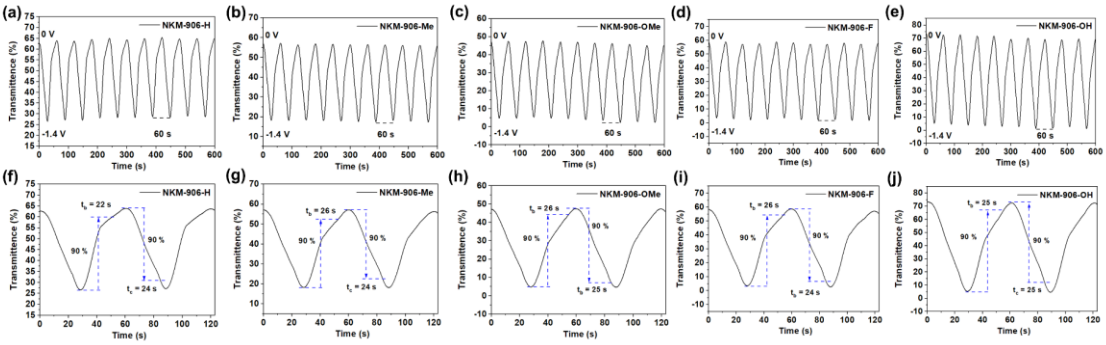

Figure S100. (a-e) Dynamic optical transmittance spectra upon applying a potential step of 0 V and -2.2 V at 422 nm (in 0.1 M [(^{n}Bu)$_4$N]PF$_6$/DMF solution), and (f-j) the corresponding coloring time (t_c) and bleaching time (t_b) for NKM-906-R.

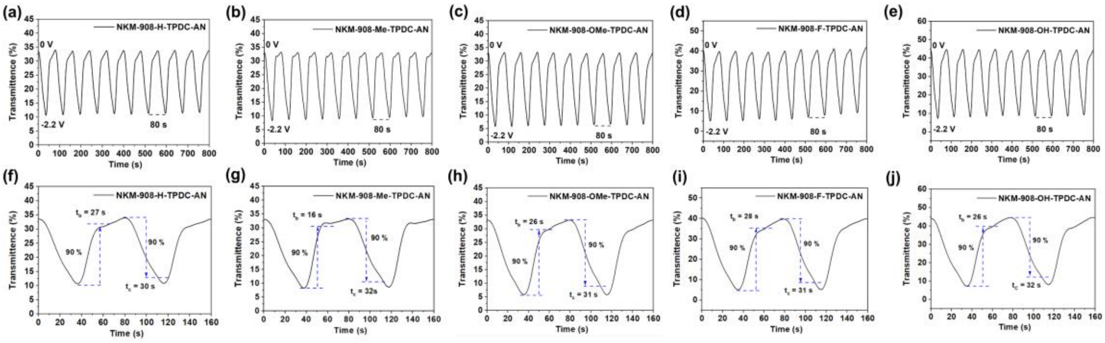

Figure S101. (a-e) Dynamic optical transmittance spectra upon applying a potential step of 0 V and -2.2 V at 422 nm (in 0.1 M [(^{n}Bu)$_4$N]PF$_6$/DMF solution), and (f-j) the corresponding coloring time (t_c) and bleaching time (t_b) for NKM-908-R-TPDC-AN.

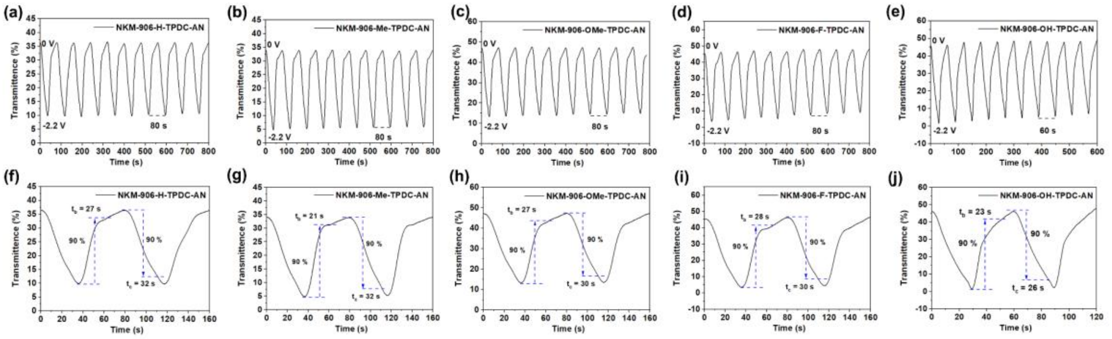

Figure S102. (a-e) Dynamic optical transmittance spectra upon applying a potential step of 0 V and -2.2 V at 422 nm (in 0.1 M [(^{n}Bu)$_4$N]PF$_6$/DMF solution), and (f-j) the corresponding coloring time (t_c) and bleaching time (t_b) for NKM-906-R-TPDC-AN.

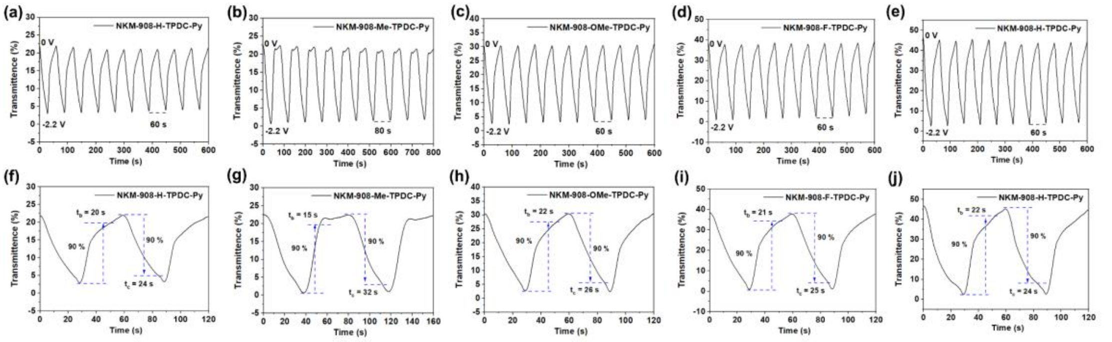

Figure S103. (a-e) Dynamic optical transmittance spectra upon applying a potential step of 0 V and -2.2 V at 488 nm (in 0.1 M [(^{n}Bu)$_4$N]PF$_6$/DMF solution), and (f-j) the corresponding coloring time (t_c) and bleaching time (t_b) for NKM-908-R-TPDC-Py.

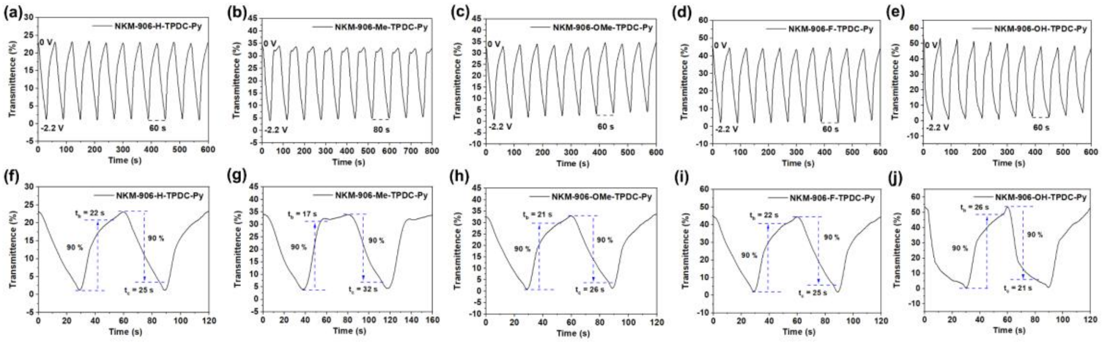

Figure S104. (a-e) Dynamic optical transmittance spectra upon applying a potential step of 0 V and -2.2 V at 488 nm (in 0.1 M [(^{n}Bu)$_4$N]PF$_6$/DMF solution), and (f-j) the corresponding coloring time (t_c) and bleaching time (t_b) for NKM-906-R-TPDC-Py.

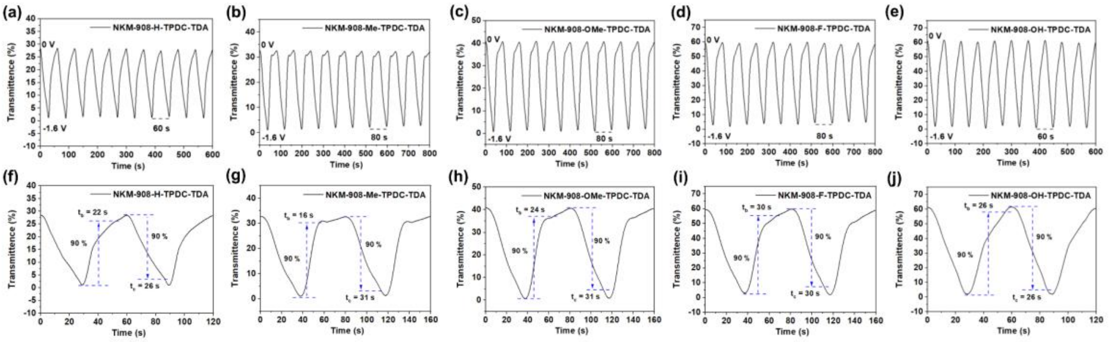

Figure S105. (a-e) Dynamic optical transmittance spectra upon applying a potential step of 0 V and -1.6 V at 520 nm (in 0.1 M $[(^{n}Bu)_4N]PF_6$/DMF solution), and (f-j) the corresponding coloring time (t_c) and bleaching time (t_b) for NKM-908-R-TPDC-TDA.

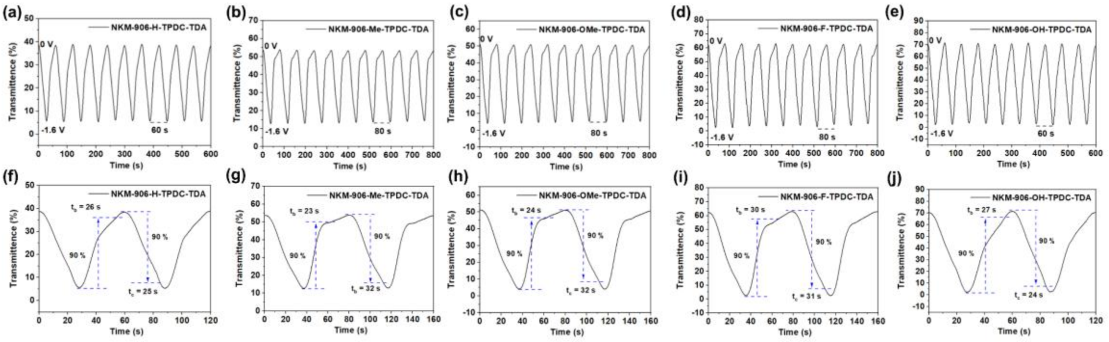

Figure S106. (a-e) Dynamic optical transmittance spectra upon applying a potential step of 0 V and -1.6 V at 520 nm (in 0.1 M $[(^{n}Bu)_4N]PF_6$/DMF solution), and (f-j) the corresponding coloring time (t_c) and bleaching time (t_b) for NKM-906-R-TPDC-TDA.

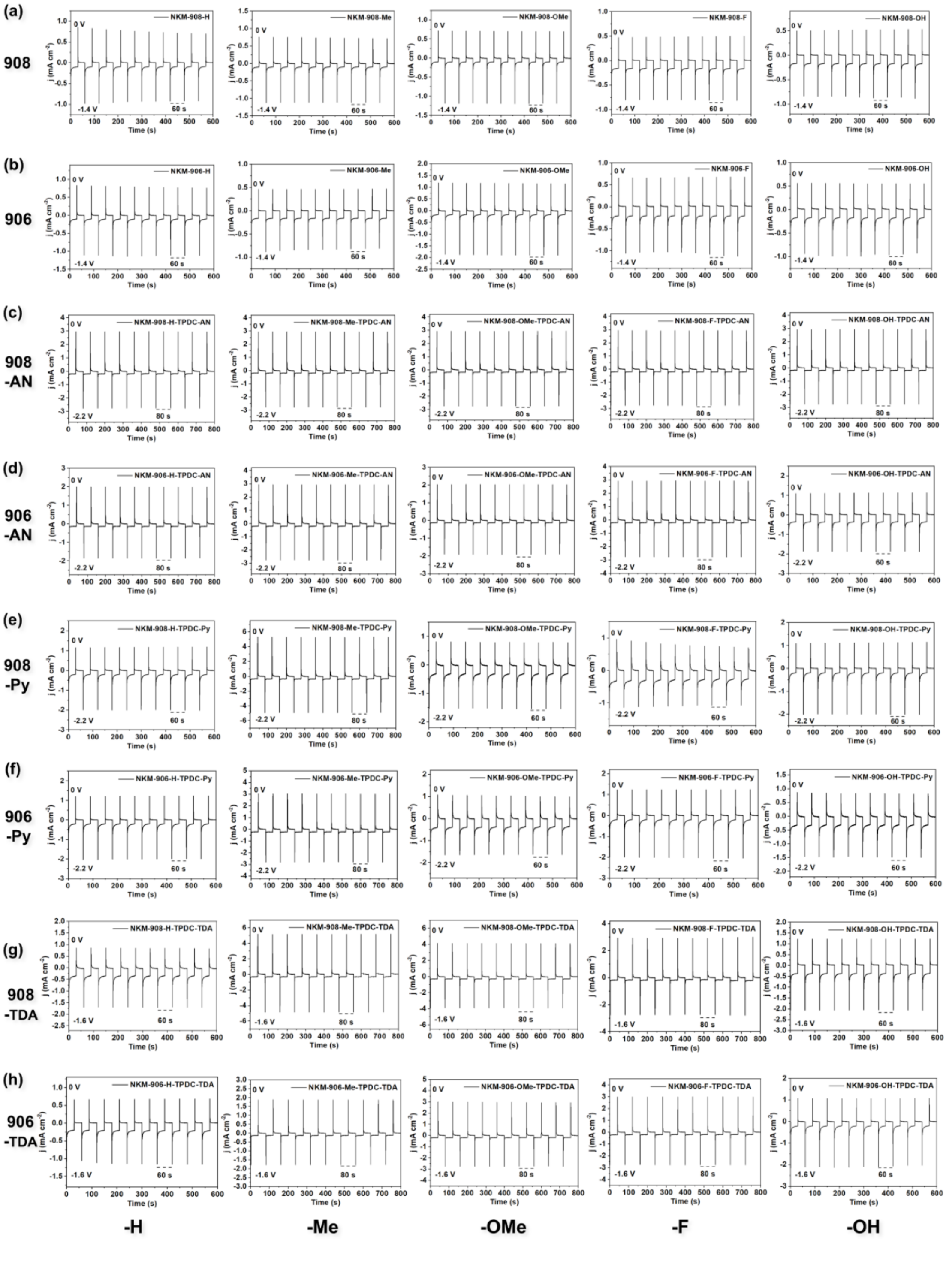

Figure S107. Changes of current density for all forty MOF thin films during over 10 electrochromic cycles.

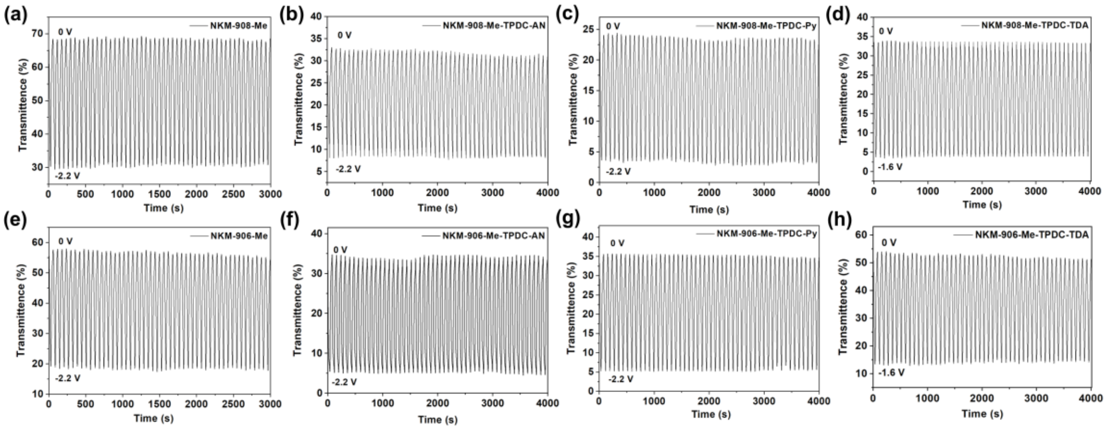

Figure S108. Dynamic optical transmittance spectra over 50 cycles for NKM-908-Me, NKM-908-Me-TPDC-X, NKM-906-Me and NKM-906-Me-TPDC-X.

Table S6. Summary of the MOF-based electrochromic materials in the literature with compassion to the forty MOFs in this work.

**MOF**	t_c/t_b **(s)**	**CE (cm^2 C^{-1})**	**ΔT (%)**	**References**
Zn-PDI	1.6/2.6	941	96.4	9
Zn-NDI	3.7/4	610	97.1	9
Zn-PMDI	N.A.	753	89.5	9
Zr-TBAPy (NU-901)	5/12	204	62	10
Zr- BINDI	N.A.	55.38	29	11
Ni/Mg-CHNDI (MOF-74 type)	7	>100	N.A.	12
Mg-PDI (MOF-74 type)	2.4/1.2	200	27.7	13
Ni-CHNDI (MOF-74 type)	2.1/1.9	260	73	14
NI- BINDI	9.5/5.1	132	42	14
Cu-TTPA	N.A.	N.A.		15
Cu-TCA	5	N.A.	65	16
Cu-HHTP	3.2/5.9	632	40.2	17
Zn (NDI-H)	~6	297	N.A.	18
Zn (NDI-ATZ)	4.35/7.72	99.14	6.45	19
Zn-NDI-74	3/91	52	32	20
Ni-MOF-74	24.5/23.5	N.A.	36	21
HKUST-1	56.5/25	N.A.	3.5	22
NKM-908-H	25/22	90.97	35.5 at 422 nm	This work
NKM-908-Me	24/25	88.02	38.8 at 422 nm	This work
NKM-908-OMe	25/24	158.99	39.7 at 422 nm	This work
NKM-908-F	24/26	161.73	55.7 at 422 nm	This work
NKM-908-OH	24/26	179.89	65.6 at 422 nm	This work
NKM-906-H	24/22	91.25	36.9 at 422 nm	This work
NKM-906-Me	24/26	90.10	38.7 at 422 nm	This work
NKM-906-OMe	25/26	151.25	43.1 at 422 nm	This work
NKM-906-F	24/26	165.50	55.6 at 422 nm	This work
NKM-906-OH	25/25	184.90	68.4 at 422 nm	This work
NKM-908-H-TPDC-AN	30/27	49.38	22.8 at 422 nm	This work
NKM-908-Me-TPDC-AN	32/16	62.18	24.6 at 422 nm	This work
NKM-908-OMe-TPDC-AN	31/26	82.27	27.1 at 422 nm	This work
NKM-908-F-TPDC-AN	31/28	91.23	34.3 at 422 nm	This work
NKM-908-OH-TPDC-AN	32/26	92.13	36.5 at 422 nm	This work

Table S6. (Continued)

MOF	t_c/t_b (s)	CE (cm^2 C^{-1})	ΔT (%)	References
NKM-906-H-TPDC-AN	32/27	84.78	22.8 at 422 nm	This work
NKM-906-Me-TPDC-AN	32/21	85.83	24.6 at 422 nm	This work
NKM-906-OMe-TPDC-AN	30/27	85.46	27.1 at 422 nm	This work
NKM-906-F-TPDC-AN	30/28	111.86	34.3 at 422 nm	This work
NKM-906-OH-TPDC-AN	26/23	121.13	36.5 at 422 nm	This work
NKM-908-H-TPDC-Py	24/20	103.27	18.1 at 488 nm	This work
NKM-908-Me-TPDC-Py	32/15	85.15	21.2 at 488 nm	This work
NKM-908-OMe-TPDC-Py	26/22	90.47	27.2 at 488 nm	This work
NKM-908-F-TPDC-Py	25/21	140.81	36.5 at 488 nm	This work
NKM-908-OH-TPDC-Py	24/22	150.45	42.2 at 488 nm	This work
NKM-906-H-TPDC-Py	25/22	144.09	21.3 at 488 nm	This work
NKM-906-Me-TPDC-Py	32/17	89.66	29.0 at 488 nm	This work
NKM-906-OMe-TPDC-Py	26/21	104.93	31.1 at 488 nm	This work
NKM-906-F-TPDC-Py	25/22	148.14	42.1 at 488 nm	This work
NKM-906-OH-TPDC-Py	21/26	161.24	52.5 at 488 nm	This work
NKM-908-H-TPDC-TDA	26/22	103.27	27.2 at 520 nm	This work
NKM-908-Me-TPDC-TDA	31/16	85.15	30.8 at 520 nm	This work
NKM-908-OMe-TPDC-TDA	31/24	90.47	39.7 at 520 nm	This work
NKM-908-F-TPDC-TDA	30/30	140.81	57.2 at 520 nm	This work
NKM-908-OH-TPDC-TDA	26/26	150.45	60.0 at 520 nm	This work
NKM-906-H-TPDC-TDA	25/26	144.09	33.3 at 520 nm	This work
NKM-906-Me-TPDC-TDA	31/16	89.66	40.8 at 520 nm	This work
NKM-906-OMe-TPDC-TDA	31/24	104.93	46.7 at 520 nm	This work
NKM-906-F-TPDC-TDA	30/30	148.14	60.5 at 520 nm	This work
NKM-906-OH-TPDC-TDA	24/27	161.24	68.8 at 520 nm	This work

References

1 Francart, T., van Wieringen, A. & Wouters, J. Apex 3: A multi-purpose test platform for auditory psychophysical experiments. *J. Neurosci. Methods.* **172**, 283-293 (2008).

2 Sadabs: Program for empirical absorption correction of area detector data (University of Göttingen, 1996).

3 Dolomanov, O. V., Bourhis, L. J., Gildea, R. J., Howard, J. A. K. & Puschmann, H. Olex2: A complete structure solution, refinement and analysis program. *J. Appl. Crystallogr.* **42**, 339-341 (2009).

4 Sheldrick, G. M. A short history of shelx. *Acta Cryst. A* **64**, 112-122 (2008).

5 Spek, A. L. Platon squeeze: A tool for the calculation of the disordered solvent contribution to the calculated structure factors. *Acta Cryst. C* **71**, 9-18 (2015).

6 Crysalispro (Oxford Diffraction /Agilent Technologies UK Ltd, Yarnton, England).

7 Macrae, C. F. *et al.* Mercury 4.0: From visualization to analysis, design and prediction. *J. Appl. Crystallogr.* **53**, 226-235 (2020).

8 Li, C. *et al.* Efficiently regulating the electrochromic behavior of naphthalene-diimide-based zirconium-organic frameworks through linker installation. *Nat. Commun.* **16**, 1405 (2025).

9 Kumar, A., Li, J., Inge, A. K. & Ott, S. Electrochromism in isoreticular metal-organic framework thin films with record high coloration efficiency. *ACS Nano* **17**, 21595-21603 (2023).

10 Kung, C.-W. *et al.* Metal–organic framework thin films composed of free-standing acicular nanorods exhibiting reversible electrochromism. *Chem. Mater.* **25**, 5012-5017 (2013).

11 Radha, G., Roy, S., Chakraborty, C. & Aggarwal, H. Electrochromic and photochromic behaviour in a single metal-organic framework containing a redox-active linker. *Chem. Commun.* **58**, 4024-4027 (2022).

12 AlKaabi, K., Wade, C. R. & Dincă, M. Transparent-to-dark electrochromic behavior in naphthalene-diimide-based mesoporous MOF-74 analogs. *Chem* **1**, 264-272 (2016).

13 Lu, Z. *et al.* Ultra-stable ionic-liquid-based electrochromism enabled by metal-organic frameworks. *Cell Rep. Phys. Sci.* **3**, 100866 (2022).

14 Li, R. *et al.* Ion-transport design for high-performance Na^+-based electrochromics. *ACS Nano* **12**, 3759-3768 (2018).

15 Ngue, C. M. *et al.* Spectroelectrochemical studies of the redox active tris[4-(triazol-1-yl)phenyl]amine linker and redox state manipulation of mn(ii)/cu(ii) coordination frameworks. *Dalton Trans.* **48**, 10122-10128 (2019).

16 Liu, J. *et al.* Highly stable and rapid switching electrochromic thin films based on metal-organic frameworks with redox-active triphenylamine ligands. *ACS Appl. Mater. Interfaces* **12**, 7442-7450 (2020).

17 Li, R., Li, S., Zhang, Q., Li, Y. & Wang, H. Layer-by-layer assembled triphenylene-based MOFs films for electrochromic electrode. *Inorg. Chem. Commun.* **123**, 108354 (2021).

18 Wade, C. R., Li, M. & Dinca, M. Facile deposition of multicolored electrochromic metal-organic framework thin films. *Angew. Chem. Int. Ed.* **52**, 13377-13381 (2013).

19 More, P. P., Rathod, P. V., Puguan, J. M. C. & Kim, H. All-in-one display device with multicolor states derived from nbu-3 MOF/monoalkylated viologen hybrid ionogel material. *Dyes Pigm.* **195**, 109730 (2021).

20 Wu, X. *et al.* A thin film of naphthalenediimide-based metal-organic framework with electrochromic properties. *J. Colloid Interface Sci.* **594**, 73-79 (2021).

21 Zhang, N. *et al.* Direct fabrication of electrochromic ni-MOF 74 film on ito with high-stable performance. *Ionics* **27**, 3655-3662 (2021).

22 Mohanadas, D., Zainudin, N. I. A. & Sulaiman, Y. A copper-based metal-organic framework/tungsten trioxide with improved coloration efficiency for electrochromic application. *Chem. Eng. J.* **428**, 130989 (2022).
